# A dataset of ant colonies’ motion trajectories in indoor and outdoor scenes to study clustering behavior

**DOI:** 10.1093/gigascience/giac096

**Published:** 2022-10-28

**Authors:** Meihong Wu, Xiaoyan Cao, Ming Yang, Xiaoyu Cao, Shihui Guo

**Affiliations:** School of Informatics, Xiamen University, Xiamen, 361000, China; School of Informatics, Xiamen University, Xiamen, 361000, China; School of Informatics, Xiamen University, Xiamen, 361000, China; Chemistry and Chemical Engineering, Xiamen University, Xiamen, 361000, China; School of Informatics, Xiamen University, Xiamen, 361000, China

**Keywords:** social insects, outdoor scenes, image sequence annotation software, computer vision, multiobject tracking

## Abstract

**Background:**

The motion and interaction of social insects (such as ants) have been studied by many researchers to understand clustering mechanisms. Most studies in the field of ant behavior have focused only on indoor environments (a laboratory setup), while outdoor environments (natural environments) are still underexplored.

**Findings:**

In this article, we collect 10 videos of 3 species of ant colonies from different scenes, including 5 indoor and 5 outdoor scenes. We develop an image sequence marking software named VisualMarkData, which enables us to provide annotations of the ants in the videos. (i) It offers comprehensive annotations of states at the individual-target and colony-target levels. (ii) It provides a simple matrix format to represent multiple targets and multiple groups of annotations (along with their IDs and behavior labels). (iii) During the annotation process, we propose a simple and effective visualization that takes the annotation information of the previous frame as a reference, and then a user can simply click on the center point of each target to complete the annotation task. (iv) We develop a user-friendly window-based GUI to minimize labor and maximize annotation quality. In all 5,354 frames, the location information and the identification number of each ant are recorded for a total of 712 ants and 114,112 annotations. Moreover, we provide visual analysis tools to assess and validate the technical quality and reproducibility of our data.

**Conclusions:**

We provide a large-scale ant dataset with the accompanying annotation software. It is hoped that our work will contribute to a deeper exploration of the behavior of ant colonies.

## Context

Social insects often tend to cluster into a colony [1], which is a complex social network [2]. From time to time, the social network springs up with self-organized clustering behaviors, including the division of labor [3], task specialization [4], and distributed problem solving [5]. Biologists have analyzed the evolution of social networks to understand the clustering behavior of insects [6], thus promoting the development of relevant modern applications, such as wireless communication [7] and cluster intelligent control [8]. The key requirement of this research is the ability to track the motions and interactions of individuals robustly and accurately.

Until the late 20th century, biologists still manually tracked motion trajectories through videos to guarantee the accuracy of markings. However, they had to track each individual at a time, which means the entire video needed to be watched 50 times or more in the case of crowded scenes [9]. Manual tracking is time-consuming and prone to human error. It becomes an inhibiting factor in obtaining a complete and accurate dataset required to analyze the evolution of social networks. Therefore, in the past 2 decades, attempts have been made to automate the tracking process for social insects utilizing computer vision (CV) techniques [10–14].

Traditional CV techniques release researchers from manual work through approaches such as the foreground segmentation algorithm [15], temporal difference method [10], and Hungarian algorithm [16]. Such approaches, however, have failed to address noise in images [17]; hence, these approaches are limited to laboratory environments with clean backgrounds. Nevertheless, many scientifically valuable results are obtained in nature rather than in laboratory environments [18–21].

Fortunately, with the emergence of deep learning, CV techniques are already capable of addressing many complex tasks [22–24], which is beneficial to automated insect tracking in outdoor scenes. Several studies have explored automated multiant tracking in outdoor scenes using deep learning–based models [25, 26]. The experimental results demonstrate that these models could be scaled up into a cost-effective alternative to traditional manual tracking methods, which are typically costly and/or labor intensive [25, 26]. A critical requirement for the development of these models is access to datasets containing annotations of motion trajectories of insects in the video. Several works have attempted to improve the imaging of such insects in natural environments [25, 27]. To the best of our knowledge, however, only a few works [25,26] annotate motion trajectories in videos, and both use only a single outdoor scene sequence, which lacks data diversity.

Considering the importance of annotating targets in videos, some annotation tools have been proposed over the years, including LabelME [28], VATIC [29], ViPER [30], and ViTBAT [31]. Except for ViTBAT, other tools are generally more suitable for annotating ground-truth information at the individual target level in terms of tracking targets. ViTBAT supports annotating a group of targets but requires much effort to set up rectangular boxes with different sizes for each target. Additionally, it cannot display the annotation results of the previous frame in the current frame, which makes it difficult for a user to identify the same target during the annotation process of a video sequence. Moreover, it is only supported in Linux systems, which are difficult to use for biology researchers without a computer background. In our opinion, a marking tool should be user-friendly, minimize human effort, and maximize annotation.

To summarize, the proposed tool and dataset are the main contributions of our work.

With respect to the tool, we propose VisualMarkData, which allows users to generate ground-truth information of multitarget motion trajectories in video sequences. Specifically, VisualMarkData offers (i) a comprehensive annotation of states at the individual-target and group-target levels; (ii) representation of annotations (together with their IDs and behavior labels) of multiple targets and multiple groups in a simple-to-access matrix format; (iii) a simple and efficient visualization during annotation, which presents the annotation information of the previous frame as a reference and then only requires clicking on the center point of each target to complete the annotation; and (iv) a Windows-based friendly graphical user interface that minimizes labor and maximizes annotation quality.

**Figure 1:**
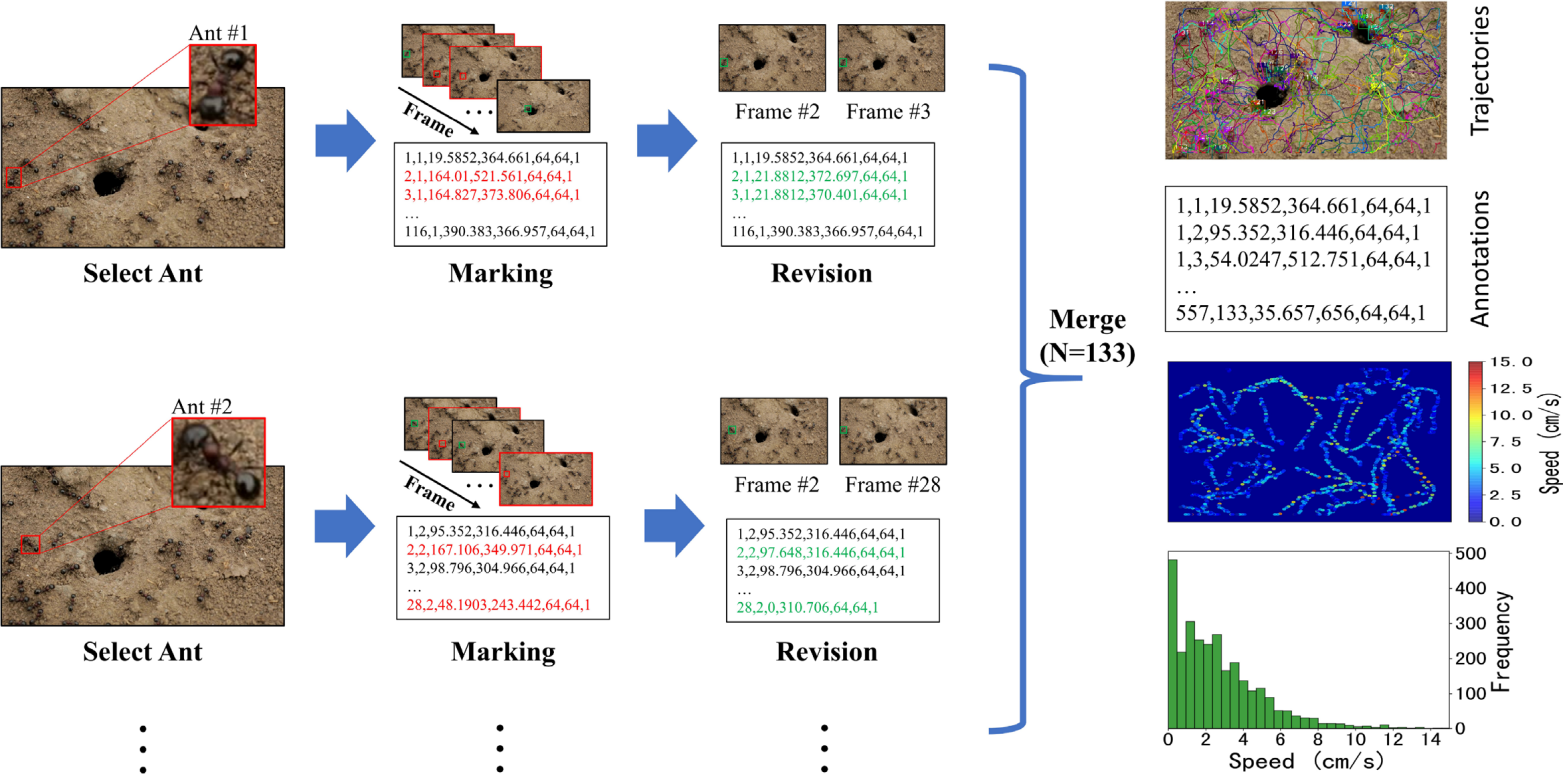
The pipeline for marking motion trajectories of ants in an image sequence; an outdoor scene is taken as an example. A total of 133 ants appear in this image sequence, and we select 1 ant to be marked in each epoch. We use a square bounding box to indicate the ant’s location and record the relevant parameters at the same time. After all ants of the entire image sequence have been marked, we check the quality of the annotations frame by frame so that wrong annotations (red font) can be corrected (green font). Then, we merge all the annotations of the image sequence into one file. Additionally, 3 Python scripts are provided to generate 3 visualization results to verify the quality of the data, including the trajectories drawn on the original graph, the heatmap of motion speeds, and the histogram of the frequency distribution of motion speeds.

With regard to the dataset, to our knowledge, we are the first to construct an ant colony activity dataset with annotations that includes multiple species and colonies in both indoor and outdoor environments. Concretely, we build equipment for video acquisition in various environments and obtain a number of different ant colony activity videos that include 3 species and 10 colonies. Then, utilizing VisualMarkData and following the process shown in Fig. 1, a large-scale dataset of ant colony activity with annotations is constructed. The total size of the dataset is 5,354 frames, 712 ants, and 114,112 labels. We believe that the dataset will benefit future research on social insect behavior analysis.

## Data description

We collect 10 videos that record the activities of different ant colonies, including colonies from both indoor and outdoor scenes. To help us mark the motion trajectories, we develop an image sequence marking software called VisualMarkData.

After spending a large quantity of time and effort, we obtain a dataset with 5,354 frames and 114,112 annotations. Table 1 describes the dataset in detail.

**Table 1:** Descriptions of ant videos with annotations in indoor and outdoor scenes. The top part provides the filming details. Sequence = Name of video for each colony. Angle = Horizontal angle of the camera during filming. Height = Height of the camera from the ground. Temp = Local temperature during filming. Datetime = Date and time of filming. Location = Location of filming. Camera = Camera type. The bottom part provides a description of the ant videos with annotations. FPS = Frame rate of the video. Resolution = Resolution of the video. Length = Number of frames in the video, with the duration in parentheses. Ants = Number of ants with different IDs that appear in the video. Annotations = Number of ant instances labeled in the video. Species = Ant species. Entrance = Whether the colony is active at the nest entrance. Area = Area of the filmed scene. Note that the camera’s angle of view is 16^°^ and 7.5^°^ in the horizontal and vertical directions, respectively, which are not represented in the table.

Filming details									
Scene	Sequence	Angle	Height	Temp	Datetime	Location		Camera	
Indoor	Seq0001	0^°^	30 cm	24^°^C–26^°^C	2019/04/15 morning	Xiamen, Fujian, China		Panasonic GX 85	
	Seq0002	0^°^	30 cm	24^°^C–26^°^C	2019/04/15 morning	Xiamen, Fujian, China		Panasonic GX 85	
	Seq0003	0^°^	30 cm	24^°^C–26^°^C	2019/04/15 morning	Xiamen, Fujian, China		Panasonic GX 85	
	Seq0004	0^°^	30 cm	24^°^C–26^°^C	2019/04/15 morning	Xiamen, Fujian, China		Panasonic GX 85	
	Seq0005	0^°^	30 cm	24^°^C–26^°^C	2019/04/15 morning	Xiamen, Fujian, China		Panasonic GX 85	
Outdoor	Seq0006	45^°^	30 cm	15^°^C–18^°^C	2019/06/23 morning	Saint-Petersburg, Russian Federation		Canon 5d	
	Seq0007	30^°^	30 cm	30^°^C–35^°^C	2019/07/21 morning	Athens, Greece		Canon 5d	
	Seq0008	30^°^	30 cm	15^°^C–18^°^C	2019/06/23 morning	Saint-Petersburg, Russian Federation		Canon 5d	
	Seq0009	30^°^	30 cm	15^°^C–18^°^C	2019/06/23 morning	Saint-Petersburg, Russian Federation		Canon 5d	
	Seq0010	0^°^	30 cm	15^°^C–17^°^C	2019/04/21 morning	Neptune Beach, United States		Canon T3i	
Description of videos with annotations									
Scene	Sequence	FPS	Resolution	Length	Ants	Annotations	Species	Entrance	Area
Indoor	Seq0001	25	1,920 × 1,080	351 (00:14)	10	3,510	Japanese arched ants	no	17 cm × $8\, \mathrm{cm}$
	Seq0002			351 (00:14)	10	3,510	Japanese arched ants	no	17 cm × $8\, \mathrm{cm}$
	Seq0003			351 (00:14)	10	3,510	Japanese arched ants	no	17 cm × $8\, \mathrm{cm}$
	Seq0004			351 (00:14)	10	3,510	Japanese arched ants	no	17 cm × $8\, \mathrm{cm}$
	Seq0005			1,001 (00:40)	10	3,510	Japanese arched ants	no	17 cm × $8\, \mathrm{cm}$
Outdoor	Seq0006	30	1,280 × 720	600 (00:20)	73	11,178	Carpenter ants	yes	17 cm × $16\, \mathrm{cm}$
	Seq0007			677 (00:23)	162	25,158	Little black ants	yes	17 cm × $11\, \mathrm{cm}$
	Seq0008			577 (00:19)	133	10,280	Carpenter ants	yes	17 cm × $11\, \mathrm{cm}$
	Seq0009			526 (00:18)	193	27,902	Carpenter ants	yes	17 cm × $11\, \mathrm{cm}$
	Seq0010			569 (00:19)	101	2,044	Little black ants	no	17 cm × $8\, \mathrm{cm}$

### Data acquisition

#### Indoor environment

Japanese arched ants (also called *Camponotus japonicus*; NCBI:txid84547) are widely studied by behavioral ecologists and social biologists [32–34]. These ants are often domesticated; thus, they are suitable for observation in laboratory environments. We collected 50 Japanese arched ant workers, which ranged from 7.4 to 13.8 mm in body length [35]. We constructed a laboratory environment that included a stable light source, stable temperature, and a transparent plastic container. The background of the container was clean and did not contain the nest. We randomly divided them into 5 colonies of ants. Then, we loaded each colony into the container in turns and filmed their activities with a high-resolution video camera. These videos were named Seq0001 to Seq0005. These recordings took place on April 15, 2019, in the morning in Xiamen, Fujian, China. More detailed information is provided in Table 1.

#### Outdoor environment

Little black ants (*Solenopsis invicta*; NCBI:txid13686) [36, 37] and carpenter ants (*Camponotus herculeanus*; NCBI:txid36169) [38–40] have been the focus of research by behavioral ecologists and sociobiologists. We acquired 5 videos from 5 ant colonies in different outdoor environments; each colony contained 73 to 193 workers. The species of these ant colonies were carpenter and little black ants, and their body lengths were between 8 and 10 mm  [41]. We named the obtained videos Seq0006 to Seq0010. Concrete and uneven stones were in the background of Seq0006. Seq0007 and Seq0008 were filmed in dry grass scenes. Seq0009 and Seq0010 were filmed on a dirt road and a rocky road, respectively. The backgrounds of the scenes were not processed. Except for Seq0010, the scenes of the other 4 videos were taken at the entrance of the nest. More informative details about the time, location, and temperature of each scene are shown in Table 1.

### Data records

The dataset consists of 10 image sequences from different scenes in JPEG digital image format, which is published in the ANTS–ant detection and tracking repository [42, 43]. In addition, we provide annotations created by VisualMarkData for all image sequences in the form of text. In the dataset, the images and annotations of each sequence are organized into three folders named “det, “gt,” and “img.”

#### Det folder

In the same format as the dataset of the multiobject tracking challenge [44], we record information, such as the identity and location parameters of all ants, in each frame for detection. Such information is stored in a “det.txt” file in a folder named “det” in our dataset. Concretely, each line represents 1 ant instance, and it contains 7 values (also called attributes), as shown in Table 2. The first number indicates in which frame the ant appears (sorted by ascending order), while the second number identifies that ant as belonging to a trajectory by assigning a unique ID (set to −1 in the detection file, as no ID is assigned yet). The next 4 numbers indicate the location of the bounding box of the ant in 2-dimensional image coordinates. The location and the width and height of the bounding box are indicated in the top-left corner. This is followed by a single number, which denotes the confidence score.

**Table 2:** Data format of the “det.txt” and “gt.txt” annotation files

Position	Name	Description
1	Frame number	Indicate in which frame the object is present
2	Identity number	Each ant trajectory is identified by a unique ID (−1 for detections)
3	Bounding box left	Coordinate of the top-left corner of the ant bounding box
4	Bounding box top	Coordinate of the top-left corner of the ant bounding box
5	Bounding box width	Width in pixels of the ant bounding box
6	Bounding box height	Height in pixels of the ant bounding box
7	Confidence score	Indicates how confident the detector is that this instance is an ant.
		For the ground truth and results, it acts as a flag whether the entry is to be considered

#### Gt folder

In our dataset, we provide ground-truth records for multiobject tracking. This information is stored in a “gt.txt” file in a folder named “gt.” Similar to the previous description of the “det.txt” file, the records of each instance in the “gt.txt” file also contain 7 values (also called attributes); see Table 2 for details. Different from the “det.txt” file, the second number in the “gt.txt” file represents the ID of an ant belonging to a trajectory, which is key information for implementing multiant tracking. In addition, each ant can be assigned to only 1 trajectory.

#### Img folder

In our dataset, we provide the original image sequence converted from the video, which is stored in the “img” folder. All images are converted to JPEG and named sequentially with a 6-digit file name (e.g., 000001.jpg).

## Data validation and quality control

### Visual confirmation

For the 10 videos, 2 staff marked the indoor videos and 3 staff marked the outdoor videos. Furthermore, the ground-truth annotations for all image sequences in the dataset were visually confirmed by 1 staff member. The visual review consists of 2 aspects: sequence level (coarse-grained) and image level (fine-grained).

**Figure 2:**
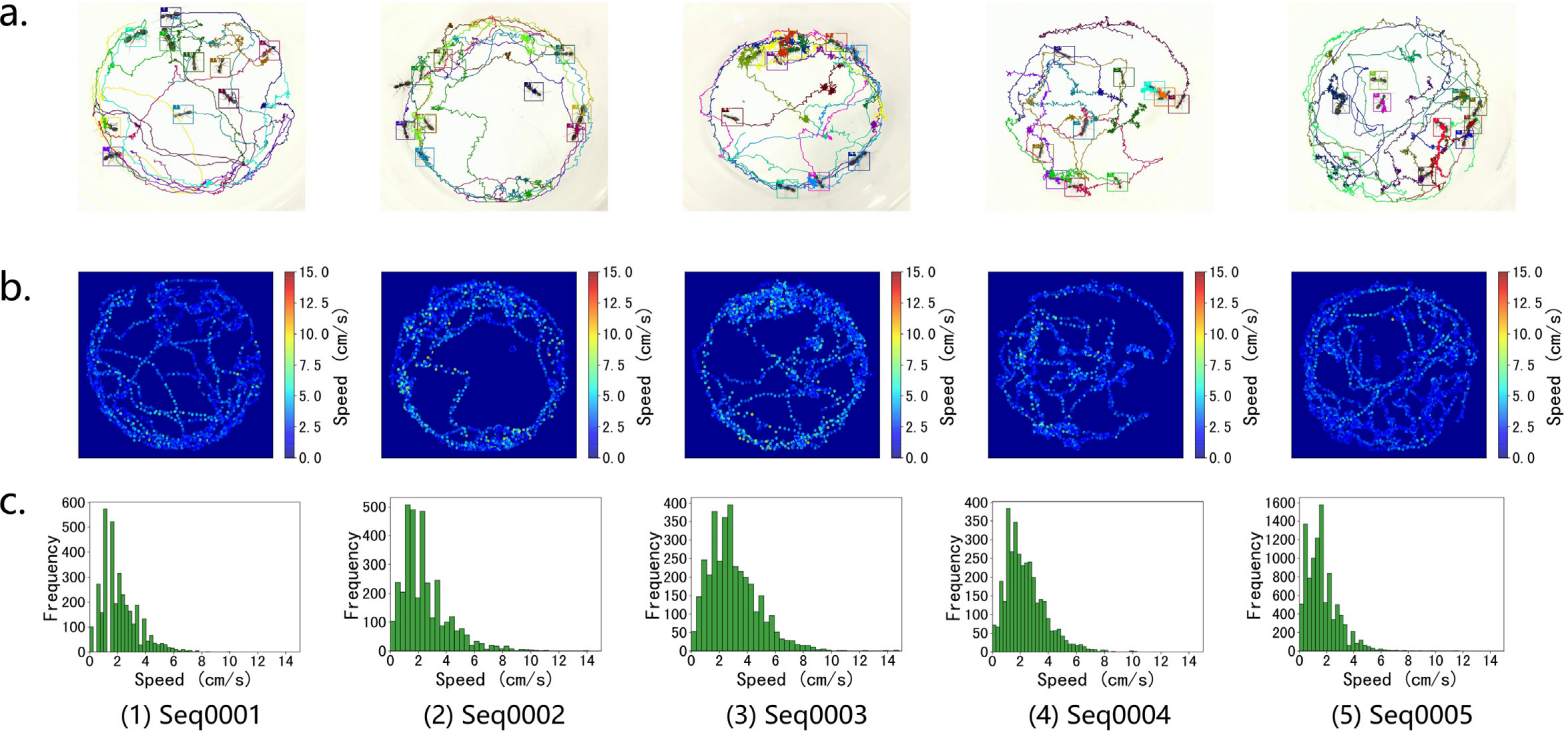
Visual analysis of the marking results on the indoor ant videos. (a) Visualization of motion trajectories of the ants for each sequence of the indoor scene. (b) Speed distributions in the image space for 5 sequences of indoor scenes. (c) Histogram of the frequency of ant speeds in $\mathrm{cm\, s}^{-1}$ for indoor sequences.

**Figure 3:**
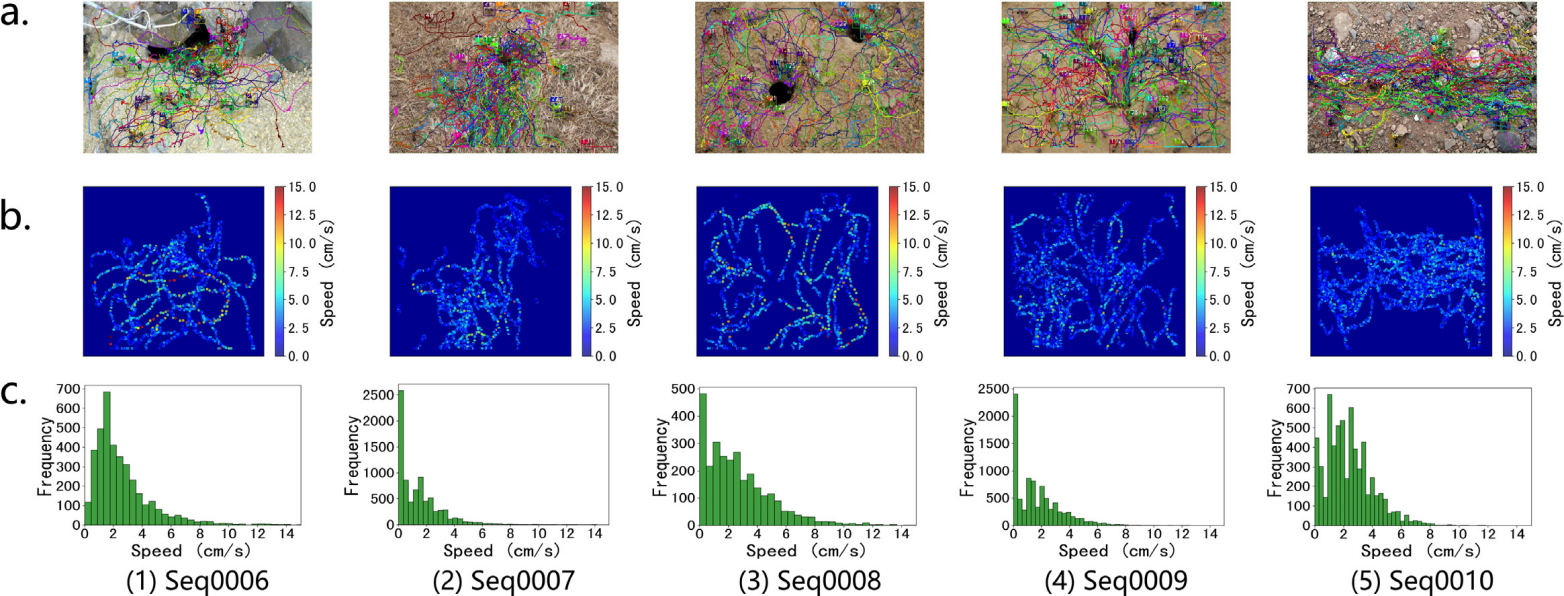
Visual analysis of the marking results on the outdoor ant videos. (a) Visualization of motion trajectories of the ants for each sequence of the outdoor scene. (b) Speed distributions in the image space for 5 consecutive sequences of indoor scenes. (c) Histogram of the frequency of ant speeds in $\mathrm{cm\, s}^{-1}$ for indoor sequences.

**Figure 4:**
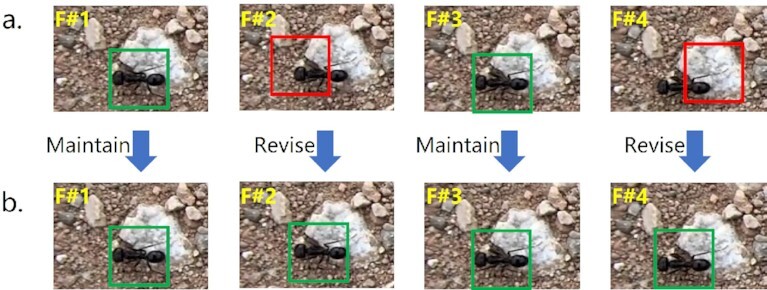
An example of remarking a segment. (a) The result of marking before revision, where the green and red boxes indicate high- and low-quality annotations, respectively. (b) The result after remarking, where we only need to revise the low-quality annotations in (a) to obtain high-quality annotations (green box).

First, the staff performed a coarse-grained review of a single sequence. Specifically, we drew the annotations on the corresponding images and then converted the image sequence into a video. For each scene, an example image frame is shown in Figs. 2a and 3a. By replaying the video, staff can quickly confirm which segments of the video are of poor quality and need to be remarked. Fig. 4a shows an example of a segment distinguished as having low-quality annotations. The sequence-level verification time consumption per video is 8 to 10 times the original video sequence duration, and it depends on the number of ants in the video. After that, staff reviewed the quality of annotations frame by frame via VisualMarkData. For inaccurate annotations, staff manually modified the annotations by using the “Check and modify” function of VisualMarkData (see details in Methods). Fig. 4b shows the modified annotations. The image-level checking speed is approximately 0.5 seconds per ant instance, while correction takes approximately 2 seconds per ant instance.

### Motion speed analysis

Furthermore, to demonstrate the reliability of our dataset, we analyzed the distribution of the movement speed of the ants in our dataset. First, for each ant, we used the 2-dimensional Euclidean distance [45] to calculate its pixel distance between 2 adjacent frames. Therefore, the pixel distance △*ps_t_* of the ant in frame *t* can be defined by the following equation:

(1) \begin{equation*} \triangle ps_{t}=\sqrt{{(px_{t} - px_{t-1})^2}+{(py_{t} - py_{t-1})^2}}
\end{equation*}

where *px_t_* denotes the pixel position of the ant in the horizontal direction at frame *t*. Similarly, *py_t_* denotes the pixel position in the vertical direction. To convert the pixel distance to real-world coordinates, we divided the ant’s body length *L* (unit: *m*) in the real world by body length *n* (unit: *pixel*) in the image.

Thus, the real-world displacement of the ant at frame *t*, △*s_t_* (unit: *m*), can be expressed as follows:

(2) \begin{equation*} \triangle s_{t} = \triangle ps_{t} \times L/n
\end{equation*}

Since the frames per second (FPS) for a specific video is a constant *f_c_*, the speed *v_t_* (unit: *m* · *s*^−1^) at frame *t* can be formulated as

(3) \begin{equation*} v_{t} = \frac{\triangle s_{t}}{1/f_{c}}
\end{equation*}

where *v*_0_ is set to 0; that is, we assumed that the ants were stationary at the initial moment. According to the aforementioned equations, combined with the location information of ants in the annotations, we can analyze the motion speed of ants in the video, as shown in Figs. 2b, c and 3b, c. Specifically, the overall motion speeds of ants in indoor and outdoor scenes are 2.16 ± 1.49 $\mathrm{cm\, s}^{-1}$ and 1.98 ± 1.84 $\mathrm{cm \, s}^{-1}$, respectively. These values are within a reasonable range (the average motion speed of ants is 2.85 $\mathrm{cm \, s}^{-1}$ under bidirectional traffic conditions [46]). This demonstrates that the ant colony activity dataset we collected and marked is real and reliable.

## Discussion

The image sequence marking software, VisualMarkData, is a toolkit with interactive visualization. The goal of the software is to provide a convenient tool for researchers to annotate the movement trajectories of social insects in videos, thus facilitating the study of the behavioral mechanisms of social insects. Additionally, by using the software, researchers can obtain standardized annotation data, as detailed in the previous section. VisualMarkData is open source, so researchers can apply it to a multiobject motion image sequence dataset. Moreover, we have provided publicly available Python Scripts at  https://github.com/holmescao/ANTS_marking_and_analysis_tools to illustrate the analysis of data as well as the usage of the data. To visualize and reproduce the results described in the Data validation and quality control section, we develop 2 scripts for the researchers. Additionally, we provide another script to calculate the metrics [44] of multiobject tracking to enable any deep learning algorithm to evaluate the tracking accuracy on the dataset. The annotated trajectory data can be used for training and testing supervised learning models, thus providing powerful tools for studying a wider range of ant colony behaviors.

In the future, it is possible that the VisualMarkData software will be updated to reduce the difficulty and improve the efficiency of annotation. The software currently marks targets based on their center points, and we are considering introducing stretchable annotation capabilities based on rectangles or ellipses. In addition, the simultaneous annotation of multiple targets in 1 frame is also a feature worth developing. Along with that, we can introduce semiautomated annotation (i.e., embedding a neural network model into VisualMarkData to automatically predict and annotate objects of the current frame based on the information in the previous frame). Thus, annotators will only need to fine-tune the annotations, which will significantly improve the efficiency of the annotation processes.

The dataset and VisualMarkData will encourage researchers in both biology and computer science to study the behavior of social insects in different environments. We hope that this work will contribute to the potential discovery of ant colony behavioral mechanisms and facilitate the application of the image processing field in biology.

## Potential usage of the dataset

Swarming behavior is one of the most important features of social insects [1] and often involves the division of labor [3], task specialization [4], and distributed problem solving [5]. Revealing the mechanisms behind swarming behavior requires observing insect colonies over long periods of time as well as recording the motion trajectory of each individual [9]. Before the advent of computer vision technology, biologists utilized manual tracking to study insect behaviors [47, 48]. Since manual recording is time-consuming and laborious, biologists focus only on individual behavioral studies, including foraging activity [48] and prey avoidance [47]. In recent years, to enable the rapid tracking of the activities of multiple insects simultaneously, automated image-based tracking techniques have been employed, and many attempts have been made to improve the accuracy of tracking [10–14]. These techniques have assisted biologists in discovering some colony mechanisms. For example, Balch et al. [2] found that a number of ants would interact at the entrance of the nest when some have found food nearby. However, current studies are limited to laboratory settings with clean backgrounds. Such approaches disregard the influence of environments surrounding insect colonies, including potential predators [49] and obstacles in the path [50]. In contrast, we provide labeled motion trajectories of active outdoor ant colonies with a variety of scenes. These data can be used to train deep learning models for the automated tracking of ants in natural environments. Moreover, we already used indoor/Japanese arched ant images as the training set in our previous work [26] and tested our model on outdoor/black ant images (Seq0010), and we achieved a tracking accuracy up to 92%. Conversely, we also conducted experiments using outdoor images as the training set and indoor images as the test set, which are presented in a method manuscript that we are preparing [51]; this manuscript can be found at arXiv. Hence, it will help biologists quantify and analyze the foraging patterns of ant colonies, such as foraging strategies, partner gathering, and collaborative transportation, in natural environments.

## Methods

### Hardware devices for acquiring raw data

#### Indoor environment

**Figure 5:**
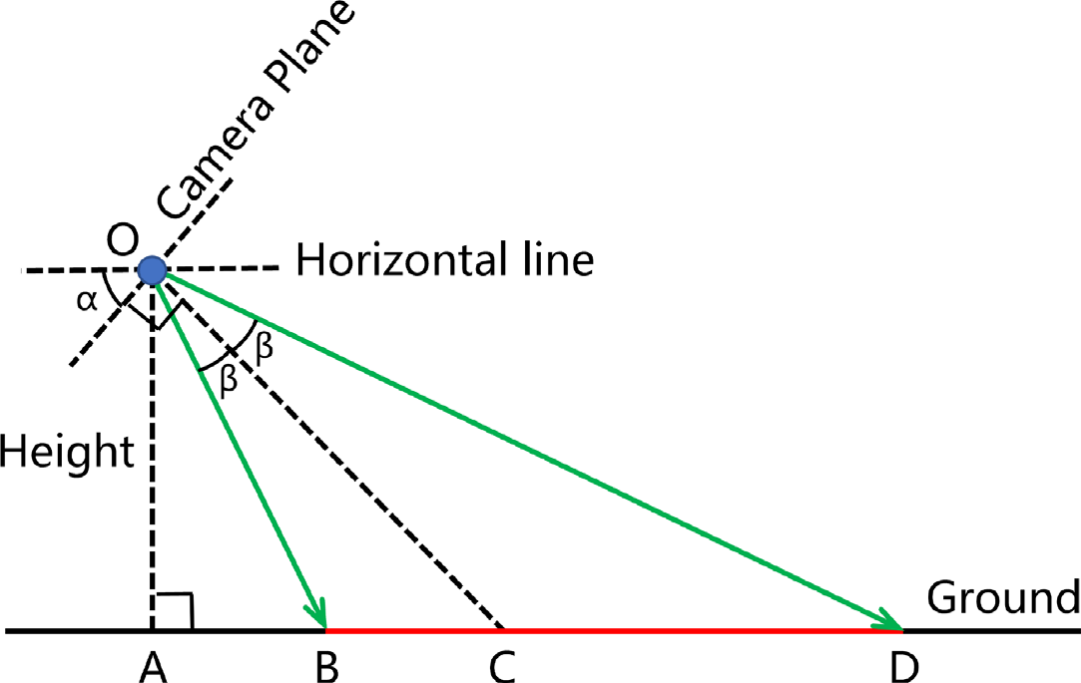
Illustration of the camera filming scene. The horizontal direction is shown as an example. The camera is at point O, the height from the ground is OA (denoted by Height), the angle between the filming angle and the horizontal line is α, and the camera’s angle of view is β. Thus, according to the position of the angle of view extending to the ground (green arrow line), the horizontal filming range can be determined and is denoted by the line BD (red line segment).

For the indoor environment, we used a cylindrical container made of transparent plastic to provide a space for the ants to move around. This container has a bottom diameter of $10\, \mathrm{cm}$, has a side height of $15\, \mathrm{cm}$, and is not closed at the top. The ants in the container were filmed with a high-resolution video camera (Panasonic GX 85) with 25 FPS in the format H.264 with a resolution of 1,920 × 1,080 pixels. To ensure stable filming, we fixed the camera on a tripod and hung a light bulb above the container. The height of the camera from the bottom of the container was 30 cm, and the filming angles in both the horizontal and vertical directions were 0^°^. Additionally, the camera had an angle of view of 16^°^ and 7.5^°^ in the horizontal and vertical directions, respectively. Fig. 5 presents an illustration filming scene, and the line segment BD denotes the length or width of the filming scene. As a result, we can use the known information to infer the value of line segment BD, as shown in Equation 4. Furthermore, we can easily obtain that the area of the indoor scene is 136 cm^2^ (17 cm × 8 cm). In addition, anti–dusting powder was applied to the inner wall of the container to prevent ants from escaping from the container during filming.

#### Outdoor environment

For the natural environments, there was no processing of the backgrounds of the scenes. The camera type was mainly a Canon 5d, which has a resolution of 1,280 × 720 with a frame rate of 30 FPS. The height of the camera from the ground was 30 cm. In different scenes, the horizontal filming angles were different (as shown in Table 1), while the vertical filming angles were all 0^°^. Additionally, the angle of view of the camera in the horizontal and vertical directions was 16^°^ and 7.5^°^, respectively. Moreover, according to Fig. 5 and Equation 4, we can calculate the area of each outdoor scene, and the concrete values are shown in Table 1.

(4) \begin{eqnarray*}
BD &=&AD-AB \nonumber \\&=&OA \times \tan \angle AOD - OA \times \tan \angle AOB \nonumber \\&=&OA \times ( \tan \angle AOD - \tan \angle AOB ) \nonumber \\&=&OA \times ( \tan ( \angle AOC + \angle COD )-\tan ( \angle AOC - \angle BOC ) ) \nonumber \\&=&Height \times ( \tan ( \angle \alpha + \angle \beta ) - \tan ( \angle \alpha - \angle \beta ) ) \end{eqnarray*}

### Description of the VisualMarkData marking software

**Figure 6:**
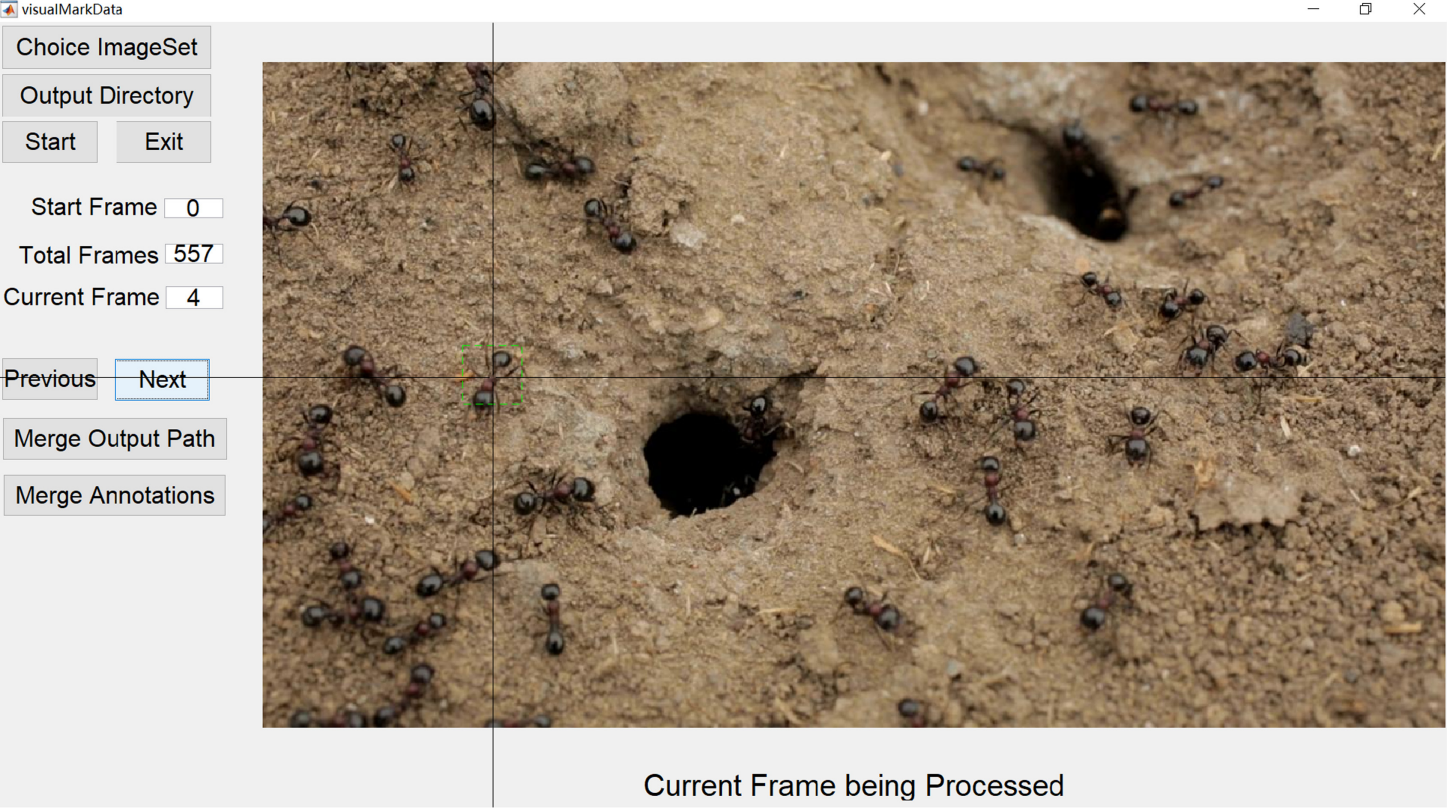
The interactive interface of our image sequence marking software, named VisualMarkData. After selecting an image sequence, a user can acquire the annotation by clicking on the center location of the ant’s body.

We developed an image sequence marking software called VisualMarkData to provide the locations and identification numbers of objects in a sequence for motion analysis. The overall annotation pipeline for the dataset using this software is shown in Fig. 1. The operation procedure of VisualMarkData is as follows, and its interface is shown in Fig. 6.

- **Choose Image Set**. Before marking, the user should click “Choose ImageSet” to select an image set. The filename of the image set is defined in the format of “SeqXObjectYImageZ,” where X is the name of the sequence, Y is the number of objects in the first frame ,and Z is the size of the bounding box that represents the object. For example, the image set, named “Seq0001Object10Image94”,,indicates that sequence “0001” contains 10 objects in the first frame, and each object will be marked with a bounding box of size 94 × 94.
- **Create Output Directory**. The user needs to click “Output Directory” to select the storage path for the annotations. Since VisualMarkData oocuses on only 1 object per marking round (each round goes through the whole image sequence), the output folder is suggested to be named with the identification number of the object (e.g., “0001”). As the identity number of the object is user-defined, the user can use any number for the object and folder as long as it is unique.
- **Select Start Frame**. In the last step before marking begins, the user needs to enter the start frame, and the default value is 0. This means that the user is allowed to exit the software halfway through the process and continue the process of the current marking task at a later time. Then, the user can click the “Start” button.
- **Marking**. The user clicks on the center of an object in the current frame, and the software will automatically save the digital location of the center, as well as a bounding box centered on the object. It should be emphasized that the user only marks the same object until the entire image sequence is finished, and then the user can focus on another object by repeating the same operation.
- **Next Frame**. The user clicks the “Next” button to show the next frame on the window of the software. The marked location from the previous frame will be displayed with a green dot, which can help the user quickly locate the target object.
- **Previous Frame**. If the marked location of the previous frame is incorrect, the user can click the “Previous” button to roll back 1 frame.
- **Check and Modify**. After the user finishes marking the entire image set, verification is needed to guarantee quality. In this case, the user can enter a specific frame to modify the annotations by carrying out the **Select Start Frame** step.
- **Merge Annotations**. After all objects in a sequence have been marked and reviewed, the user needs to click the “Merge” button; then, all annotations for each object will be sorted by frame, and the IDs of the objects will be sorted in ascending order.

## Availability of source code and requirements

The requirements include the following:

- Project name: ANTS_marking_and_analysis_tools
- Project homepage: https://github.com/holmescao/ANTS_marking_and_analysis_tools
- Operating system(s): Platform independent
- Programming language: Python, MATLAB, Shell
- Other requirements: MATLAB R2021b (with Image Processing Toolbox)
- License: MIT License
- RRID: SCR_022543
- biotoolsID identifiers: ants_marking_and_analysis_tools

## Abbreviations

CV: computer vision: FPS: frames per second; ID: identity.

## Competing Interests

The authors declare that they have no competing interests.

## Funding

This work was supported by the Natural Science Foundation of Fujian Province (No. 2019J01002) and the National Nature Science Foundation of China (No. 32071057, No. 61673322, and No. 31200769) and was partly supported by the Key Project of National Key R&D project (No. 2017YFC1703303).

## Authors’ Contributions

M.W. and S.G conceived the experiment(s), X.C. conducted the experiment(s), and X.C. analyzed the results. All authors reviewed the manuscript.

## Data Availability

The dataset supporting the results of this article is published in the ANTS–ant detection and tracking repository [42] and the *GigaScience* database GigaDB [43]. The files associated with this dataset are licensed under a CC0 waiver, dedicating them to the public domain.

## Supplementary Material

giac096_GIGA-D-22-00055_Original_Submission

giac096_GIGA-D-22-00055_Revision_1

giac096_GIGA-D-22-00055_Revision_2

giac096_GIGA-D-22-00055_Revision_3

giac096_Response_to_Reviewer_Comments_Original_Submission

giac096_Response_to_Reviewer_Comments_Revision_1

giac096_Response_to_Reviewer_Comments_Revision_2

giac096_Reviewer_1_Report_Original_Submission Reuber Antoniazzi -- 4/8/2022 Reviewed

giac096_Reviewer_2_Report_Original_Submission HARUNA Fujioka -- 4/20/2022 Reviewed

giac096_Reviewer_3_Report_Original_Submission Leandro Bugnon -- 4/25/2022 Reviewed
