# Supplementary material for: A dataset of ant colonies’ motion trajectories in indoor and outdoor scenes to study clustering behavior
Source: Gigascience. 2022 Oct 28;11:giac096. doi: 10.1093/gigascience/giac096 (PMC9614923; doi:10.1093/gigascience/giac096)
Supplement: giac096_GIGA-D-22-00055_Original_Submission [file giac096_giga-d-22-00055_original_submission.pdf]

GigaScience

A dataset of ant colonies motion trajectories in indoor and outdoor scenes for social cluster behavior study

--Manuscript Draft--

**Manuscript Number:**	GIGA-D-22-00055
**Full Title:**	A dataset of ant colonies motion trajectories in indoor and outdoor scenes for social cluster behavior study
**Article Type:**	Research
**Funding Information:**	Natural Science Foundation of Fujian Province (2019J01002) — Dr Meihong Wu
**Abstract:**	Motion and interaction of social insects (such as ants) have been studied by many researchers to understand the clusteringmechanism. Most studies in the field of ant behavior have only focused on indoor environments, while outdoor environments arestill underexplored. In this paper, we collect 10 videos of ant colonies from different indoor and outdoor scenes. And we develop animage sequence marking software named VisualMarkData, which enables us to provide annotations of ants in the video. In all 5354 frames, the location information and the identification number of each ant are recorded for a total of 712 ants and 114112 annotations. Moreover, we provide visual analysis tools to assess and validate the technical quality and reproducibility of our data.It is hoped that this dataset will contribute to a deeper exploration on the behavior of the ant colony.
**Corresponding Author:**	Xiaoyan Cao, M.D.<br>Xiamen University<br>Xiamen, CHINA
**Corresponding Author Secondary Information:**	
**Corresponding Author's Institution:**	Xiamen University
**Corresponding Author's Secondary Institution:**	
**First Author:**	Meihong Wu
**First Author Secondary Information:**	
**Order of Authors:**	Meihong Wu
	Xiaoyan Cao, M.D.
	Xiaoyu Cao
	Shihui Guo
**Order of Authors Secondary Information:**	
**Additional Information:**	
**Question**	**Response**
Are you submitting this manuscript to a special series or article collection?	No
**Experimental design and statistics**<br><br>Full details of the experimental design and statistical methods used should be given in the Methods section, as detailed in our Minimum Standards Reporting Checklist.	Yes

Information essential to interpreting the data presented should be made available in the figure legends.<br><br>Have you included all the information requested in your manuscript?	
**Resources**<br><br>A description of all resources used, including antibodies, cell lines, animals and software tools, with enough information to allow them to be uniquely identified, should be included in the Methods section. Authors are strongly encouraged to cite Research Resource Identifiers (RRIDs) for antibodies, model organisms and tools, where possible.<br><br>Have you included the information requested as detailed in our Minimum Standards Reporting Checklist?	Yes
**Availability of data and materials**<br><br>All datasets and code on which the conclusions of the paper rely must be either included in your submission or deposited in publicly available repositories (where available and ethically appropriate), referencing such data using a unique identifier in the references and in the “Availability of Data and Materials” section of your manuscript.<br><br>Have you have met the above requirement as detailed in our Minimum Standards Reporting Checklist?	Yes

Manuscript
Click here to access/download;Manuscript;ant_dataset_GigaScience.pdf

OXFORD (GIGA)^n SCIENCE

GigaScience, 2017, 1–7

doi: xx.xxxx/xxxx
Manuscript in Preparation
Paper

PAPER

A dataset of ant colonies motion trajectories in indoor and outdoor scenes for social cluster behavior study

Meihong Wu[1,†], Xiaoyan Cao[1,†], Xiaoyu Cao[2] and Shihui Guo[1,*]

[1]School of Informatics, Xiamen University, Xiamen, 361000, China and [2]Chemistry and Chemical Engineering, Xiamen University, Xiamen, 361000, China

[*]guoshihui@xmu.edu.cn
[†]Contributed equally.

Abstract

Motion and interaction of social insects (such as ants) have been studied by many researchers to understand the clustering mechanism. Most studies in the field of ant behavior have only focused on indoor environments, while outdoor environments are still underexplored. In this paper, we collect 10 videos of ant colonies from different indoor and outdoor scenes. And we develop an image sequence marking software named VisualMarkData, which enables us to provide annotations of ants in the video. In all 5354 frames, the location information and the identification number of each ant are recorded for a total of 712 ants and 114112 annotations. Moreover, we provide visual analysis tools to assess and validate the technical quality and reproducibility of our data. It is hoped that this dataset will contribute to a deeper exploration on the behavior of the ant colony.

Key words: social insects; outdoor scenes; image sequence marking software

Background

Social insects often tend to cluster into a colony [1], which forms a complex social network [2]. From time to time, the social network springs up with self-organized clustering behaviors, including division of labor [3], task specialization [4], and distributed problem solving [5]. Biologists analyze the evolution of social network to understand the clustering behavior of insects [6], thus promoting the development of relevant modern applications, such as wireless communication [7] and cluster intelligent control [8]. The key requirement of this research is the ability to track the motions and interactions of each individual robustly and accurately.

Until the late 20th century, biologists still manually marked the motion trajectories on the video to guarantee the quality. However, they have to track each individual at one time, which might mean watching the entire video 50 times or more in a crowded scene. [9] Obviously, manually tracking is time-consuming and prone to human error. It becomes an inhibiting factor in obtaining the complete and accurate dataset required to analyze the evolution of social networks. Therefore, in the past two decades, attempts have been made to automate the tracking process for social insects utilizing computer vision (CV) techniques [10, 11, 12, 13, 14].

Traditional CV techniques free researchers from manual work through approaches such as foreground segmentation algorithm [15], temporal difference method [10] and hungarian algorithm [16]. Such approaches, however, have failed to address the noise in the image [17], resulting in the limitation that a laboratory environment with a clean background is needed. Nevertheless, many scientifically valuable results are obtained in nature rather than laboratory environment [18, 19, 20, 21].

Fortunately, with the emergence of deep learning, CV techniques are already capable of addressing many complex tasks [22, 23, 24], which brings a piece of good news to automated insect tracking in outdoor scenes. Several studies have explored automated multi-ant tracking in outdoor scenes using deep learning-based models [25, 26]. Experimental results demonstrate that these models could be scaled up into a cost-effective alternative to traditional manual tracking methods which are typically costly and/or labor intensive [25, 26]. A critical requirement for the development of these models is access to the datasets containing motion trajectories of insects in the video. To the best of our knowledge, however, the current studies are all using only one outdoor-scene dataset, which is lack of data diversity.

Compiled on: March 17, 2022.
Draft manuscript prepared by the author.

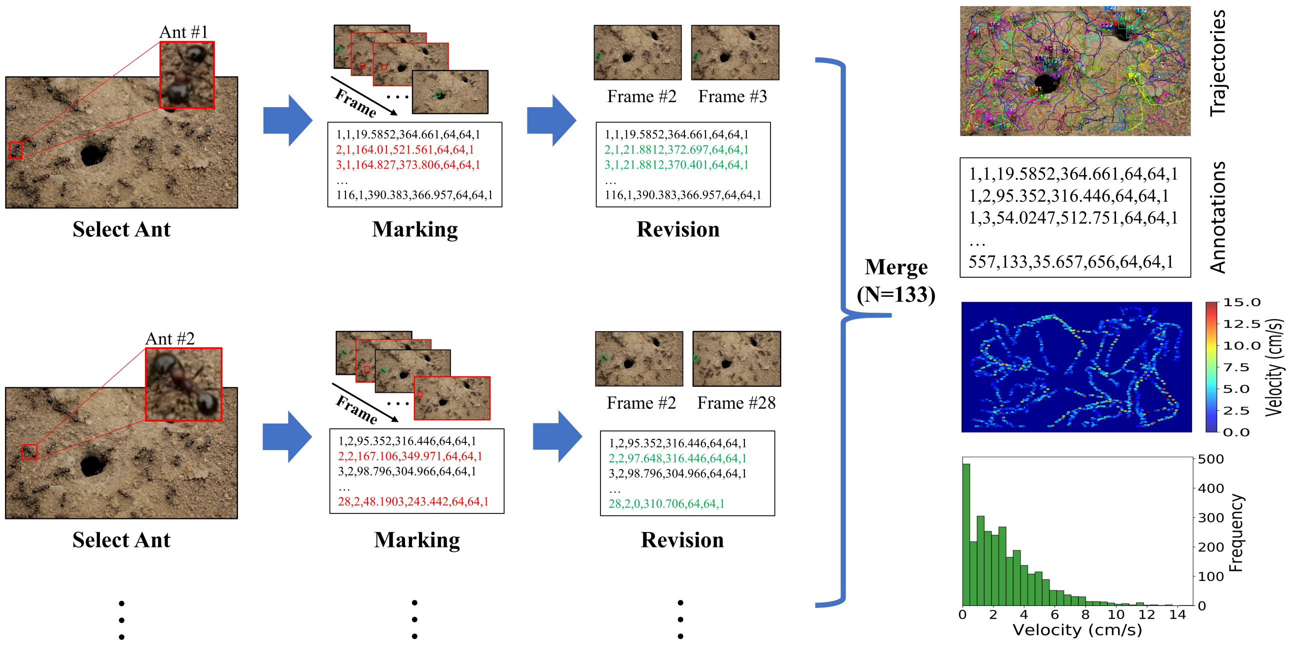

Figure 1. The pipeline for marking motion trajectories of ants in an image sequence, taking an outdoor scene as an example. A total of 133 ants appear in this image sequence, and we select one ant to be marked in each epoch. We use a square bounding box to point out the ant's location and record the relevant parameters at the same time. After all ants of the entire image sequence have been marked, we check the quality of the annotations frame by frame so that wrong annotations (red font) can be corrected (green font). Then, we merge all the annotations of the image sequence into one file. Additionally, we provide three visualization tools to verify the data quality.

Sequence	FPS	Resolution	Length	Ants	Annotations
Seq0001	25	1920×1080	351 (00:14)	10	3510
Seq0002			351 (00:14)	10	3510
Seq0003			351 (00:14)	10	3510
Seq0004			351 (00:14)	10	3510
Seq0005			1001 (00:40)	10	3510
Seq0006	30	1280×720	600 (00:20)	73	11178
Seq0007			677 (00:23)	162	25158
Seq0008			577 (00:19)	133	10280
Seq0009			526 (00:18)	193	27902
Seq0010			569 (00:19)	101	22044

Table 1. Statistics of ant videos with annotations in indoor and outdoor scenes.

In this paper, we collect video recordings of 10 ant colonies from different scenes (including indoor and outdoor scenes). Besides, we develop an image sequence mark software named VisualMarkData, which is used to mark the pixel patches covered by ants in each frame of the video. The total size of our dataset is 5354 frames, 712 ants, and 114112 labels. We believe that our dataset will benefit future research on social insect behavior analysis.

Data Description

We collect 10 videos that record activities of different ant colonies, including colonies from both indoor and outdoor scenes. To help us mark the motion trajectories, we develop an image sequence marking software called VisualMarkData.

After spending a large quantity of time and effort, we obtain a dataset with 5354 frames and 114112 annotations. Table 1 shows the statistical information of our dataset.

Data acquisition

Indoor environment

We collect 50 workers of Japanese arched ants species, which ranged from 7.4 to 13.8 *mm* in body length [27]. We construct a laboratory environment for them, including a stable light source and a transparent plastic container. We randomly divide them into 5 colonies of 10 ants each. Then, we load each colony into the container in turns and film their activities with a high-resolution video camera.

Outdoor environment

We collect 5 videos of black ant colonies in different outdoor environments, with the number of black ants ranging from 73 to 193 in the videos. The body length of black ants is between 8 to 10 *mm* [28]. These videos are provided by Depositphotos, an online video site (`https://cn.depositphotos.com/home.html`), and access is subject to a royalty-free license.

Data Records

The dataset consists of 10 image sequences from different scenes in JPEG digital image format, which is publicly available on `https://data.mendeley.com/datasets/9ws98g4npw/3`. Alongside, we provide annotations marked by VisualMarkData for all image sequences in the form of text. In the dataset, the images and annotations of each sequence are organized into three folders are named 'det', 'gt', and 'img'.

Position	Name	Description
1	Frame number	Indicate at which frame the object is present
2	Identity number	Each ant trajectory is identified by a unique ID (-1 for detections)
3	Bounding box left	Coordinate of the top-left corner of the ant bounding box
4	Bounding box top	Coordinate of the top-left corner of the ant bounding box
5	Bounding box width	Width in pixels of the ant bounding box
6	Bounding box height	Height in pixels of the ant bounding box
7	Confidence score	Indicates how confident the detector is that this instance is a ant. For the ground truth and results, it acts as a flag whether the entry is to be considered

Table 2. Data format for annotation files, both for 'det.txt' and 'gt.txt' files.

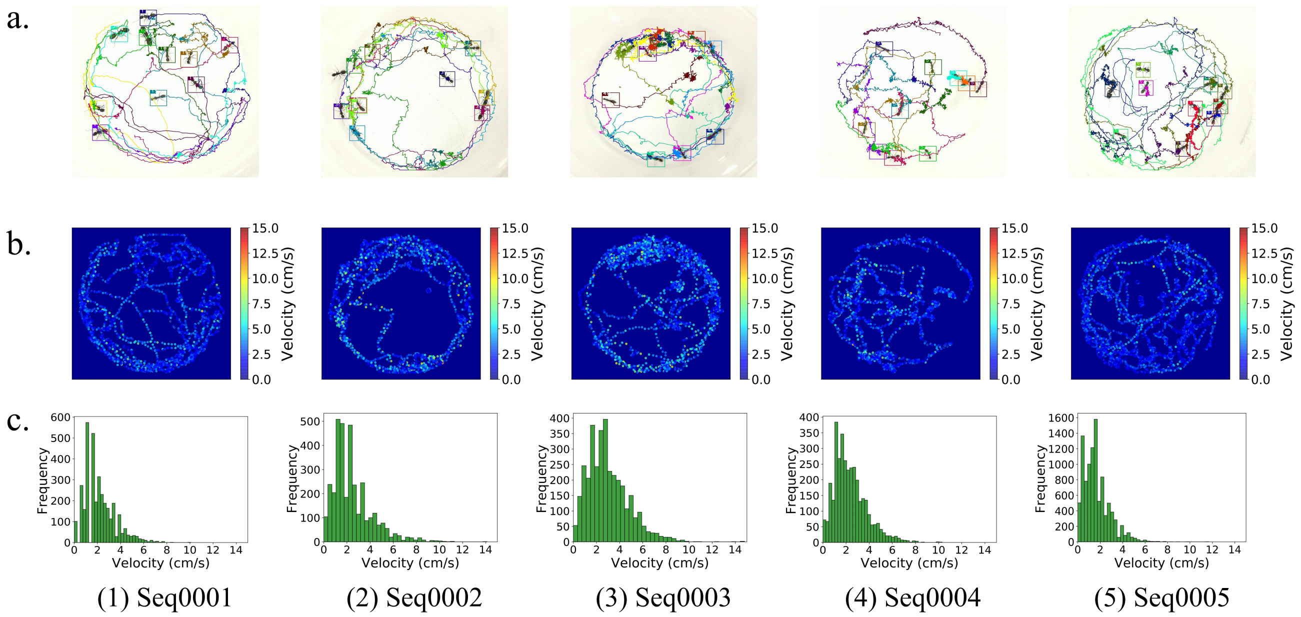

Figure 2. Visual analysis of the marking results on indoor ant videos. (a) Visualization of motion trajectories of the ants for each sequence of the indoor scene. (b) Speed distributions in image space for 5 sequences of indoor scenes, respectively. (c) Ant speed histograms per indoor sequence.

Det folder

The 'det' folder contains a 'det.txt' file which is the ground truth for detection, recording the location parameters of the ants in all frames, which is similar to multi-object tracking challenge [29]. Each line represents one ant instance, and it contains 7 values as shown in Table 2. The first number indicates in which frame the ant appears, while the second number identifies that ant as belonging to a trajectory by assigning a unique ID (set to -1 in a detection file, as no ID is assigned yet). The next four numbers indicate the location of the bounding box of the ant in 2D image coordinates. The location is indicated by the top-left corner as well as the width and height of the bounding box. This is followed by a single number, which denotes the confidence score.

Gt folder

The 'gt' folder contains a 'gt.txt' file, which is the ground truth for muti-object tracking. Similar to the 'det.txt' file, it also contains 7 values (see details in Table 2). The difference compared to the 'det.txt' file is that the second number in the 'gt.txt' file records the ID of an ant as belonging to a trajectory, which provides the key information for implementing multi-ant tracking. Besides, each ant can be assigned to only one trajectory.

Img folder

The 'img' folder stores the original image sequence converted from the video. All images are converted to JPEG and named sequentially to a 6-digit file name (e.g. 000001.jpg).

Analyses

Visually confirm

The ground truth annotations for all image sequences in the dataset are visually confirmed by the data annotation staff. The visual reviewing consists of two aspects, sequence-level (coarse-grained) and image-level (fine-grained).

Firstly, staffs perform a coarse-grained review of a single sequence. Specifically, we draw the annotations on the corresponding images, and then convert the image sequence to video. By replaying the video, staff can quickly confirm which segments of the video are poor quality and needed to be re-marked. For each scene, an example image frame is shown in Figure 2 (a) and Figure 3 (a). After that, staff reviews the quality of annotations frame-by-frame via VisualMarkData. For inaccurate annotations, staff modifies manually by using the "Check and modify" function of VisualMarkData (see details in Methods).

Motion speed analysis

Further, to demonstrate the reliability of our dataset, we analyze the distribution of the movement speed of the ants in our dataset. First, for each ant, we use the 2D Euclidean distance [30] to calculate its pixel distance between two adjacent frames. Therefore, the pixel distance $\triangle ps_t$ of the ant at frame t can be defined by the following equation:

$$\triangle ps_t = \sqrt{(px_t - px_{t-1})^2 + (py_t - py_{t-1})^2} \quad (1)$$

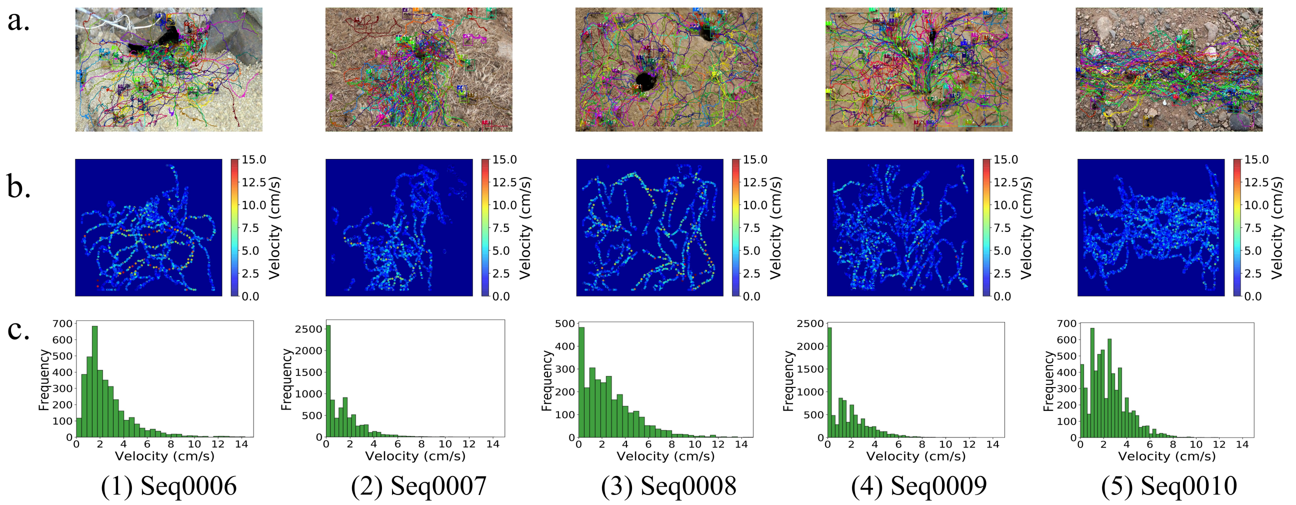

Figure 3. Visual analysis of the marking results on outdoor ant videos. (a) Visualization of motion trajectories of the ants for each sequence of the outdoor scene. (b) Speed distributions in image space for 5 sequences of outdoor scenes, respectively. (c) Ant speed histograms per outdoor sequence.

where px_t denotes the pixel position of the ant in the horizontal direction at frame t. Similarly, py_t denotes the pixel position in the vertical direction. To convert the pixel distance to real-world coordinates, we divide the ant's body length L (unit: m) in the real world by body length n (unit: *pixel*) in the image. Thus, the real-world displacement of the ant at frame t, $\triangle s_t$ (unit: m) can be expressed as follows:

$$\triangle s_t = \triangle ps_t \times L/n \tag{2}$$

Since the FPS for a specifc video is a constant f_c, the velocity v_t (unit: $m \cdot s^{-1}$) at frame t can be formulated as:

$$v_t = \frac{\triangle s_t}{1/f_c} \tag{3}$$

Where, the v_0 is set to 0.

According to the aforementioned equations, combined with the location information of ants in annotations, we can analyze the motion speed of ants in the video, as shown in Figure 2 (b), (c) and Figure 3 (b), (c). Specifically, the overall motion speed of ants in indoor and outdoor scenes are 2.16±1.49 $cm \cdot s^{-1}$ and 1.98±1.84 $cm \cdot s^{-1}$, respectively. These values are within a reasonable range (ants average motion speed is 2.85 $cm \cdot s^{-1}$ under bi-directional traffic condition [31]). This demonstrates that the ant colony activity dataset we collected and marked is real and reliable.

Discussion

The image sequence marking software VisualMarkData is a toolkit with interactive visualization. The goal of the software is to provide a convenient tool for researchers marking movement trajectories of social insects in videos, thus facilitating the study of the behavioral mechanisms of social insects. Additionally, by using the software, researchers will obtain standardized annotation data, as details in the previous section. VisualMarkData is open source, which enables researchers to mark their image sequence datasets of any multi-object motion scenario. Alongside, we have provided publicly available Python Scripts to illustrate the analysis of data as well as usage of the data. To visualize and reproduce the results described in the Technical Validation section, we develop two scripts for the researchers. Also, we provide another script to calculate metrics [29] of multi-object tracking that enables any deep learning algorithm to evaluate the tracking accuracy on our dataset. The annotated trajectory data can be used for training and testing of supervised learning models, thus providing a powerful tool for studying a wider range of ant colony behaviors.

In the future, we will enrich the VisualMarkData with more features to reduce the difficulty of marking and improve the efficiency of marking. The software currently marks targets based on their center points, and we are considering introducing stretchable annotation capabilities based on rectangles or ellipses. In addition, the simultaneous annotation of multiple targets in one frame is also a feature worth developing. Along with that, we can introduce semi-automated annotation, i.e., embedding a neural network model into the VisualMarkData, which will automatically predict and annotate objects of the current frame based on the information in the previous frame. Thus, the annotators only need to fine-tune the annotation, which will significantly improve the efficiency of the annotation.

The dataset and VisualMarkData will boost researchers both in biology and computer science to study on behavior of social insects in different environments. We hope that this work will contribute to the potential discovery of ant colony behavioral mechanisms and facilitate the application of the image processing field in biology.

Potential implications

Swarm behavior is one of the most important features of social insects, which has important significance for the study of embodied intelligence [8]. Specifically, social insects often tend to cluster into a colony [1], which forms a complex dynamical system together with the surrounding environment [2]. So far, researchers do not know enough about the mechanisms behind swarm behaviors of social insects. We believe our image sequence marking software and dataset could facilitate the analysis of ant colony behavior leading to the development of embodied intelligence.

Methods

Hardware devices for acquiring raw data

For indoor environments, we use a cylindrical container made of transparent plastic providing a space for the ants to move around. This container has a bottom diameter of 10 cm, a side height of 15 cm, and is not closed at the top. Ants, loaded in the container, are filmed with a high-resolution video camera (Panasonic GX 85) with 25 frames per second (FPS) in the format H.264 with a resolution of 1920×1080 pixels. And the distance between the camera and

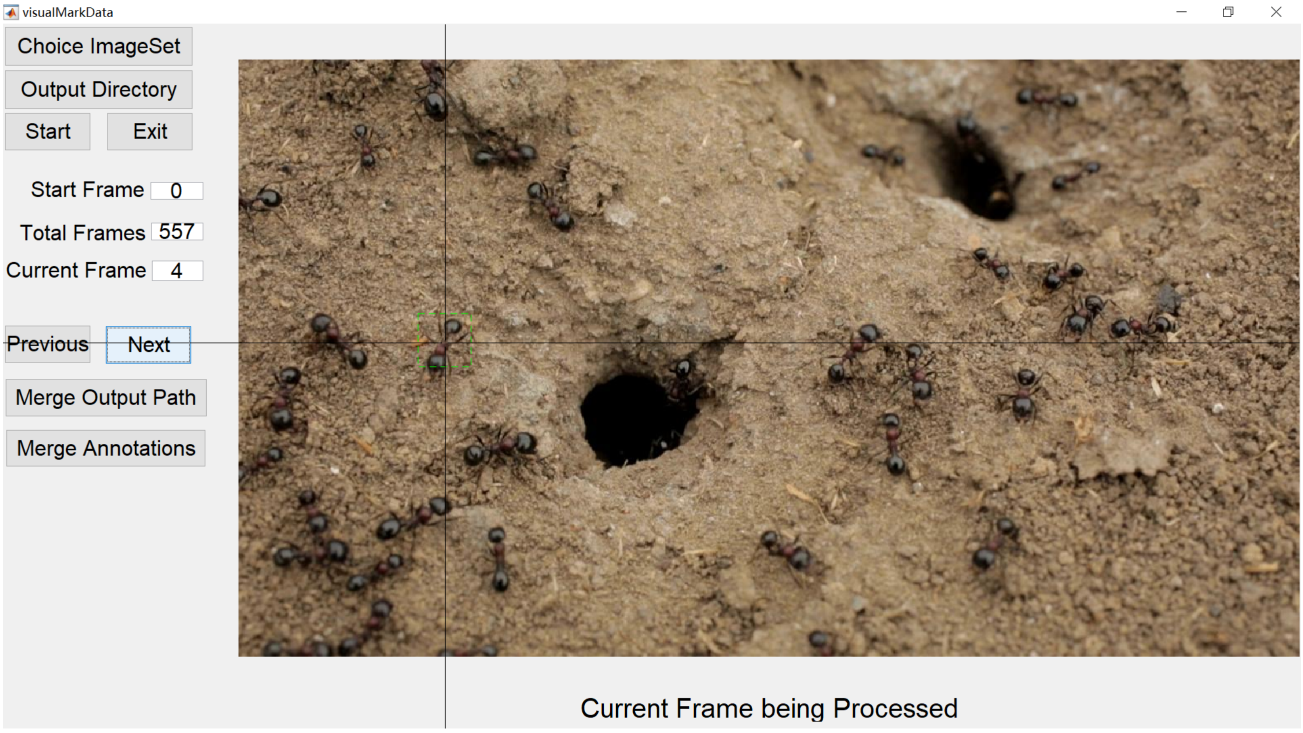

Figure 4. The interactive interface of our image sequence marking software is named VisualMarkData. After selecting an image sequence, the user can acquire the annotation by clicking on the center location of the ant's body.

the top of the container is 30 *cm* so that the filming view of the camera can cover the whole container. To ensure stable filming, we fix the camera on a tripod, as well as hanging a light bulb above the container. Besides, the anti-dusting powder is applied to the inner wall of the container, preventing ants from escaping from the container during the filming.

Description of the marking software VisualMarkData

We develop an image sequence marking software called VisualMarkData to provide the locations and identification numbers of objects in the sequence for motion analysis. The operation procedure of VisualMarkData is as follows, and its interface is shown in Figure 4.

- **Choose Image Set.** Before marking, the user should click "Choose ImageSet" to select an image set. The filename of the image set is defined in the format of "SeqXObjectYImageZ", where X is the name of the sequence, Y is the number of objects in the first frame and Z is the size of the bounding box which represents the object. For example, the image set, named "Seq0001Object10Image94", indicates that the sequence "0001" contains 10 objects in the first frame, and each object will be marked with a bounding box with the size of 94x94.
- **Create Output Directory.** The user needs to click "Output Directory" to select the storage path of annotations. Since VisualMarkData only focuses on one object per marking round (each round goes through the whole image sequence), the output folder is suggested to be named with the identification number of the object, e.g. "0001". As the identity number of the object is user-defined, the user can use any number for the object and folder as long as it is unique.
- **Select Start Frame.** In the last step before starting marking, you need to enter the start frame, the default value is 0. This means that you are allowed to exit the software halfway and continue the progress of the current marking task the next time. Then, you can click the "Start" button.
- **Marking.** The user clicks on the center of the object in the current frame, and the software will automatically save the digital location of the center, as well as a bounding box centered on the object. It should be emphasized that the user only marks the same object until finishes the entire image sequence, and then the user can focus on another object by repeating the same operation for the previous one.
- **Next Frame.** The user clicks the "Next" button to show the next frame on the window of the software. The marked location on the previous frame will be displayed with a green-dotted, which can help the user quickly locate the target object.
- **Previous Frame.** If the marked location of the previous frame is incorrect, the user can click the "Previous" button to roll back one frame.
- **Check and Modify.** After the user finishes marking the entire image set, checking is needed to guarantee the quality. In this case, the user can enter the specific frame to modify the annotations by carrying out **Select Start Frame** step.
- **Merge Annotations.** After all objects in a sequence have been marked and reviewed, the user needs to click the "Merge" button, thereby all annotations for each object will be sorted by frames and then the ID of the object both in ascending order.

Availability of source code and requirements

Lists the following:

- Project name: ANTS_marking_and_analysis_tools
- Project home page: e.g. `https://github.com/holmescao/ANTS_marking_and_analysis_tools`
- Operating system(s): Platform independent
- Programming language: Python, MATLAB, Shell
- Other requirements: MATLAB R2021b (with Image Processing Toolbox)
- License: MIT License

Availability of supporting data and materials

The dataset supporting the results of this article is available in the ANTS–ant detection and tracking repository at http://dx.doi.org/10.17632/9ws98g4npw.4. Note that the files associated with this dataset are licensed under a Public Domain Dedication license.

Declarations

List of abbreviations

CV: computer vision; ID: identity; FPS: frames per second

Consent for publication

Not applicable

Competing Interests

The authors declare that they have no competing interests.

Funding

This work was supported by the Natural Science Foundation of Fujian Province of China (No. 2019J01002).

Author's Contributions

M.W. and S.G conceived the experiment(s), X.C. conducted the experiment(s), X.C. analyzed the results. All authors reviewed the manuscript.

Acknowledgements

The authors thank the reviewers, for providing useful suggestions for improvements and valuable feedback on the workflow and the manuscript.

References

1. Vandermeer J, Perfecto I, Philpott SM. Clusters of ant colonies and robust criticality in a tropical agroecosystem. Nature 2008;451(7177):457–459.
2. Balch T, Khan Z, Veloso M. Automatically tracking and analyzing the behavior of live insect colonies. In: Proceedings of the fifth international conference on Autonomous agents; 2001. p. 521–528.
3. Hölldobler B, Wilson EO, et al. The ants. Harvard University Press; 1990.
4. Whitehouse ME, Jaffe K. Ant wars: combat strategies, territory and nest defence in the leaf-cutting antAtta laevigata. Animal Behaviour 1996;51(6):1207–1217.
5. Vaughan RT, Støy K, Sukhatme GS, Matarić MJ. Whistling in the dark: cooperative trail following in uncertain localization space. In: Proceedings of the fourth international conference on Autonomous agents; 2000. p. 187–194.
6. Fewell JH. Social insect networks. Science 2003;301(5641):1867–1870.
7. Motani M, Srinivasan V, Nuggehalli PS. Peoplenet: engineering a wireless virtual social network. In: Proceedings of the 11th annual international conference on Mobile computing and networking; 2005. p. 243–257.
8. Tiacharoen S, Chatchanayuenyong T. Design and development of an intelligent control by using bee colony optimization technique. American Journal of Applied Sciences 2012;9(9):1464.
9. Poff C, Nguyen H, Kang T, Shin MC. Efficient tracking of ants in long video with GPU and interaction. In: 2012 IEEE Workshop on the Applications of Computer Vision (WACV) IEEE; 2012. p. 57–62.
10. Khan Z, Balch T, Dellaert F. MCMC-based particle filtering for tracking a variable number of interacting targets. IEEE transactions on pattern analysis and machine intelligence 2005;27(11):1805–1819.
11. Khan Z, Balch T, Dellaert F. MCMC data association and sparse factorization updating for real time multitarget tracking with merged and multiple measurements. IEEE transactions on pattern analysis and machine intelligence 2006;28(12):1960–1972.
12. Oh SM, Rehg JM, Dellaert F. Parameterized duration mmodeling for switching linear dynamic systems. In: 2006 IEEE Computer Society Conference on Computer Vision and Pattern Recognition (CVPR'06), vol. 2 IEEE; 2006. p. 1694–1700.
13. Veeraraghavan A, Chellappa R, Srinivasan M. Shape-and-behavior encoded tracking of bee dances. IEEE transactions on pattern analysis and machine intelligence 2008;30(3):463–476.
14. Fletcher M, Dornhaus A, Shin MC. Multiple ant tracking with global foreground maximization and variable target proposal distribution. In: 2011 IEEE Workshop on Applications of Computer Vision (WACV) IEEE; 2011. p. 570–576.
15. Li M, Zhang Z, Huang K, Tan T. Estimating the number of people in crowded scenes by mid based foreground segmentation and head-shoulder detection. In: 2008 19th international conference on pattern recognition IEEE; 2008. p. 1–4.
16. Li Y, Huang C, Nevatia R. Learning to associate: Hybridboosted multi-target tracker for crowded scene. In: 2009 IEEE conference on computer vision and pattern recognition IEEE; 2009. p. 2953–2960.
17. Zhao M, Liu H, Wan Y. An improved Canny edge detection algorithm based on DCT. In: 2015 IEEE International Conference on Progress in Informatics and Computing (PIC) IEEE; 2015. p. 234–237.
18. Schmelzer E, Kastberger G. 'Special agents' trigger social waves in giant honeybees (Apis dorsata). Naturwissenschaften 2009;96(12):1431–1441.
19. Kastberger G, Weihmann F, Hoetzl T. Social waves in giant honeybees (Apis dorsata) elicit nest vibrations. Naturwissenschaften 2013;100(7):595–609.
20. Tan K, Dong S, Li X, Liu X, Wang C, Li J, et al. Honey bee inhibitory signaling is tuned to threat severity and can act as a colony alarm signal. PLoS biology 2016;14(3):e1002423.
21. Dong S, Wen P, Zhang Q, Wang Y, Cheng Y, Tan K, et al. Olfactory eavesdropping of predator alarm pheromone by sympatric but not allopatric prey. Animal Behaviour 2018;141:115–125.
22. Schor N, Bechar A, Ignat T, Dombrovsky A, Elad Y, Berman S. Robotic disease detection in greenhouses: Combined detection of powdery mildew and tomato spotted wilt virus. IEEE Robotics and Automation Letters 2016;1(1):354–360.
23. Wang G, Li W, Zuluaga MA, Pratt R, Patel PA, Aertsen M, et al. Interactive medical image segmentation using deep learning with image-specific fine tuning. IEEE transactions on medical imaging 2018;37(7):1562–1573.
24. Wang C. Research and application of traffic sign detection and recognition based on deep learning. In: 2018 International Conference on Robots & Intelligent System (ICRIS) IEEE; 2018. p. 150–152.
25. Imirzian N, Zhang Y, Kurze C, Loreto RG, Chen DZ, Hughes DP. Automated tracking and analysis of ant trajectories shows variation in forager exploration. Scientific reports 2019;9(1):1–10.
26. Cao X, Guo S, Lin J, Zhang W, Liao M. Online tracking of ants

based on deep association metrics: method, dataset and evaluation. Pattern Recognition 2020;103:107233.

27. Terayama M, Ogata K. Two new species of the ant genus Probolomyrmex (Hymenoptera, Formicidae) from Japan. Kontyu 1988;56(3):590–594.
28. Ayieko MA, Kinyuru J, Ndong'a M, Kenji G. Nutritional value and consumption of black ants (Carebara vidua Smith) from the Lake Victoria region in Kenya. Advance Journal of Food Science and Technology 2012;.
29. Leal-Taixé L, Milan A, Reid I, Roth S, Schindler K. Motchallenge 2015: Towards a benchmark for multi-target tracking. arXiv preprint arXiv:150401942 2015;.
30. Fabbri R, Costa LDF, Torelli JC, Bruno OM. 2D Euclidean distance transform algorithms: A comparative survey. ACM Computing Surveys (CSUR) 2008;40(1):1–44.
31. Wang Q, Song W, Zhang J, Lo S. Bi-directional movement characteristics of Camponotus japonicus ants during nest relocation. Journal of Experimental Biology 2018;221(18):jeb181669.
