# Supplementary material for: A dataset of ant colonies’ motion trajectories in indoor and outdoor scenes to study clustering behavior
Source: Gigascience. 2022 Oct 28;11:giac096. doi: 10.1093/gigascience/giac096 (PMC9614923; doi:10.1093/gigascience/giac096)
Supplement: giac096_GIGA-D-22-00055_Revision_1 [file giac096_giga-d-22-00055_revision_1.pdf]

GigaScience

A dataset of ant colonies motion trajectories in indoor and outdoor scenes for social cluster behavior study

--Manuscript Draft--

**Manuscript Number:**	GIGA-D-22-00055R1
**Full Title:**	A dataset of ant colonies motion trajectories in indoor and outdoor scenes for social cluster behavior study
**Article Type:**	Data Note
**Funding Information:**	Natural Science Foundation of Fujian Province (2019J01002) — Dr Meihong Wu<br>National Nature Science Foundation of China (32071057) — Dr Meihong Wu<br>National Nature Science Foundation of China (61673322) — Dr Meihong Wu<br>National Nature Science Foundation of China (31200769) — Dr Meihong Wu
**Abstract:**	Motion and interaction of social insects (such as ants) have been studied by many researchers to understand the clustering mechanism. Most studies in the field of ant behavior have only focused on indoor environments (laboratory setup), while outdoor environments (natural environments) are still underexplored.<br>In this paper, for the first time, we collect 10 videos and 3 species of ant colonies from different scenes, including 5 indoor and 5 outdoor scenes.<br>And we develop an image sequence marking software named VisualMarkData, which enables us to provide annotations of ants in the video:<br>(1) offers a comprehensive annotation of states at individual-target as well as colony-target level;<br>(2) provides a simple matrix format to represent multiple targets and multiple groups of annotations (along with their IDs and behavior labels);<br>(3) during the annotation process, we propose a simple and effective visualization that takes the annotation information of the previous frame as a reference and then simply clicks on the center point of each target to complete the annotation;<br>and (4) we develop a user-friendly windows-based GUI to minimize labor and maximize annotation quality.<br>In all 5,354 frames, the location information and the identification number of each ant are recorded for a total of 712 ants and 114,112 annotations. Moreover, we provide visual analysis tools to assess and validate the technical quality and reproducibility of our data. It is hoped that this dataset will contribute to a deeper exploration on the behavior of the ant colony.
**Corresponding Author:**	Xiaoyan Cao, M.D.<br>Xiamen University<br>Xiamen, CHINA
**Corresponding Author Secondary Information:**	
**Corresponding Author's Institution:**	Xiamen University
**Corresponding Author's Secondary Institution:**	
**First Author:**	Meihong Wu
**First Author Secondary Information:**	
**Order of Authors:**	Meihong Wu<br>Xiaoyan Cao, M.D.

Powered by Editorial Manager® and ProduXion Manager® from Aries Systems Corporation

	Ming Yang
	Xiaoyu Cao
	Shihui Guo
**Order of Authors Secondary Information:**	
**Response to Reviewers:**	We want to thank you for your valuable comments.<br>We submitted 3 files, including a revised manuscript, a track changes file (to highlight differences between the revised and the original manuscript), and this file, i.e., the response letter (a complete response to the editor and all reviewers). In the following, we respond to each of your concerns and recommendations.<br>(EC: Editor’s Comment, RC: Reviewer's Comment, AR: Authors' Response)<br><br>Editor<br>EC0: In particular, the reviewers mention the need to add more specific information and metadata to make the data set useful for others (including, for example, the scientific name of the ant species used and more exact details on data acquisition - see the reports below). In addition to the reviewers' reports, some minor editorial points from my side:<br>AR0: We thank the editor and reviewers for their kind comments and responded in a point-by-point manner.<br><br>EC1: We will consider the submission for publication as a "Data Note" - please check our instructions or authors for formatting guidelines for this article type (you do not need to change the text in a major way, but please try to use the section headings as indicated for the "Data Note" article type, if applicable). (Please also note that the Data Note article type has a lower Article Processing Charge than "Research".)<br>AR1: We modified the article to "Data Note" type by referring to the formatting guidelines on the journal website.<br><br>EC2: On page 2, you mention that data is available "subject to a royalty-free license", but at the end you provide another source which is public domain /cc0 (which is great). If the data is available under public domain, I recommend you remove the alternative source, or clarify if the data fulfills public domain criteria - (or are these different data sets? If you need help to make data available, we can also store it under a cc0 licence on our server GigaDB.)<br>AR2: We removed the alternative sources mentioned in the second page in the revised manuscript.<br><br>EC3: In the methods section, I recommend the use of past tense for the procedures that you carried out. ("We collected ants" instead of "we collect ants", etc..). Please also include the helpful wording suggestions from the reviewers' reports.<br>AR3: In the Data Description section and the Methods section of the revised manuscript, we have changed the procedures that are carried out to the past tense (see details in lines 96-124 and 274-303 of the revised manuscript).<br><br>EC4: In addition, please register the new software application in the bio.tools and SciCrunch.org databases to receive RRID (Research Resource Identification Initiative ID) and biotoolsID identifiers, and include these in your manuscript (in the code availability section). This will facilitate tracking, reproducibility and re-use of your tool.<br>AR4: We registered in bio.tools and SciCrunch.org respectively. The biotoolsID identifiers we obtained are ants_marking_and_analysis_tools and the RRID is SCR_022543. These information can be found in the Availability of source code and requirements section in lines 365-366 of the revised manuscript.<br><br>Reviewer reports:<br>Reviewer #1<br>RC1.0: Authors proposed a new software to evaluate ant movement, very useful to understand ant behavior and also to use their patterns to apply to other areas of science. I found a very interesting manuscript, well written and well addresses as well. This is a great advance in the methods to evaluate ant foraging, which I feel is useful for scientific researchers from many areas. I have some suggestions that I hope can help the authors strengthen the manuscript, making it clear to readers.<br>AR1.0: We thank the reviewer for the kind comments.

Powered by Editorial Manager® and ProduXion Manager® from Aries Systems Corporation

RC1.1: [Abstract] Lines 1,2: The clustering mechanism is most related to inside the colony than outside, in terms of the behavior of ant colonies that cluster their corpses and sort their larvae inside the colony. Or it could be valid for outside the colony like the authors made, at the entrance of the nest?
AR1.1: As you pointed out, clustering mechanism is most related to inside the colony, hence what we want to explore is the mechanism inside the colony. However, it seems that our statement is not clear enough. The so-called "indoor" and "outdoor" refer to the laboratory environment with a clean background (as shown in Figure 2a) and the natural environment (as shown in Figure 3a), respectively. We modified the abstract to eliminate ambiguity, as detailed in lines 2-3 of the revised manuscript, and shown below.
Origin: "Most studies in the field of ant behavior have only focused on indoor environments, while outdoor environments are still underexplored."
Revision: "Most studies in the field of ant behavior have only focused on indoor environments (laboratory setup), while outdoor environments (natural environments) are still underexplored."

RC1.2: [Abstract] Line 3: Include how many videos were taken indoors and outdoor.
AR1.2: We have 10 videos in total, 5 of which are indoor (laboratory setting) scene and 5 of which are outdoor (natural environment) scene. The comparison before and after modification is as follows (see details in lines 3-4 of the revised manuscript):
Origin: "In this paper, we collect 10 videos of ant colonies from different indoor and outdoor scenes."
Revision: "In this paper, for the first time, we collect 10 videos and 3 species of ant colonies from different scenes, including 5 indoor and 5 outdoor scenes."

RC1.3: [Abstract] Line 3: Maybe "And" instead of "In addition".
AR1.3: "In addition" has been changed to "And". The comparison before and after modification is as follows (see details in line 4 of the revised manuscript):
Origin: "In addition, we develop an image sequence marking software named VisualMarkData …"
Revision: "And we develop an image sequence marking software named VisualMarkData …"

RC1.4: [Abstract] Line 5: Maybe using the notation "114,112" instead of "114112".
AR1.4: "114112" has been changed to "114,112". The comparison before and after modification is as follows (see details in lines 10-11 of the revised manuscript):
Origin: "In all 5354 frames, the location information and the identification number of each ant are recorded for a total of 712 ants and 114112 annotations.”
Revision: "In all 5,354 frames, the location information and the identification number of each ant are recorded for a total of 712 ants and 114,112 annotations.”

RC1.5: [Introduction] Line 2 §2: The quality of what? From the information acquired?
AR1.5: To be more specific, we need to ensure the quality of the annotations, i.e., the accuracy. The comparison before and after modification is as follows (see details in lines 12-14 of the revised manuscript):
Origin: "Until the late 20th century, biologists still manually marked the motion trajectories on the video to guarantee the quality."
Revision: "Until the late 20th century, biologists still manually tracked the motion trajectories on the video to guarantee the accuracy of the marking."

RC1.6: [Introduction] Lines 5 §2: The final dot is after the reference: "[9].
AR1.6: The final dot has been placed after the reference. The comparison before and after modification is as follows (see details in line 16 of the revised manuscript):
Origin: ". [9]"
Revision: "[9]."

RC1.7: [Introduction] Lines 5,6 §2: I suggest removing the term "Obviously". Besides, the development of an automated tracking process is also time-consuming in relation to setting and calibrating the system (visual confirmation, etc.), right? And also, the automated tracking process can be more expensive than doing manually, right? If there is some previous work comparing the trade-off cost/benefit it could be included in the citation in order to better base the author's arguments.

AR1.7: "Obviously" has been removed. The comparison before and after modification is as follows (see details in line 16 of the revised manuscript):
Origin: "Obviously, manually tracking is time-consuming and prone to human error."
Revision: "Manually tracking is time-consuming and prone to human error."
Besides, the development of an automatic tracking process is indeed time-consuming. However, we only need to develop it once. It is reusable for same tasks in the future. Once development finished, additional manually making time for every video will no longer be needed.

RC1.8: [Introduction] Line 1 §3: "release" instead of "free"?
AR1.8: "Free" has been modified to "release". The comparison before and after modification is as follows (see details in line 22 of the revised manuscript):
Origin: "Traditional CV techniques free researchers …"
Revision: "Traditional CV techniques release researchers …"

RC1.9: [Introduction] Lines 12, 13 §4: Regarding "only one outdoor scene dataset": Which one?
AR1.9: We want to express that only a single outdoor scene sequence is used in all the current studies. References to these studies have been added now. The comparison before and after modification is as follows (see details in lines 42-44 of the revised manuscript):
Origin: "To the best of our knowledge, however, the current studies are all using only one outdoor-scene dataset, which is lack of data diversity."
Revision: "To the best of our knowledge, however, only a few works~\cite{imirzian2019automated, cao2020online} annotate motion trajectories in videos, and both use only a single outdoor scene sequence, which lacks data diversity."

RC1.10: [Introduction] §4: I would suggest using "5,354" and "114,112" instead of "5354" and "114112".
AR1.10: We totally agree with your suggestion. "5354" and "114112" have been modified to "5,354" and "114,112". The comparison before and after modification is as follows (see details in lines 82-83 of the revised manuscript):
Origin: "The total size of our dataset is 5354 frames, 712 ants, and 114112 labels."
Revision: "The total size of the dataset is 5,354 frames, 712 ants, and 114,112 labels."

RC1.11: [Data Description] Line 2 §2: What is the time interval of each frame taken from the video?
AR1.11: In Table 1 of the revised manuscript, column 3 indicates the FPS (frames per second) of the video. Specifically, Seq0001~Seq0005 is 25 frames per second, the interval between two frames is 40 ms. Seq0006~Seq0010 is 30 frames per second, that is, the interval between two frames is 33 ms.

RC1.12: [Data acquisition] Line 1 §1: "ant" instead of "ants".
AR1.12: "ants" has been modified to "ant". The comparison before and after modification is as follows (see details in lines 99-100 of the revised manuscript):
Origin: "We collect 50 workers of Japanese arched ants species …".
Revision: "We collect 50 workers of Japanese arched ant species …".

RC1.13: [Data acquisition] Regarding the links, https://cn.depositphotos.com/home.html and https://data.mendeley.com/datasets/9ws98g4npw/3: How stable are those links? Does the journal require Dryad/Github or another repository?
AR1.13: We removed the first link because its information was already included in our dataset. For the second link, the Mendeley Data continues to provide open-source data repositories to researchers. They claim on their official website that the DOI of this dataset is available always: "To ensure the highest level of integrity and security possible , data is stored on Amazon's S3 servers in Ireland. Our service was extensively penetration tested and received certification. Additionally, your published datasets are archived with Data Archiving and Network Services (DANS) to preserve your data over the long term. DANS is a long-term archiving provider, which is an institute of the Dutch Academy KNAW, and of the Netherlands' national research council, NWO. We contract with DANS to archive all valid published datasets in perpetuity. The agreement ensures that the DOIs we provide for datasets will always

resolve to a web page, where the dataset metadata and files will be available. Data archived at DANS is backed up and stored in three locations for redundancy."
Besides, the journal allows us to choose other repositories.

RC1.14: [Det folder] Provide the information you want to show and include the source between parenthesis. Do not start with the "The 'det' folder…"
AR1.14: Thank you for this suggestion, according to your suggestion, we modified the expression and the comparison before and after modification is as follows (see details in lines 134-139 of the revised manuscript):
Origin: "The 'det' folder contains a 'det.txt' file which is the ground truth for detection, recording the location parameters of the ants in all frames, which is similar to multi-object tracking challenge~\cite{leal2015motchallenge}. Each line represents one ant instance, and it contains 7 values as shown in Table~\ref{tab:data_format}."
Revision: "In the same format as the dataset of multi-object tracking challenge~\cite{leal2015motchallenge}, we record information such as the identity and location parameters of all ants in each frame for detection. Such information is stored in a 'det.txt' file in a folder named 'det' in our dataset. Concretely, each line represents one ant instance, and it contains 7 values (also called attributes) as shown in Table~\ref{tab:data_format}."

RC1.15: [Det folder] Line 3: "which is similar to multi-object tracking challenge": And how is it? Similar how?
AR1.15: The multi-object tracking challenge~\cite{leal2015motchallenge} we describe here is a dataset, and the format of our dataset is the same as that. We modified the expression, and the comparison before and after modification is as follows (see details in lines 134-137 of the revised manuscript):
Origin: "The 'det' folder contains a 'det.txt' file which is the ground truth for detection, recording the location parameters of the ants in all frames, which is similar to multi-object tracking challenge~\cite{leal2015motchallenge}."
Revision: "In the same format as the dataset of multi-object tracking challenge~\cite{leal2015motchallenge}, we record information such as the identity and location parameters of all ants in each frame for detection. Such information is stored in a 'det.txt' file in a folder named 'det' in our dataset."
Besides, the specific format can be seen in lines 139-147 or Table 2 of the revised manuscript.

RC1.16: [Det folder] Line 4: "the position of one ant at one instant classified into seven categories (values 1-7)"? instead of "one ant instance and it contains 7 values"
AR1.16: We use 7 attributes to represent each ant instance, including frame number, identity number, bounding box left, bounding box top, bounding box width, bounding box height, and confidence score (see Table 2 for details). However, it seems that there was an ambiguity in our original statement, so we revised it. The comparison before and after modification is as follows (see details in lines 138-139 of the revised manuscript):
Origin: "Each line represents one ant instance, and it contains 7 values as shown in Table~\ref{tab:data_format}."
Revision: "Concretely, each line represents one ant instance, and it contains 7 values (also called attributes) as shown in Table~\ref{tab:data_format}."

RC1.17: [Gt folder] The same for Det folder. Describe the results, and the information, and then reference the source.
AR1.17: Thank you for this suggestion, according to your suggestion, we modified the expression, and the comparison before and after modification is as follows (see details in lines 149-153 of the revised manuscript):
Origin: "The 'gt' folder contains a 'gt.txt' file, which is the ground truth for muti-object tracking. Similar to the 'det.txt' file, it also contains 7 values (see details in Table~\ref{tab:data_format})."
Revision: "In our dataset, we provide groundtruth records for multi-object tracking. These information are stored in a 'gt.txt' file in a folder named 'gt'. Similar to the previous description of the 'det.txt' file, the records of each instance in the 'gt.txt' file also contains 7 values (also called attributes), see Table~\ref{tab:data_format} for details."

RC1.18: [Img folder] The same as the previous comment. Also, after "e.g." there is a

	comma: "e.g.,"<br>AR1.18: Thank you for this suggestion, according to your suggestion, we modified the expression and the comparison before and after modification is as follows (see details in lines 159-160 of the revised manuscript):<br>Origin: "The 'img' folder stores the original image sequence converted from the video."<br>Revision: "In our dataset, we provide the original image sequence converted from the video, which are stored in the 'img' folder."<br>Besides, "e.g."has been modified to "e.g.,", The comparison before and after modification is as follows (see details in lines 161-162 of the revised manuscript):<br>Origin: "All images are converted to JPEG and named sequentially to a 6-digit file name (e.g. 000001.jpg)"<br>Revision: "All images are converted to JPEG and named sequentially to a 6-digit file name (e.g., 000001.jpg)"<br><br>RC1.19: [Analyses] Visually confirmation instead of "Visually confirm"<br>AR1.19: "Visually confirm" has been modified to "Visually confirmation". The comparison before and after modification is as follows (see details in line 164 of the revised manuscript):<br>Origin: "Visually confirm"<br>Revision: "Visually confirmation"<br><br>RC1.20: [Analyses] Please describe how many people did it, if it expected some bias depending on the person that check the images? Also: Is it necessary to do with all images? How much time does it take? Can you all estimate the labor time, is it?<br>AR1.20:<br>Q1: Please describe how many people did it, if it expected some bias depending on the person that check the images?<br>A1: We have 2 staff marking indoor videos and 3 staff marking outdoor videos. And there is only one inspector, so the bias is avoided. (See details in lines 165-168 of the revised manuscript)<br>Q2: Is it necessary to check all images?<br>A2: In the sequence-level checking, all sequences need to be checked. In the image-level checking, Only the sequence that is considered to be of low quality needs to have each frame checked. (See details in Figure 4 and lines 170-181 of the revised manuscript)<br>Q3: How much time does it take? Can you all estimate the labor time, is it?<br>A3: In the sequence-level checking, depending on how many ants are in each video, the time cost ranges from 8 to 10 times the length of the original video sequence. In the image-level checking, the check speed is about 0.5 seconds/ant, and the repair time is about 2 seconds/ant. (See details in lines 177-179 and 184-185 of the revised manuscript)<br>A comparison of the above three questions involving the content before and after the revision is as follows (see details in lines 165-185 of the revised manuscript):<br>Origin: "The ground truth annotations for all image sequences in the dataset were visually confirmed by the data annotation staff. The visual reviewing consists of two aspects, sequence-level (coarse-grained) and image-level (fine-grained). Firstly, staffs performed a coarse-grained review of a single sequence. Specifically, we drew the annotations on the corresponding images, and then converted the image sequence to video. By replaying the video, staff can quickly confirm which segments of the video are poor quality and needed to be re-marked. For each scene, an example image frame is shown in Figure~\ref{fig:tech_val_in} (a) and Figure~\ref{fig:tech_val_out} (a). After that, staff reviewed the quality of annotations frame-by-frame via VisualMarkData. For inaccurate annotations, staff modified manually by using the "Check and modify" function of VisualMarkData (see details in Methods)."<br>Revision: "In all 10 videos, we have 2 staff to mark indoor videos and 3 staff to mark outdoor videos. Furthermore, the ground-truth annotations for all image sequences in the dataset were visually confirmed by one staff. The visual reviewing consists of two aspects, sequence-level (coarse-grained) and image-level (fine-grained). Firstly, the staff performed a coarse-grained review of a single sequence. Specifically, we drew the annotations on the corresponding images, and then converted the image sequence to video. For each scene, an example image frame is shown in Figure~\ref{fig:tech_val_in} (a) and Figure~\ref{fig:tech_val_out} (a). By replaying the video, staff can quickly confirm which segments of the video are of poor quality and needed to be re-marked. Figure~\ref{fig:low_quality} (a) shows an example of a

	segment distinguished as low-quality annotations. The sequence-level checking time consumption per video is 8 to 10 times the original video sequence duration, which depends on the number of ants in the video. After that, staff reviewed the quality of annotations frame-by-frame via VisualMarkData. For inaccurate annotations, staff modified manually by using the "Check and modify" function of VisualMarkData (see details in Methods). Figure~\ref{fig:low_quality} (b) shows the modified annotations. The image-level checking speed is about 0.5 sec per ant instance while correction takes about 2 sec per ant instance."<br><br>RC1.21: [Analyses] After the equation 3: "is set to o": I guess this is an "o" and not a "0" (zero).<br>AR1.21: Here v0 is set to zero, because we assume that the ants are stationary at the initial moment. We added this assumption after the expression. The comparison before and after modification is as follows (see details in lines 187-188 of the revised manuscript):<br>Origin: "Where, the v_{0} is set to 0."<br>Revision: "Where, the v_{0} is set to 0, i.e. we assumed that the ants were stationary at the initial moment."<br><br>RC1.22: [Discussion] Lines 9-11 §1: Where are the Python Scripts available?<br>AR1.22: Links to the Python scripts have been added now. The comparison before and after modification is as follows (see details in lines 207-210 of the revised manuscript):<br>Origin: "Alongside, we have provided publicly available Python Scripts to illustrate the analysis of data as well as usage of the data."<br>Revision: "Alongside, we have provided publicly available Python Scripts at ~\url{https://github.com/holmescao/ANTS_marking_and_analysis_tools}, to illustrate the analysis of data as well as usage of the data."<br><br>RC1.23: [Discussion] Line 15: "the" instead of "our".<br>AR1.23: “our” has been modified to “the”. The comparison before and after modification is as follows (see details in line 214 of the revised manuscript):<br>Origin: "… evaluate the tracking accuracy on our dataset."<br>Revision: "… evaluate the tracking accuracy on the dataset."<br><br>RC1.24: [Discussion] Lines 1-3 §2: How it would be made? When it would be available? I think that promising something in a paper is not appropriate. Maybe the authors can only tell something parsimonious like: "it is possible that some updates will be done in the future".<br>AR1.24: We highly agree with your suggestion, and we modified the sentence expression and the comparison before and after modification is as follows (see details in lines 218-220 of the revised manuscript):<br>Origin: "In the future, we will enrich the VisualMarkData with more features to reduce the difficulty of marking and improve the efficiency of marking."<br>Revision: "In the future, it is possible that VisualMarkData software will be made some updates to reduce the difficulty of marking and improve the efficiency of marking."<br><br>RC1.25: [Potential implications] It is a bit vague about the implications (and also applications). Please provide citations of previous articles that used the manual tracking and conclusions they made and mention how this technique can improve the reach of these studies in terms of investigating outdoor nests and also more replicates.<br>AR1.25: According to your suggestion, we modified the potential implications section (The current version is called Potential usage of dataset). The comparison before and after modification is as follows (see details in lines 237-270 of the revised manuscript):<br>Origin: "Swarm behavior is one of the most important features of social insects, which has important significance for the study of embodied intelligence~\cite{tiacharoen2012design}. Specifically, social insects often tend to cluster into a colony~\cite{vandermeer2008clusters}, which forms a complex dynamical system together with the surrounding environment~\cite{balch2001automatically}. So far, researchers do not know enough about the mechanisms behind swarm behaviors of social insects. We believe our image sequence marking software and dataset could facilitate the analysis of ant colony behavior leading to the development of embodied intelligence."<br>Revision: " Swarming behavior is one of the most important features of social insects~\cite{vandermeer2008clusters}, often involving division of

Powered by Editorial Manager® and ProduXion Manager® from Aries Systems Corporation

labor~\cite{holldobler1990ants}, task specialization~\cite{whitehouse1996ant}, distributed problem solving~\cite{vaughan2000whistling}, etc. To reveal the mechanisms behind swarming behavior requires observing insect colonies over long periods of time as well as recording the motion trajectory of each individual~\cite{poff2012efficient}. Before the advance of computer vision technology, biologists utilize manual tracking to study insect behaviors~\cite{bond1980optimal, deffernez1990analysis}. Since manual recording is time-consuming and laborious, biologists focus only on individual behavioral studies, including foraging activity~\cite{deffernez1990analysis} and prey avoidance~\cite{bond1980optimal}, etc. In recent years, in order to enable rapid tracking of the activities of multiple insects simultaneously, automated image-based tracking techniques are employed, and a lot of attempts are made to improve the accuracy of tracking~\cite{khan2005mcmc, khan2006mcmc, oh2006parameterized, veeraraghavan2008shape, fletcher2011multiple}. These techniques have assisted biologists to discover some colony mechanisms. For example, Balch T et al~\cite{balch2001automatically} found that a number of ants would interact at the entrance of the nest when some find food nearby. However, current studies are almost limited to laboratory settings with clean backgrounds. Such approaches disregard the influence of the environment surrounding the insect colony, including potential predators~\cite{feener1990defense}, obstacles on the road~\cite{loreto2013foraging}, etc. In contrast, we provide labeled motion trajectories of ant colonies active outdoors, containing a variety of scenes. These data can be used to train deep learning models for automated tracking ants in natural environments. Moreover, we already used indoor/Japanese arched ants' images as the training set in our previous work~\cite{cao2020online} and tested our model on outdoor/black ant images (Seq0010), achieving a tracking accuracy up to 92\%. Vice versa, we also conducted experiments using outdoor images as the training set and indoor images as the test set, which are presented in a method manuscript that we are preparing~\cite{wu2022swarm}, which can be found at arXiv. Hence, it will help biologists to quantify and analyze the foraging patterns of ant colonies in natural environments, such as foraging strategies, partner gathering, and collaborative transportation."

RC1.26: [Methods] More information is required here (or can be placed in the Data Description section). Which ant species do the ant colonies belong to? Which size (more or less) do the ant workers have? Outside or inside the nest? Close to the entrance? What angle of view was used, i.e., distance from ground? The height where the camera was placed? Which camera was used? Area of the frames? Description of the background? The background was cleaned prior to the recording? Etc.
AR1.26: We added the information you suggested and below are the answers to your questions.
Q1: Which ant species do the ant colonies belong to?
R1: Column 7 in the bottom part of Table 1, and lines 96 and 111 of the revised manuscript describe the species of ants of each video.
Q2: Which size (more or less) do the ant workers have?
R2: The ants in each scene are all worker ants, see lines 99-100 and 112-114 in the revised manuscript for details.
Q3: Outside or inside the nest?
R3: No nest in the indoor scenes. Outdoor scenes are filmed outside the nest except for Seq0010. See lines 103-104 and 121-122 in the revised manuscript for details.
Q4: Close to the entrance?
R4: Only the four video scenes of outdoor scenes are close to the entrance, including Seq0006 to Seq0009. See Table 1 and lines 121-122 of the revised manuscript for details.
Q5&Q6: What angle of view was used, i.e., distance from ground? The height where the camera was placed?
R5&A6: Table 1, Figure 5, and lines 281-285 and 297-301 of the revised manuscript describe the camera angle, the field of view of the camera, and the height of the camera from the ground for each video sequence.
Q7: Which camera was used?
A7: Table 1, lines 278-280 and 295-296 of the revised manuscript describe the camera types.
Q8：Area of the frames?
A8: Table 1 gives the area of each scene (length times width). Figure 5, Equation 4, and lines 285-290, 301-303 of the revised manuscript describe how the area is

Powered by Editorial Manager® and ProduXion Manager® from Aries Systems Corporation

	calculated.<br>Q9&Q10：Description of the background? The background was cleaned prior to the recording?<br>A9&A10: Lines 103-104 and 116-122 of the revised manuscript describe the backgrounds of scenes.<br>Besides, we added information about the date, location, and temperature during the filming, as detailed in Table 1.<br><br>RC1.27: [Methods] Figure 1: Figure 1 was never referenced in the text. Line 4, Caption of Figure 1: Describe each of the three visualization tools to verify the data quality.<br>AR1.27: We have added the mention of Figure 1 in 2 places of the context. The added information is as follow (see details in lines 80-81 and 307-308 of the revised manuscript):<br>Add: "Then utilizing VisualMarkData and following the process shown in Figure~\ref{fig:pipeline}, …"<br>Add: "The overall annotation pipeline for the dataset using this software is shown in Figure~\ref{fig:pipeline}."<br>About caption of Figure 1, we added detail descriptions of three Python scripts. The comparison before and after modification is as follows (please see the description of Figure 1 lines 4-5):<br>Origin: "Additionally, we provide three visualization tools to verify the data quality."<br>Revision: "Additionally, three Python scripts are provided to generate three visualization results to verify the quality of the data, including the trajectories drawn on the original graph, the heat map of motion velocity, and the histogram of the frequency distribution of motion velocity."<br><br>RC1.28: [Methods] Figure 2: I suggest changing Velocity to Speed, prefer the scalar rather than the vector.<br>AR1.28: “Velocity” in Figure 2 has been modified to “Speed”, as detailed in Figure 2. Besides, all the "velocity" in the manuscript have been replaced by "speed”.<br><br>RC1.29: [Methods] Line 2, Caption of Figure 2: "five consecutive sequences" instead of "5 sequences" and then remove ", respectively". "Histogram of the frequency of ant speeds in cm/s for indoor sequences" instead of "Ant speed histograms per indoor sequence".<br>AR1.29: We fully adopt your suggestions. The comparison before and after modification is as follows (see details in Figure 2 lines 1-2):<br>Origin: "(b) Speed distributions in image space for 5 sequences of indoor scenes, respectively. (c) Ant speed histograms per indoor sequence."<br>Revision: "(b) Speed distributions in image space for five consecutive sequences of indoor scenes. (c) Histogram of the frequency of ant speeds in cm/s for indoor sequences."<br><br>RC1.30: [Methods] Table 1: Describe the meaning of each column, Sequence is ...XXX, FPS is ...XXX, etc. Maybe "statistics" is not the appropriate term; prefer the use of "description" or something like that.<br>AR1.30: We described the meaning of each column and changed "Statistics" to "Description". It should be noted that we expanded the column of Table 1 based on other comments. The information before and after the modification of caption is as follows (see details in Table 1):<br>Origin: "Statistics of ant videos with annotations in indoor and outdoor scenes."<br>Revision: "Description of ant videos with annotations in indoor and outdoor scenes. Top part provides filming details. Sequence = Name of video for each colony. Angle = Angle of the camera during filming. Height = Height of camera from the ground. Temp = Local temperature during filming. Datetime = Date and time of the filming. Location = Location of the filming. Camera = Camera type. Bottom part provides a description of ant videos with annotations. FPS = Frame rate of the video. Resolution = The resolution of the video. Length = The number of frames of the video, with the duration in parentheses. Ants = The number of ants with different IDs that appear in the video. Annotations = The number of ants instances labeled in the video. Species = The ant species. Entrance = Whether the colony is active at the nest entrance. Area = The area of the filmed scene. Note that the camera's angle of view is 16° and 7.5° in the horizontal and vertical directions, respectively, which are not represented in the table."

Powered by Editorial Manager® and ProduXion Manager® from Aries Systems Corporation

RC1.31: [Methods] Table 2: Maybe the files could be provided as an appendix for the paper.
AR1.31: Thank you for your suggestion. However, due to each file contains thousands of lines, it would be inconvenient to add the files directly to the manuscript's appendix. Thus, we published the files in https://data.mendeley.com/datasets/9ws98g4npw/3, which is convenient for users to view and download.

Reviewer #2
RC2.0: This paper developed the image sequence marking software VisualMarkData and tracked ants' locomotion in indoor and outdoor environments. All data of these images are available. Developing new tracking software is important for animal behaviour and computational biology. However, it wasn't easy to follow the novelty of this study and its importance as biological research.
AR2.0: We thank the reviewer for the kind comments.

RC2.1: [Major comments] What is the novel point of the image sequence marking software VisualMarkData? The authors have mentioned outdoor and indoor, but how did you overcome this difficulty?
AR2.1: For the first question, the main contribution of VisualMarkData is that it offers: (1) a comprehensive annotation of states at individual-target as well as group-target level; (2) representation of annotations (together with their IDs and behavior labels) of multiple targets and multiple groups in a simple-to-access matrix format; (3) a simple and efficient visualization during annotation, which presents the annotation information of the previous frame as a reference and then only requires clicking on the center point of each target to complete the annotation; and (4) a windows-based friendly graphical user interface that minimizes labor and maximizes annotation quality. We revised the abstract and the introduction (current version is context) about the VisualMarkData to highlight these contributions, as described in lines 4-10 and 62-74, respectively, of the revised manuscript.
In addition, since the type of our manuscript is "Data Note", we focus on providing a detailed approach to data production, validation, and potential reuse, which is in line with the instructions on the journal's website, see https://academic.oup.com/gigascience/ pages/data_note#Criteria. We are the first to construct the ant colony activity dataset with annotations with multiple species and colonies in both indoor and outdoor environments. Specifically, the dataset contains 3 species and 10 colonies with a total of 5,354 frames, 712 ants, and 114,112 labels. Where the revision of the descriptions of the abstract is shown below (It can also be found in lines 4-10 of the revised manuscript abstract).
Origin: "And we develop an image sequence marking software named VisualMarkData, which enables us to provide annotations of ants in the video."
Revision: "And we develop an image sequence marking software named VisualMarkData, which enables us to provide annotations of ants in the video: (1) offers a comprehensive annotation of states at individual-target as well as colony-target level; (2) provides a simple matrix format to represent multiple targets and multiple groups of annotations (along with their IDs and behavior labels); (3) during the annotation process, we propose a simple and effective visualization that takes the annotation information of the previous frame as a reference and then simply clicks on the center point of each target to complete the annotation; and (4) we develop a user-friendly windows-based GUI to minimize labor and maximize annotation quality."
Where the revision of the descriptions of the introduction (called context in the current version) is shown below (it can also be found in lines 62-74 of the revised manuscript).
Origin: "Besides, we develop an image sequence mark software named VisualMarkData, which is used to mark the pixel patches covered by ants in each frame of the video."
Revision: "With respect to the tool, we propose the VisualMarkData, which allows users to generate ground-truth information of multi-target motion trajectories in video sequences. Specifically, VisualMarkData offers: (1) a comprehensive annotation of states at individual-target as well as group-target level; (2) representation of annotations (together with their IDs and behavior labels) of multiple targets and multiple groups in a simple-to-access matrix format; (3) a simple and efficient visualization during annotation, which presents the annotation information of the previous frame as a reference and then only requires clicking on the center point of each target to complete the annotation; and (4) a windows-based friendly graphical

user interface that minimizes labor and maximizes annotation quality."
For the second question, there is no difference in the way we annotate the indoor and outdoor scene videos, and the detailed annotation method is described in lines 311-354 of the revised manuscript.
As for the method of data acquisition, we provide a detailed description in the Data Description section, including both indoor and outdoor (added in the current version) ways, which are described in lines 95-124 of the revised manuscript.

RC2.2: [Major comments] What is the purpose of providing your dataset for training or future research? If it is for training, the authors will show that training using indoor/Japanese arched ants' images can be useful for tracking outdoor/black ant images or vice versa. If authors want other researchers re-analyze these data for any reason, basic information is lacking. The species name is very important; the authors need to mention it. It is better to provide the temperature, date, time, and location of the colony when they took video in the outdoor environment.
AR2.2: The main purpose of providing this data set is to train automated models for biologists to better understand the clustering behavior of insects, as described in lines 6-9 of the revised version. In the meanwhile, our dataset can also be used for future research.
For training, we already used indoor/Japanese arched ants' images as the training set in our previous work~\cite{cao2020online} and tested our model on outdoor/black ant images, achieving a tracking accuracy up to 92%. Vice versa, we also conducted experiments using outdoor images as the training set and indoor images as the test set, which are presented in a method manuscript that we are preparing~\cite{wu2022swarm}, which can be found at arXiv. These details are added in lines 260-267 of the Potential implications (named Potential usage of dataset in the current version) section of the revised manuscript.
For future research, we added relevant information so that other researchers re-analyze these data for any reason. Additional information includes:
•Colony size
•Species name
•Nest exist or not in the scene
•Relationship between the scene and the entrance of the nest
•The background of the scene is processed or not
•The background description of the filming scene
•Temperature during the filming
•Filming date and time
•Filming location
•Camera type and parameter
•Camera angle
•Filming height of camera
•The field of view of camera
•The area of the filming scene and its measure method
This information is supplemented in Table 1, Figure 5, Equation 4, lines 95-124, 277-290 and 295-303 of the revised manuscript.

RC2.3: [Major comments] Why did you use two species? Did you collect two species of ants and record their behaviour under both indoor and outdoor conditions? If not, why? Can we use data of different ant species as training data? For example, if you use data of Japanese arched ants as training, can you detect the black ant?
AR2.3: We use three species, including Japanese arched ants (also called Camponotus japonicus ants)~\cite{nakanishi2009sex, he2011bacteria, nishikawa2012higher}, Little black ants~\cite{wang2015fatty, thompson2019ants} and Carpenter ants~\cite{sanders1964biology, carney1969behavioral, carlin1986kin}, which are widely studied by behavioral ecologists and social biologists. Since Japanese arched ants are often domesticated, they are suitable for observation in laboratory environments. As for the other two species of ants, we only record their behavior outdoors and do not catch them in laboratory environments so as not to disturb their normal life. It is in accordance with academic ethics that ant citizens should not be violated casually to prevent ecological damage. In the revised manuscript, the species of ants in each video are shown in Table 1, and the relevant descriptions can be found in lines 96-99 and 111-114.
In addition, we can use data of different ant species as training data. we already used indoor/Japanese arched ants' images as the training set in our previous

work~\cite{cao2020online} and tested our model on outdoor/black ant images, achieving a tracking accuracy up to 92%. Vice versa, we also conducted experiments using outdoor images as the training set and indoor images as the test set, which are presented in a method manuscript that we are preparing~\cite{wu2022swarm}, which can be found at arXiv. These details are added in lines 260-267 of the Potential implications (named Potential usage of dataset in the current version) section of the revised manuscript.

RC2.4: [Minor comments] Indoor, did you detect ten ants all time? What was the accuracy of the detection?
AR2.4: VisualMarkData is a marking tool with a graphical user interface, which can facilitate users to mark targets in the scene quickly. We marked the location and identity information of each ant in each frame, as described in lines 10-11 of the abstract. Thus, we didn't use neural network to detect ants in this manuscript. Our contribution is to manually mark the trajectories of ants using VisualMarkData to obtain ground-truth. As a "Data Note" type article, the introduction to the dataset and its production method is our main contribution. As for the accuracy of the annotations, all the image sequences in the dataset were visually confirmed by a data annotator. More detailed information is described in the visual confirmation subsection of the revised manuscript (lines 165-185).

RC2.5: [Minor comments] Indoor environment: How many colonies did you collect? How did you record the video outdoor? I couldn't find this information in the Outdoor environment and Hardware devices for acquiring raw data.
AR2.5: For indoor environment, we collected 50 workers of Japanese arched ant species and randomly divided them into 5 colonies of 10 ants each, as described in lines 99-105 in the revised manuscript. We have supplemented information such as video record of the outdoor environment and hardware devices for acquiring raw data in the revised manuscript (see details in lines 293-303 of the revised manuscript).

RC2.6: [Minor comments] Visually confirm: I am unsure of the meaning of poor quality and needed to be re-marked. Can you show the use before and after of re-marked image? In addition, did you annotate just an ant or keep the ID of an ant? If you keep an ant's ID, how did you do?
AC2.6: To make the meaning of mark quality and re-marking clearer and easier to understand, we add an example of re-marking a segment, as shown in Figure 4. Besides, we always keep one ant's ID while marking. Specifically, the user only marks the same object until finishes the entire image sequence, and then the user can focus on another object and repeating the same operation. In order to help users quickly locate the same target in the next frame, the marked location on the previous frame will be displayed with a green-dotted. Above description can be seen in lines 333-343 of the revised manuscript.

Reviewer #3
RC3.0: Authors make available a new dataset of ant colony videos, containing scenes from 10 different ant colonies, with indoor and outdoor variants, to aid in the preparation of better computer vision approaches to track ants. Indeed, those methods require a good amount of labeled data, which is very time consuming and error prone in such insects. Videos seem to have a good variety of resolution and number of ants (crowding). They also provide an open-source tagging software to aid in the augmentation of such datasets. Code and data is publically available.
AR3.0: Thank you for your careful evaluation of this manuscript.

RC3.1: Given the details above, I don't have major concerns about this work. I would only recommend checking English in some sentences, but it is on average very well written.
AR3.1: We have made any necessary modifications and check that intended meaning has been retained. And here we did not list the changes but marked in blue (add) and red (delete) in revised paper. Please see the revised manuscript with track changes for details.

RC3.2: In addition, others are also working on improving the capture of such insects in natural environments, so it could be useful to cite them as well. One in particular

Powered by Editorial Manager® and ProduXion Manager® from Aries Systems Corporation

	provides open-hardware scheme to make it reproducible: Sabattini, J. A., Reta, J. M., Bugnon, L. A., Cerrudo, J. I., Sabattini, R. A., Peñalva, A., ... & Sturniolo, F. (2022). AntVideoRecord: Autonomous system to capture the locomotor activity of leafcutter ants. HardwareX, 11, e00270.<br>AR3.2: Thanks for the comments and suggestions. In the introduction (named context in the current version), we add two works on capturing insects in the natural environment and modify the relevant contents. The comparison before and after modification is as follows (see details in lines 38-44 of the revised manuscript):<br>Origin: "A critical requirement for the development of these models is access to the datasets containing motion trajectories of insects in the video. To the best of our knowledge, however, the current studies are all using only one outdoor-scene dataset, which is lack of data diversity."<br>Revision: "A critical requirement for the development of these models is access to the datasets containing motion trajectories of insects in the video. Several works are working on improving the capture of such insects in natural environments~\cite{imirzian2019automated, sabattini2022antvideorecord}. To the best of our knowledge, however, only a few works~\cite{imirzian2019automated, cao2020online} annotate motion trajectories in videos, and all use only a single outdoor scene sequence, which lacks data diversity."
**Additional Information:**	
**Question**	**Response**
Are you submitting this manuscript to a special series or article collection?	No
**Experimental design and statistics**<br><br>Full details of the experimental design and statistical methods used should be given in the Methods section, as detailed in our Minimum Standards Reporting Checklist. Information essential to interpreting the data presented should be made available in the figure legends.<br><br>Have you included all the information requested in your manuscript?	Yes
**Resources**<br><br>A description of all resources used, including antibodies, cell lines, animals and software tools, with enough information to allow them to be uniquely identified, should be included in the Methods section. Authors are strongly encouraged to cite Research Resource Identifiers (RRIDs) for antibodies, model organisms and tools, where possible.<br><br>Have you included the information requested as detailed in our Minimum	Yes

Powered by Editorial Manager® and ProduXion Manager® from Aries Systems Corporation

Standards Reporting Checklist?	
**Availability of data and materials**<br><br>All datasets and code on which the conclusions of the paper rely must be either included in your submission or deposited in publicly available repositories (where available and ethically appropriate), referencing such data using a unique identifier in the references and in the "Availability of Data and Materials" section of your manuscript.<br><br>Have you have met the above requirement as detailed in our Minimum Standards Reporting Checklist?	Yes

Powered by Editorial Manager® and ProduXion Manager® from Aries Systems Corporation

revised manuscript
Click here to access/download;Manuscript;revised manuscript.pdf

GigaScience, 2017, 1–9

doi: xx.xxxx/xxxx
Manuscript in Preparation
Paper

PAPER

A dataset of ant colonies motion trajectories in indoor and outdoor scenes for social cluster behavior study

Meihong Wu[1,†], Xiaoyan Cao[1,†], Ming Yang[1], Xiaoyu Cao[2] and Shihui Guo[1,*]

[1]School of Informatics, Xiamen University, Xiamen, 361000, China and [2]Chemistry and Chemical Engineering, Xiamen University, Xiamen, 361000, China

[*]guoshihui@xmu.edu.cn
[†]Contributed equally.

Abstract

Motion and interaction of social insects (such as ants) have been studied by many researchers to understand the clustering mechanism. Most studies in the field of ant behavior have only focused on indoor environments (laboratory setup), while outdoor environments (natural environments) are still underexplored. In this paper, for the first time, we collect 10 videos and 3 species of ant colonies from different scenes, including 5 indoor and 5 outdoor scenes. And we develop an image sequence marking software named VisualMarkData, which enables us to provide annotations of ants in the video: (1) offers a comprehensive annotation of states at individual-target as well as colony-target level; (2) provides a simple matrix format to represent multiple targets and multiple groups of annotations (along with their IDs and behavior labels); (3) during the annotation process, we propose a simple and effective visualization that takes the annotation information of the previous frame as a reference and then simply clicks on the center point of each target to complete the annotation; and (4) we develop a user-friendly windows-based GUI to minimize labor and maximize annotation quality. In all 5,354 frames, the location information and the identification number of each ant are recorded for a total of 712 ants and 114,112 annotations. Moreover, we provide visual analysis tools to assess and validate the technical quality and reproducibility of our data. It is hoped that this dataset will contribute to a deeper exploration on the behavior of the ant colony.

Key words: social insects; outdoor scenes; image sequence marking software

Context

Social insects often tend to cluster into a colony [1], which forms a complex social network [2]. From time to time, the social network springs up with self-organized clustering behaviors, including division of labor [3], task specialization [4], and distributed problem solving [5]. Biologists analyze the evolution of social network to understand the clustering behavior of insects [6], thus promoting the development of relevant modern applications, such as wireless communication [7] and cluster intelligent control [8]. The key requirement of this research is the ability to track the motions and interactions of each individual robustly and accurately.

Until the late 20th century, biologists still manually tracked the motion trajectories on the video to guarantee the accuracy of the marking. However, they have to track each individual at one time, which might mean watching the entire video 50 times or more in a crowded scene [9]. Manually tracking is time-consuming and prone to human error. It becomes an inhibiting factor in obtaining the complete and accurate dataset required to analyze the evolution of social networks. Therefore, in the past two decades, attempts have been made to automate the tracking process for social insects utilizing computer vision (CV) techniques [10, 11, 12, 13, 14].

Traditional CV techniques release researchers from manual work through approaches such as foreground segmentation algorithm [15], temporal difference method [10] and hungarian algorithm [16]. Such approaches, however, have failed to address the noise in the image [17], resulting in the limitation that a laboratory environment with a clean background is needed. Nevertheless, many scientifically valuable results are obtained in nature rather than laboratory environment [18, 19, 20, 21].

Compiled on: July 27, 2022.
Draft manuscript prepared by the author.

Fortunately, with the emergence of deep learning, CV techniques are already capable of addressing many complex tasks [22, 23, 24], which brings a piece of good news to automated insect tracking in outdoor scenes. Several studies have explored automated multi-ant tracking in outdoor scenes using deep learning-based models [25, 26]. Experimental results demonstrate that these models could be scaled up into a cost-effective alternative to traditional manual tracking methods which are typically costly and/or labor intensive [25, 26]. A critical requirement for the development of these models is access to the datasets containing annotations of motion trajectories of insects in the video. Several works are working on improving the capture of such insects in natural environments [25, 27]. To the best of our knowledge, however, only a few works [25, 26] annotate motion trajectories in videos, and both use only a single outdoor scene sequence, which lacks data diversity.

Considering the importance of annotating targets in videos, some annotation tools have been proposed over the years including LabelME [28], VATIC [29], ViPER [30], and ViTBAT [31]. Except for ViTBAT, other tools are generally more suitable for annotating ground-truth information at the individual target level in terms of tracking targets. As for ViTBAT supports annotating a group of targets but requires much effort to set up rectangular boxes with different sizes for each target. Additionally, it will not display the annotation results of the previous frame in the current frame, which makes it difficult for the user to identify the same target during the annotation process of the video sequence. Moreover, it only supports Linux systems, which is difficult to use for non-computer background biology researchers. In our opinion, as a marking tool, it is desired to be user-friendly, minimize human effort and maximize annotation.

To summarise, the contributions of our work are mainly in the aspects of tool and dataset.

With respect to the tool, we propose the VisualMarkData, which allows users to generate ground-truth information of multi-target motion trajectories in video sequences. Specifically, VisualMarkData offers: (1) a comprehensive annotation of states at individual-target as well as group-target level; (2) representation of annotations (together with their IDs and behavior labels) of multiple targets and multiple groups in a simple-to-access matrix format; (3) a simple and efficient visualization during annotation, which presents the annotation information of the previous frame as a reference and then only requires clicking on the center point of each target to complete the annotation; and (4) a windows-based friendly graphical user interface that minimizes labor and maximizes annotation quality.

With regard to the dataset, we are the first to construct the ant colony activity dataset with annotations with multiple species and colonies in both indoor and outdoor environments. Concretely, we build equipment for video acquisition in various environments and obtain a number of different ant colony activity videos, including 3 species and 10 colonies. Then utilizing VisualMarkData and following the process shown in Figure 1, a large-scale dataset of ant colony activity with annotations is constructed. The total size of the dataset is 5,354 frames, 712 ants, and 114,112 labels. We believe that the dataset will benefit future research on social insect behavior analysis.

Data Description

We collect 10 videos that record activities of different ant colonies, including colonies from both indoor and outdoor scenes. To help us mark the motion trajectories, we develop an image sequence marking software called VisualMarkData.

After spending a large quantity of time and effort, we obtain a dataset with 5,354 frames and 114,112 annotations. Table 1 describes the dataset in detail.

Data acquisition

Indoor environment

Japanese arched ants (also called Camponotus japonicus ants) are widely studied by behavioral ecologists and social biologists [32, 33, 34]. And these ants are often domesticated, thus they are suitable for observation in laboratory environments. We collected 50 workers of Japanese arched ant species, which ranged from 7.4 to 13.8 *mm* in body length [35]. We constructed a laboratory environment for them, including a stable light source, stable temperature and a transparent plastic container. And the background of the container was clean and without the nest. We randomly divided them into 5 colonies of ants each. Then, we loaded each colony into the container in turns and filmed their activities with a high-resolution video camera. These videos were named Seq0001 to Seq0005. These filmings took place on April 15, 2019, in the morning in Xiamen, Fujian, China. More detailed information is provided in Table 1.

Outdoor environment

Little black ants [36, 37] and Carpenter ants [38, 39, 40], are the focus of research by behavioral ecologists and sociobiologists. We acquired five videos from five ant colonies in different outdoor environments, each colony ranging from 73 to 193 workers. The species of these ant colonies were Carpenter and Little black ants, and their body length was between 8 and 10 mm [41]. We named the obtained videos Seq0006 to Seq0010. Concrete and uneven stones were in the background of Seq0006. Seq0007 and Seq0008 were filmed in the dry grass. As for Seq0009 and Seq0010, they were filmed on a dirt road and a rocky road, respectively. And the backgrounds of scenes were without any processing. Except for Seq0010, the scenes of the other four videos were taken at the entrance of the nest. More informative details about the time, location, and temperature of each film are shown in Table 1.

Data Records

The dataset consists of 10 image sequences from different scenes in JPEG digital image format, which is publicly available on https://data.mendeley.com/datasets/9ws98g4npw/3. Alongside, we provide annotations marked by VisualMarkData for all image sequences in the form of text. In the dataset, the images and annotations of each sequence are organized into three folders are named 'det', 'gt', and 'img'.

Det folder

In the same format as the dataset of multi-object tracking challenge [42], we record information such as the identity and location parameters of all ants in each frame for detection. Such information is stored in a 'det.txt' file in a folder named 'det' in our dataset. Concretely, each line represents one ant instance, and it contains 7 values (also called attributes) as shown in Table 2. The first number indicates in which frame the ant appears (sorted by ascending order), while the second number identifies that ant as belonging to a trajectory by assigning a unique ID (set to -1 in a detection file, as no ID is assigned yet). The next four numbers indicate the location of the bounding box of the ant in 2D image coordinates. The location is indicated by the top-left corner as well as the width and height of the bounding box. This is followed by a single number, which denotes the confidence score.

Gt folder

In our dataset, we provide ground-truth records for multi-object tracking. These information are stored in a 'gt.txt' file in a folder named 'gt'. Similar to the previous description of the 'det.txt' file, the records of each instance in the 'gt.txt' file also contains 7 values (also called attributes), see Table 2 for details. The difference compared to the 'det.txt' file is that the second number in the 'gt.txt' file

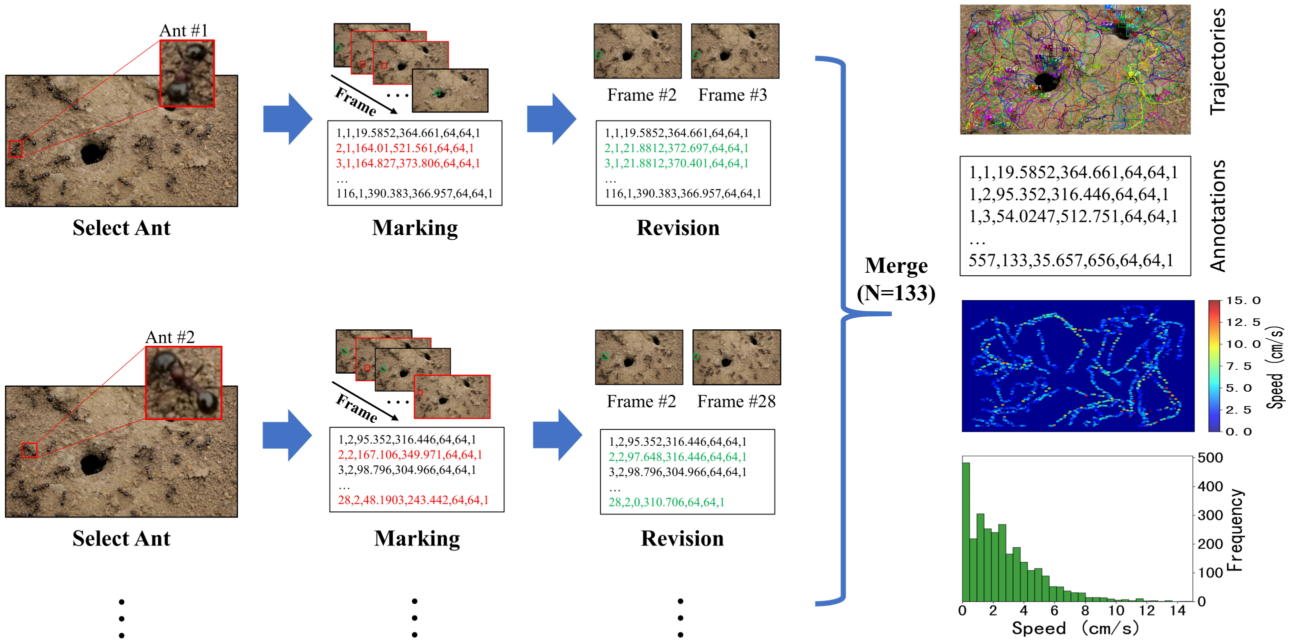

Figure 1. The pipeline for marking motion trajectories of ants in an image sequence, taking an outdoor scene as an example. A total of 133 ants appear in this image sequence, and we select one ant to be marked in each epoch. We use a square bounding box to point out the ant's location and record the relevant parameters at the same time. After all ants of the entire image sequence have been marked, we check the quality of the annotations frame by frame so that wrong annotations (red font) can be corrected (green font). Then, we merge all the annotations of the image sequence into one file. Additionally, three Python scripts are provided to generate three visualization results to verify the quality of the data, including the trajectories drawn on the original graph, the heat map of motion speed, and the histogram of the frequency distribution of motion speed.

Filming details							
Scene	Sequence	Angle	Height	Temp	Datetime	Location	Camera
Indoor	Seq0001	0°	30cm	24°C-26°C	2019/04/15 morning	Xiamen, Fujian, China	Panasonic GX 85
	Seq0002	0°	30cm	24°C-26°C	2019/04/15 morning	Xiamen, Fujian, China	Panasonic GX 85
	Seq0003	0°	30cm	24°C-26°C	2019/04/15 morning	Xiamen, Fujian, China	Panasonic GX 85
	Seq0004	0°	30cm	24°C-26°C	2019/04/15 morning	Xiamen, Fujian, China	Panasonic GX 85
	Seq0005	0°	30cm	24°C-26°C	2019/04/15 morning	Xiamen, Fujian, China	Panasonic GX 85
Outdoor	Seq0006	45°	30cm	15°C-18°C	2019/06/23 morning	Russian Federation, Saint-Petersburg	Canon 5d
	Seq0007	30°	30cm	30°C-35°C	2019/07/21 morning	Greece, Athens	Canon 5d
	Seq0008	30°	30cm	15°C-18°C	2019/06/23 morning	Russian Federation, Saint-Petersburg	Canon 5d
	Seq0009	30°	30cm	15°C-18°C	2019/06/23 morning	Russian Federation, Saint-Petersburg	Canon 5d
	Seq0010	0°	30cm	15°C-17°C	2019/04/21 morning	United States, Neptune Beach	Canon T3i

Description of videos with annotations									
Scene	Sequence	FPS	Resolution	Length	Ants	Annotations	Species	Entrance	Area
Indoor	Seq0001	25	1920×1080	351 (00:14)	10	3510	Japanese arched ants	no	17cm×8cm
	Seq0002			351 (00:14)	10	3510	Japanese arched ants	no	17cm×8cm
	Seq0003			351 (00:14)	10	3510	Japanese arched ants	no	17cm×8cm
	Seq0004			351 (00:14)	10	3510	Japanese arched ants	no	17cm×8cm
	Seq0005			1001 (00:40)	10	3510	Japanese arched ants	no	17cm×8cm
Outdoor	Seq0006	30	1280×720	600 (00:20)	73	11178	Carpenter ants	yes	17cm×16cm
	Seq0007			677 (00:23)	162	25158	Little black ants	yes	17cm×11cm
	Seq0008			577 (00:19)	133	10280	Carpenter ants	yes	17cm×11cm
	Seq0009			526 (00:18)	193	27902	Carpenter ants	yes	17cm×11cm
	Seq0010			569 (00:19)	101	22044	Little black ants	no	17cm×8cm

Table 1. Description of ant videos with annotations in indoor and outdoor scenes. **Top part provides filming details**. Sequence = Name of video for each colony. Angle = The horizontal angle of the camera during filming. Height = Height of camera from the ground. Temp = Local temperature during filming. Datetime = Date and time of the filming. Location = Location of the filming. Camera = Camera type. **Bottom part provides a description of ant videos with annotations.** FPS = Frame rate of the video. Resolution = The resolution of the video. Length = The number of frames of the video, with the duration in parentheses. Ants = The number of ants with different IDs that appear in the video. Annotations = The number of ants instances labeled in the video. Species = The ant species. Entrance = Whether the colony is active at the nest entrance. Area = The area of the filmed scene. Note that the camera's angle of view is 16° and 7.5° in the horizontal and vertical directions, respectively, which are not represented in the table.

Position	Name	Description
1	Frame number	Indicate at which frame the object is present
2	Identity number	Each ant trajectory is identified by a unique ID (-1 for detections)
3	Bounding box left	Coordinate of the top-left corner of the ant bounding box
4	Bounding box top	Coordinate of the top-left corner of the ant bounding box
5	Bounding box width	Width in pixels of the ant bounding box
6	Bounding box height	Height in pixels of the ant bounding box
7	Confidence score	Indicates how confident the detector is that this instance is a ant. For the ground truth and results, it acts as a flag whether the entry is to be considered

Table 2. Data format for annotation files, both for 'det.txt' and 'gt.txt' files.

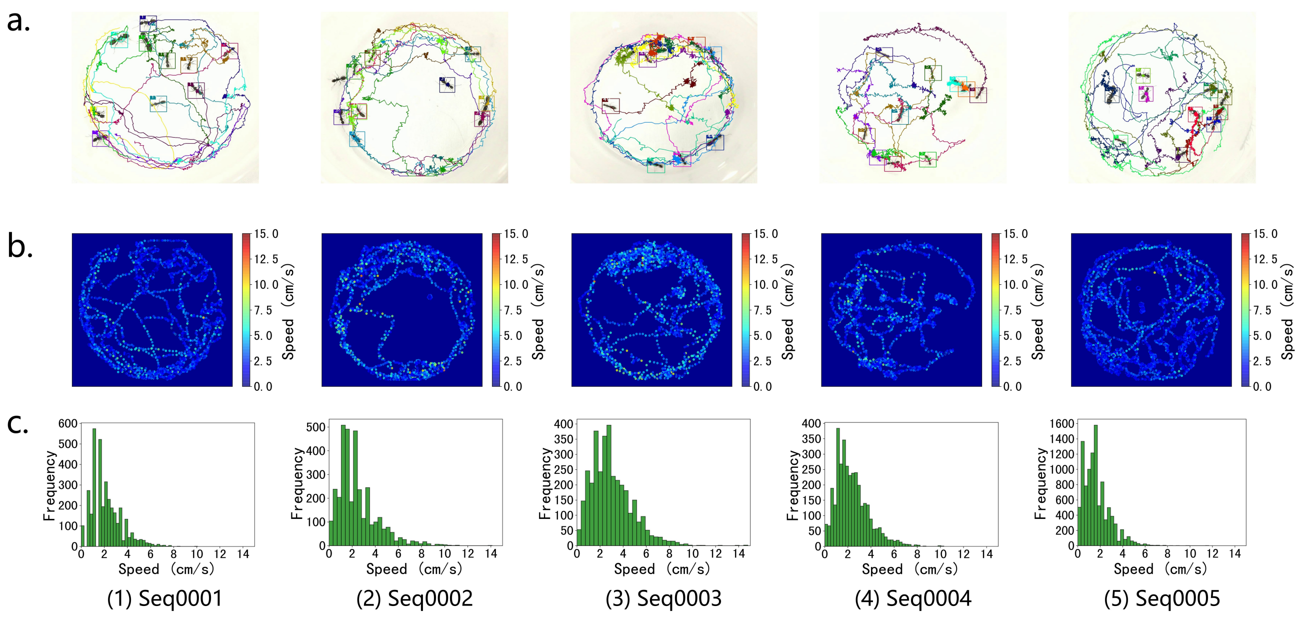

Figure 2. Visual analysis of the marking results on indoor ant videos. (a) Visualization of motion trajectories of the ants for each sequence of the indoor scene. (b) Speed distributions in image space for five sequences of indoor scenes. (c) Histogram of the frequency of ant speeds in *cm/s* for indoor sequences.

records the ID of an ant as belonging to a trajectory, which provides the key information for implementing multi-ant tracking. Besides, each ant can be assigned to only one trajectory.

Img folder

In our dataset, we provide the original image sequence converted from the video, which are stored in the 'img' folder. All images are converted to JPEG and named sequentially to a 6-digit file name (e.g., 000001.jpg).

Data Validation and quality control

Visually confirmation

In all 10 videos, we have 2 staff to mark indoor videos and 3 staff to mark outdoor videos. Furthermore, the ground-truth annotations for all image sequences in the dataset were visually confirmed by one staff. The visual reviewing consists of two aspects, sequence-level (coarse-grained) and image-level (fine-grained).

Firstly, the staff performed a coarse-grained review of a single sequence. Specifically, we drew the annotations on the corresponding images, and then converted the image sequence to video. For each scene, an example image frame is shown in Figure 2 (a) and Figure 3 (a). By replaying the video, staff can quickly confirm which segments of the video are of poor quality and needed to be re-marked. Figure 4 (a) shows an example of a segment distinguished as low-quality annotations. The sequence-level checking time consumption per video is 8 to 10 times the original video sequence duration, which depends on the number of ants in the video. After that, staff reviewed the quality of annotations frame-by-frame via VisualMarkData. For inaccurate annotations, staff modified manually by using the "Check and modify" function of VisualMarkData (see details in Methods). Figure 4 (b) shows the modified annotations. The image-level checking speed is about 0.5 sec per ant instance while correction takes about 2 sec per ant instance.

Motion speed analysis

Further, to demonstrate the reliability of our dataset, we analyzed the distribution of the movement speed of the ants in our dataset. First, for each ant, we used the 2D Euclidean distance [43] to calculate its pixel distance between two adjacent frames. Therefore, the pixel distance $\triangle ps_t$ of the ant at frame t can be defined by the following equation:

$$\triangle ps_t = \sqrt{(px_t - px_{t-1})^2 + (py_t - py_{t-1})^2} \tag{1}$$

where px_t denotes the pixel position of the ant in the horizontal direction at frame t. Similarly, py_t denotes the pixel position in the vertical direction. To convert the pixel distance to real-world coordinates, we divided the ant's body length L (unit: m) in the real world by body length n (unit: $pixel$) in the image. Thus, the real-world displacement of the ant at frame t, $\triangle s_t$ (unit: m) can be expressed as follows:

$$\triangle s_t = \triangle ps_t \times L/n \tag{2}$$

Since the FPS for a specifc video is a constant f_c, the speed v_t

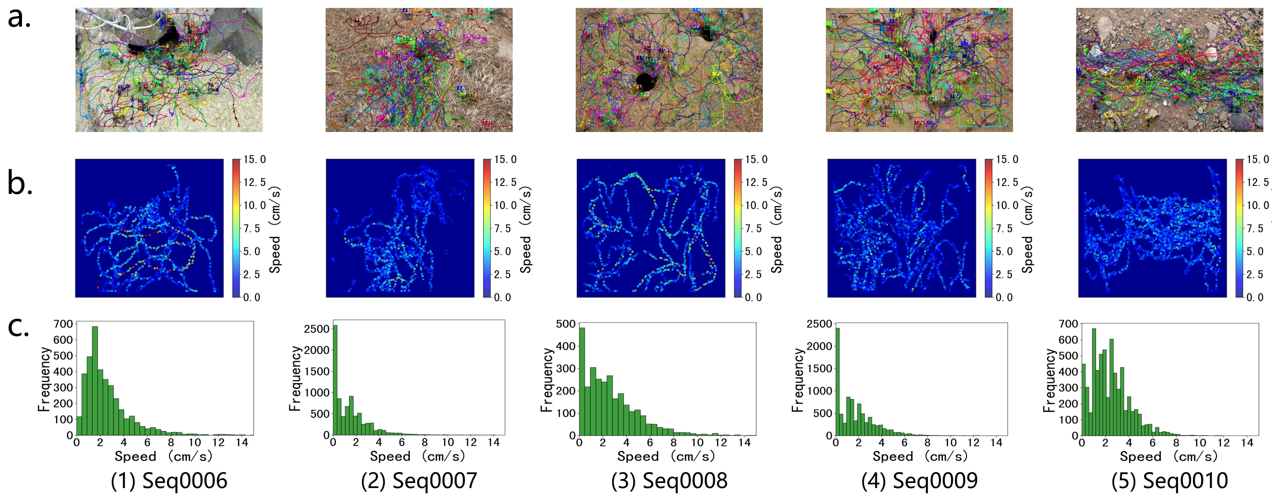

Figure 3. Visual analysis of the marking results on outdoor ant videos. (a) Visualization of motion trajectories of the ants for each sequence of the outdoor scene. (b) Speed distributions in image space for five consecutive sequences of indoor scenes. (c) Histogram of the frequency of ant speeds in cm/s for indoor sequences.

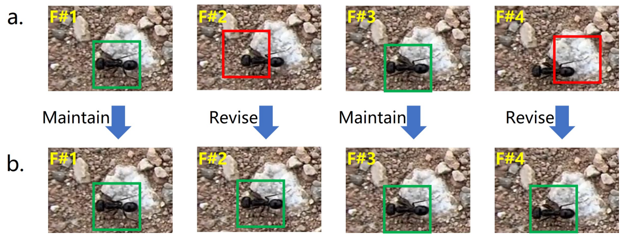

Figure 4. An example of re-marking a segment. (a) indicates the result of marking before revision, where the green and red boxes indicate the high and low quality annotations, respectively. (b) indicates the result after re-marking, where we only need to revise the low quality annotations in (a) to get the high quality annotations (green box).

(unit: $m \cdot s^{-1}$) at frame t can be formulated as:

$$v_t = \frac{\triangle s_t}{1/f_c} \tag{3}$$

Where, the v_0 is set to 0, i.e. we assumed that the ants were stationary at the initial moment. According to the aforementioned equations, combined with the location information of ants in annotations, we can analyze the motion speed of ants in the video, as shown in Figure 2 (b), (c) and Figure 3 (b), (c). Specifically, the overall motion speed of ants in indoor and outdoor scenes are 2.16±1.49 $cm \cdot s^{-1}$ and 1.98±1.84 $cm \cdot s^{-1}$, respectively. These values are within a reasonable range (ants average motion speed is 2.85 $cm \cdot s^{-1}$ under bi-directional traffic condition [44]). This demonstrates that the ant colony activity dataset we collected and marked is real and reliable.

Discussion

The image sequence marking software VisualMarkData is a toolkit with interactive visualization. The goal of the software is to provide a convenient tool for researchers marking movement trajectories of social insects in videos, thus facilitating the study of the behavioral mechanisms of social insects. Additionally, by using the software, researchers will obtain standardized annotation data, as details in the previous section. VisualMarkData is open source, which enables researchers to mark their image sequence datasets of any multi-object motion scenario. Alongside, we have provided publicly available Python Scripts at https://github.com/holmescao/ANTS_marking_and_analysis_tools, to illustrate the analysis of data as well as usage of the data. To visualize and reproduce the results described in the Technical Validation section, we develop two scripts for the researchers. Also, we provide another script to calculate metrics [42] of multi-object tracking that enables any deep learning algorithm to evaluate the tracking accuracy on the dataset. The annotated trajectory data can be used for training and testing of supervised learning models, thus providing a powerful tool for studying a wider range of ant colony behaviors.

In the future, it is possible that VisualMarkData software will be made some updates to reduce the difficulty of marking and improve the efficiency of marking. The software currently marks targets based on their center points, and we are considering introducing stretchable annotation capabilities based on rectangles or ellipses. In addition, the simultaneous annotation of multiple targets in one frame is also a feature worth developing. Along with that, we can introduce semi-automated annotation, i.e., embedding a neural network model into the VisualMarkData, which will automatically predict and annotate objects of the current frame based on the information in the previous frame. Thus, the annotators only need to fine-tune the annotation, which will significantly improve the efficiency of the annotation.

The dataset and VisualMarkData will boost researchers both in biology and computer science to study on behavior of social insects in different environments. We hope that this work will contribute to the potential discovery of ant colony behavioral mechanisms and facilitate the application of the image processing field in biology.

Potential usage of dataset

Swarming behavior is one of the most important features of social insects [1], often involving division of labor [3], task specialization [4], distributed problem solving [5], etc. To reveal the mechanisms behind swarming behavior requires observing insect colonies over long periods of time as well as recording the motion trajectory of each individual [9]. Before the advance of computer vision technology, biologists utilize manual tracking to study insect behaviors [45, 46]. Since manual recording is time-consuming and laborious, biologists focus only on individual behavioral studies, including foraging activity [46] and prey avoidance [45], etc. In recent years, in order to enable rapid tracking of the activities of multiple insects simultaneously, automated image-based tracking techniques are employed, and a lot of attempts are made to improve the accuracy of tracking [10, 11, 12, 13, 14]. These techniques have assisted biologists to discover some colony mechanisms. For example, Balch T et al [2] found that a number of ants would interact

at the entrance of the nest when some find food nearby. However, current studies are almost limited to laboratory settings with clean backgrounds. Such approaches disregard the influence of the environment surrounding the insect colony, including potential predators [47], obstacles on the road [48], etc. In contrast, we provide labeled motion trajectories of ant colonies active outdoors, containing a variety of scenes. These data can be used to train deep learning models for automated tracking ants in natural environments. Moreover, we already used indoor/Japanese arched ants' images as the training set in our previous work [26] and tested our model on outdoor/black ant images (Seq0010), achieving a tracking accuracy up to 92%. Vice versa, we also conducted experiments using outdoor images as the training set and indoor images as the test set, which are presented in a method manuscript that we are preparing [49], which can be found at arXiv. Hence, it will help biologists to quantify and analyze the foraging patterns of ant colonies in natural environments, such as foraging strategies, partner gathering, and collaborative transportation.

Methods

Hardware devices for acquiring raw data

Indoor environment

For indoor environments, we used a cylindrical container made of transparent plastic providing a space for the ants to move around. This container has a bottom diameter of 10 *cm*, a side height of 15 *cm*, and is not closed at the top. Ants, loaded in the container, were filmed with a high-resolution video camera (Panasonic GX 85) with 25 FPS in the format H.264 with a resolution of 1920×1080 pixels. To ensure stable filming, we fixed the camera on a tripod, as well as hung a light bulb above the container. The height of the camera from the bottom of the container was 30 *cm*, and the filming angles in both the horizontal and vertical directions were 0 °. Also, the camera has an angle of view of 16° and 7.5° in the horizontal and vertical directions, respectively. Figure 5 presents the filming illustration, and the line segment BD denotes the length or width of the filming scene. As a result, we can use the known information to infer the value of line segment BD, as shown in Equation 4. Further, we can easily obtain the area of the indoor scene is 136 cm^2 (17 *cm* × 8 *cm*). Besides, the anti-dusting powder was applied to the inner wall of the container, preventing ants from escaping from the container during the filming.

Outdoor environment

For natural environments, there was no processing for the backgrounds of the scenes. The camera type was mainly the Canon 5d, which has a resolution of 1280 × 720 with a frame rate of 30 FPS. The height of the camera from the ground was 30 *cm*. In different scenes, the horizontal filming angles were different (as shown in Table 1), while the vertical filming angles were all 0°. Additionally, the angle of view of the camera in the horizontal and vertical directions was 16° and 7.5°, respectively. Likewise, according to Figure 5 and Equation 4, we can calculate the area of each outdoor scene, and the concrete values are shown in Table 1.

$$
\begin{aligned}
BD &= AD - AB \\
&= OA \times \tan\angle AOD - OA \times \tan\angle AOB \\
&= OA \times (\tan\angle AOD - \tan\angle AOB) \\
&= OA \times (\tan(\angle AOC + \angle COD) - \tan(\angle AOC - \angle BOC)) \\
&= Height \times (\tan(\angle\alpha + \angle\beta) - \tan(\angle\alpha - \angle\beta))
\end{aligned}
\tag{4}
$$

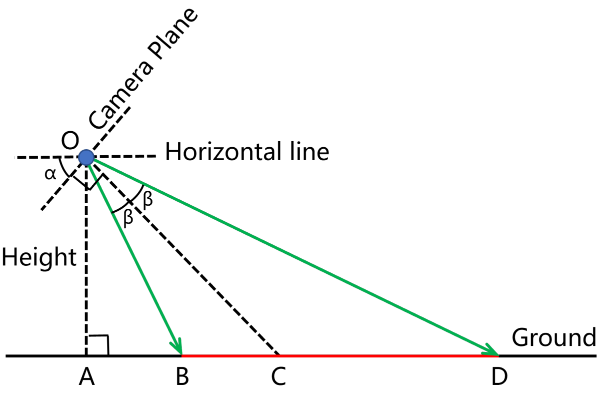

Figure 5. Illustration of the way of camera filming. Take the horizontal direction as an example. The camera is at point O, the height from the ground is OA (denoted by Height), the angle between the filming angle and the horizontal line is α, and the camera's angle of view is β. Thus, according to the position of the angle of view extending to the ground (green arrow line), the horizontal filming range can be determined, denoted by the line BD (red line segment)

Description of the marking software VisualMarkData

We developed an image sequence marking software called VisualMarkData to provide the locations and identification numbers of objects in the sequence for motion analysis. The overall annotation pipeline for the dataset using this software is shown in Figure 1. The operation procedure of VisualMarkData is as follows, and its interface is shown in Figure 6.

- **Choose Image Set.** Before marking, the user should click "Choose ImageSet" to select an image set. The filename of the image set is defined in the format of "SeqXObjectYImageZ", where X is the name of the sequence, Y is the number of objects in the first frame and Z is the size of the bounding box which represents the object. For example, the image set, named "Seq0001Object10Image94", indicates that the sequence "0001" contains 10 objects in the first frame, and each object will be marked with a bounding box with the size of 94x94.
- **Create Output Directory.** The user needs to click "Output Directory" to select the storage path of annotations. Since VisualMarkData only focuses on one object per marking round (each round goes through the whole image sequence), the output folder is suggested to be named with the identification number of the object, e.g. "0001". As the identity number of the object is user-defined, the user can use any number for the object and folder as long as it is unique.
- **Select Start Frame.** In the last step before starting marking, you need to enter the start frame, the default value is 0. This means that you are allowed to exit the software halfway and continue the progress of the current marking task the next time. Then, you can click the "Start" button.
- **Marking.** The user clicks on the center of the object in the current frame, and the software will automatically save the digital location of the center, as well as a bounding box centered on the object. It should be emphasized that the user only marks the same object until finishes the entire image sequence, and then the user can focus on another object by repeating the same operation for the previous one.
- **Next Frame.** The user clicks the "Next" button to show the next frame on the window of the software. The marked location on the previous frame will be displayed with a green-dotted, which can help the user quickly locate the target object.
- **Previous Frame.** If the marked location of the previous frame is incorrect, the user can click the "Previous" button to roll back one frame.

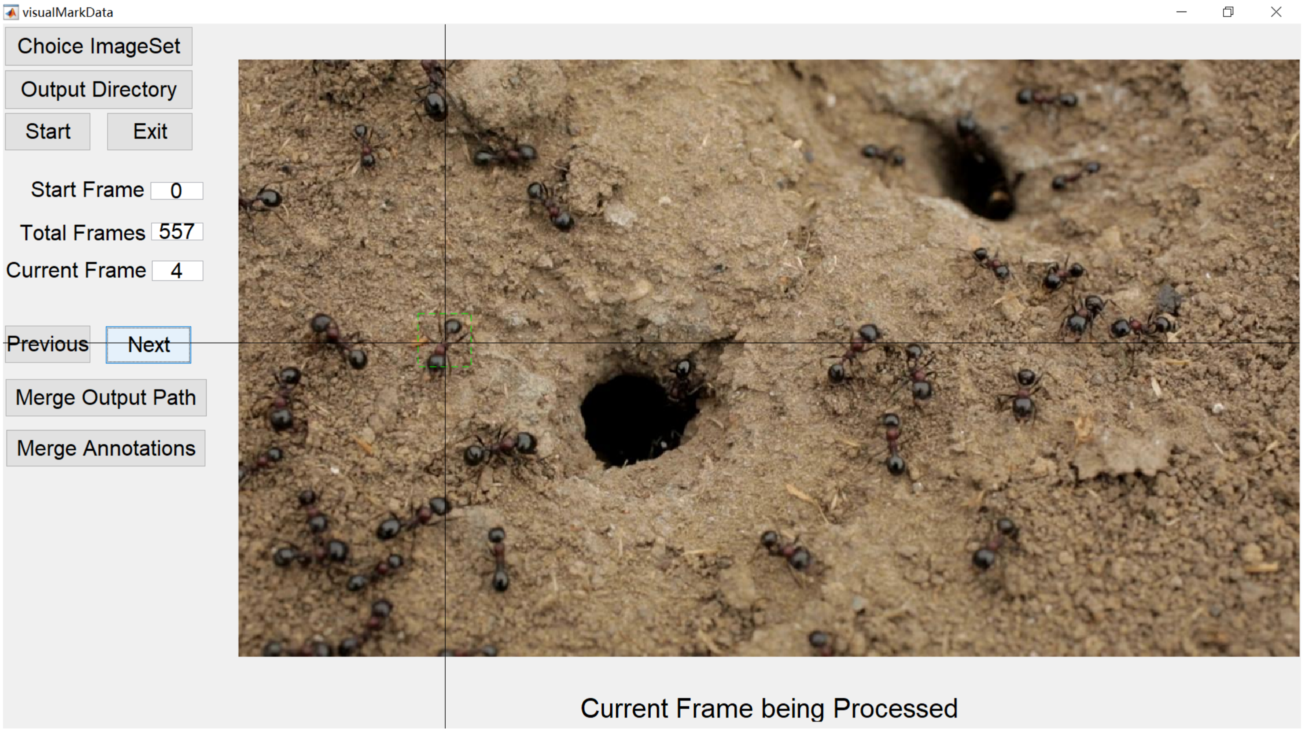

Figure 6. The interactive interface of our image sequence marking software is named VisualMarkData. After selecting an image sequence, the user can acquire the annotation by clicking on the center location of the ant's body.

- **Check and Modify.** After the user finishes marking the entire image set, checking is needed to guarantee the quality. In this case, the user can enter the specific frame to modify the annotations by carrying out **Select Start Frame** step.
- **Merge Annotations.** After all objects in a sequence have been marked and reviewed, the user needs to click the "Merge" button, thereby all annotations for each object will be sorted by frames and then the ID of the object both in ascending order.

Availability of source code and requirements

Lists the following:

- Project name: ANTS_marking_and_analysis_tools
- Project home page: e.g., https://github.com/holmescao/ANTS_marking_and_analysis_tools
- Operating system(s): Platform independent
- Programming language: Python, MATLAB, Shell
- Other requirements: MATLAB R2021b (with Image Processing Toolbox)
- License: MIT License
- RRID: SCR_022543
- biotoolsID identifiers: ants_marking_and_analysis_tools

Data Availability

The dataset supporting the results of this article is available in the ANTS–ant detection and tracking repository at http://dx.doi.org/10.17632/9ws98g4npw.4. Note that the files associated with this dataset are licensed under a Public Domain Dedication license.

Declarations

List of abbreviations

CV: computer vision; ID: identity; FPS: frames per second

Consent for publication

Not applicable

Competing Interests

The authors declare that they have no competing interests.

Funding

This work was supported by the Natural Science Foundation of Fujian Province (No. 2019J01002) and National Nature Science Foundation of China (No.32071057; No. 61673322; No. 31200769), and was partly supported by the Key project of National Key R&D project (No. 2017YFC1703303).

Author's Contributions

M.W. and S.G conceived the experiment(s), X.C. conducted the experiment(s), X.C. analyzed the results. All authors reviewed the manuscript.

Acknowledgements

The authors thank the reviewers, for providing useful suggestions for improvements and valuable feedback on the workflow and the manuscript.

References

1. Vandermeer J, Perfecto I, Philpott SM. Clusters of ant colonies and robust criticality in a tropical agroecosystem. Nature 2008;451(7177):457–459.
2. Balch T, Khan Z, Veloso M. Automatically tracking and analyzing the behavior of live insect colonies. In: Proceedings of the fifth international conference on Autonomous agents; 2001. p. 521–528.
3. Hölldobler B, Wilson EO, et al. The ants. Harvard University Press; 1990.
4. Whitehouse ME, Jaffe K. Ant wars: combat strategies, territory and nest defence in the leaf-cutting antAtta laevigata. Animal Behaviour 1996;51(6):1207–1217.
5. Vaughan RT, Støy K, Sukhatme GS, Matarić MJ. Whistling in the dark: cooperative trail following in uncertain localization space. In: Proceedings of the fourth international conference on Autonomous agents; 2000. p. 187–194.
6. Fewell JH. Social insect networks. Science 2003;301(5641):1867–1870.
7. Motani M, Srinivasan V, Nuggehalli PS. Peoplenet: engineering a wireless virtual social network. In: Proceedings of the 11th annual international conference on Mobile computing and networking; 2005. p. 243–257.
8. Tiacharoen S, Chatchanayuenyong T. Design and development of an intelligent control by using bee colony optimization technique. American Journal of Applied Sciences 2012;9(9):1464.
9. Poff C, Nguyen H, Kang T, Shin MC. Efficient tracking of ants in long video with GPU and interaction. In: 2012 IEEE Workshop on the Applications of Computer Vision (WACV) IEEE; 2012. p. 57–62.
10. Khan Z, Balch T, Dellaert F. MCMC-based particle filtering for tracking a variable number of interacting targets. IEEE transactions on pattern analysis and machine intelligence 2005;27(11):1805–1819.
11. Khan Z, Balch T, Dellaert F. MCMC data association and sparse factorization updating for real time multitarget tracking with merged and multiple measurements. IEEE transactions on pattern analysis and machine intelligence 2006;28(12):1960–1972.
12. Oh SM, Rehg JM, Dellaert F. Parameterized duration mmodeling for switching linear dynamic systems. In: 2006 IEEE Computer Society Conference on Computer Vision and Pattern Recognition (CVPR'06), vol. 2 IEEE; 2006. p. 1694–1700.
13. Veeraraghavan A, Chellappa R, Srinivasan M. Shape-and-behavior encoded tracking of bee dances. IEEE transactions on pattern analysis and machine intelligence 2008;30(3):463–476.
14. Fletcher M, Dornhaus A, Shin MC. Multiple ant tracking with global foreground maximization and variable target proposal distribution. In: 2011 IEEE Workshop on Applications of Computer Vision (WACV) IEEE; 2011. p. 570–576.
15. Li M, Zhang Z, Huang K, Tan T. Estimating the number of people in crowded scenes by mid based foreground segmentation and head-shoulder detection. In: 2008 19th international conference on pattern recognition IEEE; 2008. p. 1–4.
16. Li Y, Huang C, Nevatia R. Learning to associate: Hybridboosted multi-target tracker for crowded scene. In: 2009 IEEE conference on computer vision and pattern recognition IEEE; 2009. p. 2953–2960.
17. Zhao M, Liu H, Wan Y. An improved Canny edge detection algorithm based on DCT. In: 2015 IEEE International Conference on Progress in Informatics and Computing (PIC) IEEE; 2015. p. 234–237.
18. Schmelzer E, Kastberger G. 'Special agents' trigger social waves in giant honeybees (Apis dorsata). Naturwissenschaften 2009;96(12):1431–1441.
19. Kastberger G, Weihmann F, Hoetzl T. Social waves in giant honeybees (Apis dorsata) elicit nest vibrations. Naturwissenschaften 2013;100(7):595–609.
20. Tan K, Dong S, Li X, Liu X, Wang C, Li J, et al. Honey bee inhibitory signaling is tuned to threat severity and can act as a colony alarm signal. PLoS biology 2016;14(3):e1002423.
21. Dong S, Wen P, Zhang Q, Wang Y, Cheng Y, Tan K, et al. Olfactory eavesdropping of predator alarm pheromone by sympatric but not allopatric prey. Animal Behaviour 2018;141:115–125.
22. Schor N, Bechar A, Ignat T, Dombrovsky A, Elad Y, Berman S. Robotic disease detection in greenhouses: Combined detection of powdery mildew and tomato spotted wilt virus. IEEE Robotics and Automation Letters 2016;1(1):354–360.
23. Wang G, Li W, Zuluaga MA, Pratt R, Patel PA, Aertsen M, et al. Interactive medical image segmentation using deep learning with image-specific fine tuning. IEEE transactions on medical imaging 2018;37(7):1562–1573.
24. Wang C. Research and application of traffic sign detection and recognition based on deep learning. In: 2018 International Conference on Robots & Intelligent System (ICRIS) IEEE; 2018. p. 150–152.
25. Imirzian N, Zhang Y, Kurze C, Loreto RG, Chen DZ, Hughes DP. Automated tracking and analysis of ant trajectories shows variation in forager exploration. Scientific reports 2019;9(1):1–10.
26. Cao X, Guo S, Lin J, Zhang W, Liao M. Online tracking of ants based on deep association metrics: method, dataset and evaluation. Pattern Recognition 2020;103:107233.
27. Sabattini J, Reta J, Bugnon L, Cerrudo J, Sabattini R, Peñalva A, et al. AntVideoRecord: Autonomous system to capture the locomotor activity of leafcutter ants. HardwareX 2022;11:e00270.
28. Yuen J, Russell B, Liu C, Torralba A. Labelme video: Building a video database with human annotations. In: 2009 IEEE 12th International Conference on Computer Vision IEEE; 2009. p. 1451–1458.
29. Vondrick C, Patterson D, Ramanan D. Efficiently scaling up crowdsourced video annotation. International journal of computer vision 2013;101(1):184–204.
30. Doermann D, Mihalcik D. Tools and techniques for video performance evaluation. In: Proceedings 15th International Conference on Pattern Recognition. ICPR-2000, vol. 4 IEEE; 2000. p. 167–170.
31. Biresaw TA, Nawaz T, Ferryman J, Dell AI. Vitbat: Video tracking and behavior annotation tool. In: 2016 13th IEEE International Conference on Advanced Video and Signal Based Surveillance (AVSS) IEEE; 2016. p. 295–301.
32. Nakanishi A, Nishino H, Watanabe H, Yokohari F, Nishikawa M. Sex-specific antennal sensory system in the ant Camponotus japonicus: structure and distribution of sensilla on the flagellum. Cell and tissue research 2009;338(1):79–97.
33. He H, Chen Y, Zhang Y, Wei C. Bacteria associated with gut lumen of Camponotus japonicus Mayr. Environmental Entomology 2011;40(6):1405–1409.
34. Nishikawa M, Watanabe H, Yokohari F. Higher brain centers for social tasks in worker ants, Camponotus japonicus. Journal of Comparative Neurology 2012;520(7):1584–1598.
35. Terayama M, Ogata K. Two new species of the ant genus Probolomyrmex (Hymenoptera, Formicidae) from Japan. Kontyu 1988;56(3):590–594.
36. Wang L, Chen J. Fatty amines from little black ants, Monomorium minimum, and their biological activities against red imported fire ants, Solenopsis invicta. Journal of chemical ecology 2015;41(8):708–715.
37. Thompson C. Ants that have pest status in the United States. In: Applied Myrmecology CRC press; 2019.p. 51–67.
38. Sanders C. The Biology of Carpenter Ants in New Brunswick1. The Canadian Entomologist 1964;96(6):894–909.
39. Carney WP. Behavioral and morphological changes in carpenter ants harboring dicrocoeliid metacercariae. The American

Midland Naturalist 1969;82(2):605–611.

40. Carlin NF, Hölldobler B. The kin recognition system of carpenter ants (Camponotus spp.). Behavioral Ecology and Sociobiology 1986;19(2):123–134.
41. Ayieko MA, Kinyuru J, Ndong'a M, Kenji G. Nutritional value and consumption of black ants (Carebara vidua Smith) from the Lake Victoria region in Kenya. Advance Journal of Food Science and Technology 2012;.
42. Leal-Taixé L, Milan A, Reid I, Roth S, Schindler K. Motchallenge 2015: Towards a benchmark for multi-target tracking. arXiv preprint arXiv:150401942 2015;.
43. Fabbri R, Costa LDF, Torelli JC, Bruno OM. 2D Euclidean distance transform algorithms: A comparative survey. ACM Computing Surveys (CSUR) 2008;40(1):1–44.
44. Wang Q, Song W, Zhang J, Lo S. Bi-directional movement characteristics of Camponotus japonicus ants during nest relocation. Journal of Experimental Biology 2018;221(18):jeb181669.
45. Bond AB. Optimal foraging in a uniform habitat: the search mechanism of the green lacewing. Animal Behaviour 1980;28(1):10–19.
46. Deffernez L, Champagne P, Verhaeghe JC, Josens G, Loreau M. Analysis of the spatio-temporal niche of foraging grassland ants in the field. Insectes sociaux 1990;37(1):1–13.
47. Feener DH, Moss KA. Defense against parasites by hitchhikers in leaf-cutting ants: a quantitative assessment. Behavioral ecology and sociobiology 1990;26(1):17–29.
48. Loreto RG, Hart AG, Pereira TM, Freitas ML, Hughes DP, Elliot SL. Foraging ants trade off further for faster: use of natural bridges and trunk trail permanency in carpenter ants. Naturwissenschaften 2013;100(10):957–963.
49. Wu M, Cao X, Guo S. Swarm behavior tracking based on a deep vision algorithm. arXiv preprint arXiv:220403319 2022;.

revised manuscript with track changes

Click here to access/download;Manuscript;revised manuscript with track changes.pdf

OXFORD (GIGA)^n SCIENCE

GigaScience, 2017, 1–9

doi: xx.xxxx/xxxx
Manuscript in Preparation
Paper

PAPER

A dataset of ant colonies motion trajectories in indoor and outdoor scenes for social cluster behavior study

Meihong Wu[1,†], Xiaoyan Cao[1,†], Ming Yang[1], Xiaoyu Cao[2] and Shihui Guo[1,*]

[1]School of Informatics, Xiamen University, Xiamen, 361000, China and [2]Chemistry and Chemical Engineering, Xiamen University, Xiamen, 361000, China

*guoshihui@xmu.edu.cn
†Contributed equally.

Abstract

Motion and interaction of social insects (such as ants) have been studied by many researchers to understand the clustering mechanism. Most studies in the field of ant behavior have only focused on indoor environments (laboratory setup), while outdoor environments (natural environments) are still underexplored. In this paper, for the first time, we collect 10 videos and 3 species of ant colonies from different ~~indoor and~~ scenes, including 5 indoor and 5 outdoor scenes. And we develop an image sequence marking software named VisualMarkData, which enables us to provide annotations of ants in the video: (1) offers a comprehensive annotation of states at individual-target as well as colony-target level; (2) provides a simple matrix format to represent multiple targets and multiple groups of annotations (along with their IDs and behavior labels); (3) during the annotation process, we propose a simple and effective visualization that takes the annotation information of the previous frame as a reference and then simply clicks on the center point of each target to complete the annotation; and (4) we develop a user-friendly windows-based GUI to minimize labor and maximize annotation quality. In all ~~5354~~ 5,354 frames, the location information and the identification number of each ant are recorded for a total of 712 ants and ~~114112~~ 114,112 annotations. Moreover, we provide visual analysis tools to assess and validate the technical quality and reproducibility of our data. It is hoped that this dataset will contribute to a deeper exploration on the behavior of the ant colony.

Key words: social insects; outdoor scenes; image sequence marking software

~~Background~~Context

Social insects often tend to cluster into a colony [1], which forms a complex social network [2]. From time to time, the social network springs up with self-organized clustering behaviors, including division of labor [3], task specialization [4], and distributed problem solving [5]. Biologists analyze the evolution of social network to understand the clustering behavior of insects [6], thus promoting the development of relevant modern applications, such as wireless communication [7] and cluster intelligent control [8]. The key requirement of this research is the ability to track the motions and interactions of each individual robustly and accurately.

Until the late 20th century, biologists still manually ~~marked~~ tracked the motion trajectories on the video to guarantee the ~~quality~~accuracy of the marking. However, they have to track each individual at one time, which might mean watching the entire video 50 times or more in a crowded scene~~. [9]Obviously, manually~~ [9]. Manually tracking is time-consuming and prone to human error. It becomes an inhibiting factor in obtaining the complete and accurate dataset required to analyze the evolution of social networks. Therefore, in the past two decades, attempts have been made to automate the tracking process for social insects utilizing computer vision (CV) techniques [10, 11, 12, 13, 14].

Traditional CV techniques ~~free~~ release researchers from manual work through approaches such as foreground segmentation algorithm [15], temporal difference method [10] and hungarian algorithm [16]. Such approaches, however, have failed to address the noise in the image [17], resulting in the limitation that a laboratory environment with a clean background is needed. Nevertheless, many scientifically valuable results are obtained in nature rather

Compiled on: July 27, 2022.
Draft manuscript prepared by the author.

than laboratory environment [18, 19, 20, 21].

Fortunately, with the emergence of deep learning, CV techniques are already capable of addressing many complex tasks [22, 23, 24], which brings a piece of good news to automated insect tracking in outdoor scenes. Several studies have explored automated multi-ant tracking in outdoor scenes using deep learning-based models [25, 26]. Experimental results demonstrate that these models could be scaled up into a cost-effective alternative to traditional manual tracking methods which are typically costly and/or labor intensive [25, 26]. A critical requirement for the development of these models is access to the datasets containing annotations of motion trajectories of insects in the video. Several works are working on improving the capture of such insects in natural environments [25, 27]. To the best of our knowledge, however, ~~the current studies are all using only one outdoor-scene dataset~~only a few works [25, 26] annotate motion trajectories in videos, and both use only a single outdoor scene sequence, which lacks data diversity.

Considering the importance of annotating targets in videos, some annotation tools have been proposed over the years including LabelME [28], VATIC [29], ViPER [30], and ViTBAT [31]. Except for ViTBAT, other tools are generally more suitable for annotating ground-truth information at the individual target level in terms of tracking targets. As for ViTBAT supports annotating a group of targets but requires much effort to set up rectangular boxes with different sizes for each target. Additionally, it will not display the annotation results of the previous frame in the current frame, which makes it difficult for the user to identify the same target during the annotation process of the video sequence. Moreover, it only supports Linux systems, which is ~~lack of data diversity~~difficult to use for non-computer background biology researchers. In our opinion, as a marking tool, it is desired to be user-friendly, minimize human effort and maximize annotation.

To summarise, the contributions of our work are mainly in the aspects of tool and dataset.

~~In this paper, we collect video recordings of 10 ant colonies from different scenes (including~~ With respect to the tool, we propose the VisualMarkData, which allows users to generate ground-truth information of multi-target motion trajectories in video sequences. Specifically, VisualMarkData offers: (1) a comprehensive annotation of states at individual-target as well as group-target level; (2) representation of annotations (together with their IDs and behavior labels) of multiple targets and multiple groups in a simple-to-access matrix format; (3) a simple and efficient visualization during annotation, which presents the annotation information of the previous frame as a reference and then only requires clicking on the center point of each target to complete the annotation; and (4) a windows-based friendly graphical user interface that minimizes labor and maximizes annotation quality.

With regard to the dataset, we are the first to construct the ant colony activity dataset with annotations with multiple species and colonies in both indoor and outdoor ~~scenes). Besides, we develop an image sequence mark software named VisualMarkData, which is used to mark the pixel patches covered by ants in each frame of the video~~environments. Concretely, we build equipment for video acquisition in various environments and obtain a number of different ant colony activity videos, including 3 species and 10 colonies. Then utilizing VisualMarkData and following the process shown in Figure 1, a large-scale dataset of ant colony activity with annotations is constructed. The total size of ~~our dataset is 5354~~ the dataset is 5,354 frames, 712 ants, and ~~114112~~ 114,112 labels. We believe that ~~our~~ the dataset will benefit future research on social insect behavior analysis.

Data Description

We collect 10 videos that record activities of different ant colonies, including colonies from both indoor and outdoor scenes. To help us mark the motion trajectories, we develop an image sequence marking software called VisualMarkData.

After spending a large quantity of time and effort, we obtain a dataset with ~~5354 frames and 114112~~ 5,354 frames and 114,112 annotations. Table 1 ~~shows the statistical information of our dataset~~ describes the dataset in detail.

Data acquisition

Indoor environment

~~We collect~~ Japanese arched ants (also called Camponotus japonicus ants) are widely studied by behavioral ecologists and social biologists [32, 33, 34]. And these ants are often domesticated, thus they are suitable for observation in laboratory environments. We collected 50 workers of Japanese arched ~~ants~~ ant species, which ranged from 7.4 to 13.8 *mm* in body length [35]. We ~~construct~~ constructed a laboratory environment for them, including a stable light source, stable temperature and a transparent plastic container. ~~We randomly divide~~ And the background of the container was clean and without the nest. We randomly divided them into 5 colonies of ~~10~~ ants each. Then, we ~~load~~ loaded each colony into the container in turns and ~~film~~ filmed their activities with a high-resolution video camera. These videos were named Seq0001 to Seq0005. These filmings took place on April 15, 2019, in the morning in Xiamen, Fujian, China. More detailed information is provided in Table 1.

Outdoor environment

~~We collect 5 videos of black~~ Little black ants [36, 37] and Carpenter ants [38, 39, 40], are the focus of research by behavioral ecologists and sociobiologists. We acquired five videos from five ant colonies in different outdoor environments, ~~with the number of black ants each colony~~ ranging from 73 to 193 ~~in the videos. The body length of black ants is~~ workers. The species of these ant colonies were Carpenter and Little black ants, and their body length was between 8 ~~to~~ and 10 ~~*mm*~~ mm [41]. ~~These videos are provided by Depositphotos, an online video site (), and access is subject to a royalty-free license.~~ We named the obtained videos Seq0006 to Seq0010. Concrete and uneven stones were in the background of Seq0006. Seq0007 and Seq0008 were filmed in the dry grass. As for Seq0009 and Seq0010, they were filmed on a dirt road and a rocky road, respectively. And the backgrounds of scenes were without any processing. Except for Seq0010, the scenes of the other four videos were taken at the entrance of the nest. More informative details about the time, location, and temperature of each film are shown in Table 1.

Data Records

The dataset consists of 10 image sequences from different scenes in JPEG digital image format, which is publicly available on https://data.mendeley.com/datasets/9ws98g4npw/3. Alongside, we provide annotations marked by VisualMarkData for all image sequences in the form of text. In the dataset, the images and annotations of each sequence are organized into three folders are named 'det', 'gt', and 'img'.

Det folder

~~The 'det' folder contains a 'det.txt' file which is the ground truth for detection, recording the~~

In the same format as the dataset of multi-object tracking challenge [42], we record information such as the identity and lo-

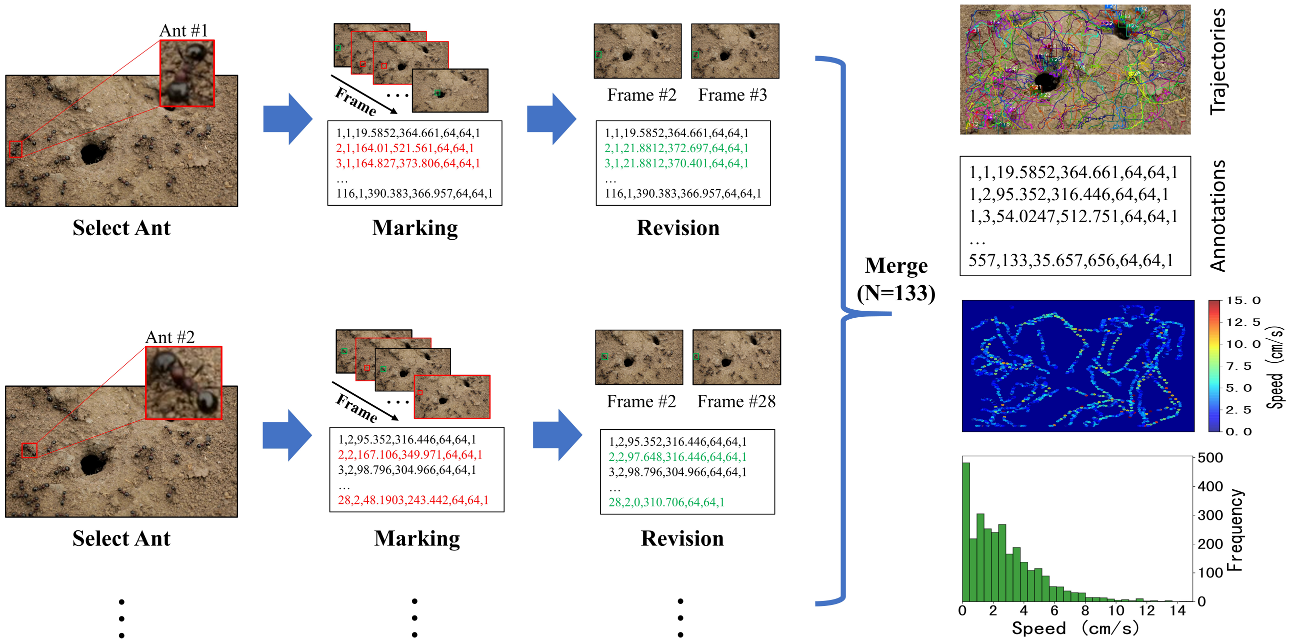

Figure 1. The pipeline for marking motion trajectories of ants in an image sequence, taking an outdoor scene as an example. A total of 133 ants appear in this image sequence, and we select one ant to be marked in each epoch. We use a square bounding box to point out the ant's location and record the relevant parameters at the same time. After all ants of the entire image sequence have been marked, we check the quality of the annotations frame by frame so that wrong annotations (red font) can be corrected (green font). Then, we merge all the annotations of the image sequence into one file. Additionally, ~~we provide~~ three Python scripts are provided to generate three visualization ~~tools~~ results to verify the ~~data~~ quality of the data, including the trajectories drawn on the original graph, the heat map of motion speed, and the histogram of the frequency distribution of motion speed.

Filming details

Scene	Sequence	Angle	Height	Temp	Datetime	Location	Camera
Indoor	Seq0001	0°	30*cm*	24°C-26°C	2019/04/15 morning	Xiamen, Fujian, China	Panasonic GX 85
	Seq0002	0°	30*cm*	24°C-26°C	2019/04/15 morning	Xiamen, Fujian, China	Panasonic GX 85
	Seq0003	0°	30*cm*	24°C-26°C	2019/04/15 morning	Xiamen, Fujian, China	Panasonic GX 85
	Seq0004	0°	30*cm*	24°C-26°C	2019/04/15 morning	Xiamen, Fujian, China	Panasonic GX 85
	Seq0005	0°	30*cm*	24°C-26°C	2019/04/15 morning	Xiamen, Fujian, China	Panasonic GX 85
Outdoor	Seq0006	45°	30*cm*	15°C-18°C	2019/06/23 morning	Russian Federation, Saint-Petersburg	Canon 5d
	Seq0007	30°	30*cm*	30°C-35°C	2019/07/21 morning	Greece, Athens	Canon 5d
	Seq0008	30°	30*cm*	15°C-18°C	2019/06/23 morning	Russian Federation, Saint-Petersburg	Canon 5d
	Seq0009	30°	30*cm*	15°C-18°C	2019/06/23 morning	Russian Federation, Saint-Petersburg	Canon 5d
	Seq0010	0°	30*cm*	15°C-17°C	2019/04/21 morning	United States, Neptune Beach	Canon T3i

Description of videos with annotations

Scene	Sequence	FPS	Resolution	Length	Ants	Annotations	Species	Entrance	Area
Indoor	Seq0001	25	1920×1080	351 (00:14)	10	3510	Japanese arched ants	no	17*cm*×8*cm*
	Seq0002			351 (00:14)	10	3510	Japanese arched ants	no	17*cm*×8*cm*
	Seq0003			351 (00:14)	10	3510	Japanese arched ants	no	17*cm*×8*cm*
	Seq0004			351 (00:14)	10	3510	Japanese arched ants	no	17*cm*×8*cm*
	Seq0005			1001 (00:40)	10	3510	Japanese arched ants	no	17*cm*×8*cm*
Outdoor	Seq0006	30	1280×720	600 (00:20)	73	11178	Carpenter ants	yes	17*cm*×16*cm*
	Seq0007			677 (00:23)	162	25158	Little black ants	yes	17*cm*×11*cm*
	Seq0008			577 (00:19)	133	10280	Carpenter ants	yes	17*cm*×11*cm*
	Seq0009			526 (00:18)	193	27902	Carpenter ants	yes	17*cm*×11*cm*
	Seq0010			569 (00:19)	101	22044	Little black ants	no	17*cm*×8*cm*

Table 1. Description of ant videos with annotations in indoor and outdoor scenes. **Top part provides filming details**. Sequence = Name of video for each colony. Angle = The horizontal angle of the camera during filming. Height = Height of camera from the ground. Temp = Local temperature during filming. Datetime = Date and time of the filming. Location = Location of the filming. Camera = Camera type. **Bottom part provides a description of ant videos with annotations.** FPS = Frame rate of the video. Resolution = The resolution of the video. Length = The number of frames of the video, with the duration in parentheses. Ants = The number of ants with different IDs that appear in the video. Annotations = The number of ants instances labeled in the video. Species = The ant species. Entrance = Whether the colony is active at the nest entrance. Area = The area of the filmed scene. Note that the camera's angle of view is 16° and 7.5° in the horizontal and vertical directions, respectively, which are not represented in the table.

Position	Name	Description
1	Frame number	Indicate at which frame the object is present
2	Identity number	Each ant trajectory is identified by a unique ID (-1 for detections)
3	Bounding box left	Coordinate of the top-left corner of the ant bounding box
4	Bounding box top	Coordinate of the top-left corner of the ant bounding box
5	Bounding box width	Width in pixels of the ant bounding box
6	Bounding box height	Height in pixels of the ant bounding box
7	Confidence score	Indicates how confident the detector is that this instance is a ant.<br>For the ground truth and results, it acts as a flag whether the entry is to be considered

Table 2. Data format for annotation files, both for 'det.txt' and 'gt.txt' files.

cation parameters of ~~the ants in all frames, which is similar to multi-object tracking challenge [42].Each~~ all ants in each frame for detection. Such information is stored in a 'det.txt' file in a folder named 'det' in our dataset. Concretely, each line represents one ant instance, and it contains 7 values (also called attributes) as shown in Table 2. The first number indicates in which frame the ant appears (sorted by ascending order), while the second number identifies that ant as belonging to a trajectory by assigning a unique ID (set to -1 in a detection file, as no ID is assigned yet). The next four numbers indicate the location of the bounding box of the ant in 2D image coordinates. The location is indicated by the top-left corner as well as the width and height of the bounding box. This is followed by a single number, which denotes the confidence score.

Gt folder

~~The 'gt' folder contains~~

In our dataset, we provide ground-truth records for multi-object tracking. These information are stored in a 'gt.txt' file ~~, which is the ground truth for muti-object tracking~~in a folder named 'gt'. Similar to the previous description of the 'det.txt' file, ~~it~~ the records of each instance in the 'gt.txt' file also contains 7 values (~~see details in~~ also called attributes), see Table 2 ~~)~~for details. The difference compared to the 'det.txt' file is that the second number in the 'gt.txt' file records the ID of an ant as belonging to a trajectory, which provides the key information for implementing multi-ant tracking. Besides, each ant can be assigned to only one trajectory.

Img folder

~~The 'img' folder stores~~

In our dataset, we provide the original image sequence converted from the video~~.~~, which are stored in the 'img' folder. All images are converted to JPEG and named sequentially to a 6-digit file name (e.g., 000001.jpg).

~~Analyses~~Data Validation and quality control

Visually ~~confirm~~confirmation

~~The ground truth~~ In all 10 videos, we have 2 staff to mark indoor videos and 3 staff to mark outdoor videos. Furthermore, the ground-truth annotations for all image sequences in the dataset ~~are~~ were visually confirmed by ~~the data annotation~~ one staff. The visual reviewing consists of two aspects, sequence-level (coarse-grained) and image-level (fine-grained).

Firstly, ~~staffs perform~~ the staff performed a coarse-grained review of a single sequence. Specifically, we ~~draw~~ drew the annotations on the corresponding images, and then ~~convert~~ converted the image sequence to video. For each scene, an example image frame is shown in Figure 2 (a) and Figure 3 (a). By replaying the video, staff can quickly confirm which segments of the video are of poor quality and needed to be re-marked. ~~For each scene, an example image frame is shown in Figure 2~~ Figure 4 (a) ~~and Figure 3 (a).~~ shows an example of a segment distinguished as low-quality annotations. The sequence-level checking time consumption per video is 8 to 10 times the original video sequence duration, which depends on the number of ants in the video. After that, staff ~~reviews~~ reviewed the quality of annotations frame-by-frame via VisualMarkData. For inaccurate annotations, staff ~~modifies~~ modified manually by using the "Check and modify" function of VisualMarkData (see details in Methods). Figure 4 (b) shows the modified annotations. The image-level checking speed is about 0.5 sec per ant instance while correction takes about 2 sec per ant instance.

Motion speed analysis

Further, to demonstrate the reliability of our dataset, we ~~analyze~~ analyzed the distribution of the movement speed of the ants in our dataset. First, for each ant, we ~~use~~ used the 2D Euclidean distance [43] to calculate its pixel distance between two adjacent frames. Therefore, the pixel distance $\triangle ps_t$ of the ant at frame t can be defined by the following equation:

$$\triangle ps_t = \sqrt{(px_t - px_{t-1})^2 + (py_t - py_{t-1})^2} \quad (1)$$

where px_t denotes the pixel position of the ant in the horizontal direction at frame t. Similarly, py_t denotes the pixel position in the vertical direction. To convert the pixel distance to real-world coordinates, we ~~divide~~ divided the ant's body length L (unit: m) in the real world by body length n (unit: $pixel$) in the image. Thus, the real-world displacement of the ant at frame t, $\triangle s_t$ (unit: m) can be expressed as follows:

$$\triangle s_t = \triangle ps_t \times L/n \quad (2)$$

Since the FPS for a specifc video is a constant f_c, the ~~velocity~~ speed v_t (unit: $m \cdot s^{-1}$) at frame t can be formulated as:

$$v_t = \frac{\triangle s_t}{1/f_c} \quad (3)$$

Where, the v_0 is set to ~~0.~~

0, i.e. we assumed that the ants were stationary at the initial moment. According to the aforementioned equations, combined with the location information of ants in annotations, we can analyze the motion speed of ants in the video, as shown in Figure 2 (b), (c) and Figure 3 (b), (c). Specifically, the overall motion speed of ants in indoor and outdoor scenes are 2.16±1.49 $cm \cdot s^{-1}$ and 1.98±1.84 $cm \cdot s^{-1}$, respectively. These values are within a reasonable range (ants average motion speed is 2.85 $cm \cdot s^{-1}$ under bi-directional traffic condition [44]). This demonstrates that the ant colony activity dataset we collected and marked is real and reliable.

Discussion

The image sequence marking software VisualMarkData is a toolkit with interactive visualization. The goal of the software is to provide a convenient tool for researchers marking movement trajectories of social insects in videos, thus facilitating the study of the behavioral mechanisms of social insects. Additionally, by using the software,

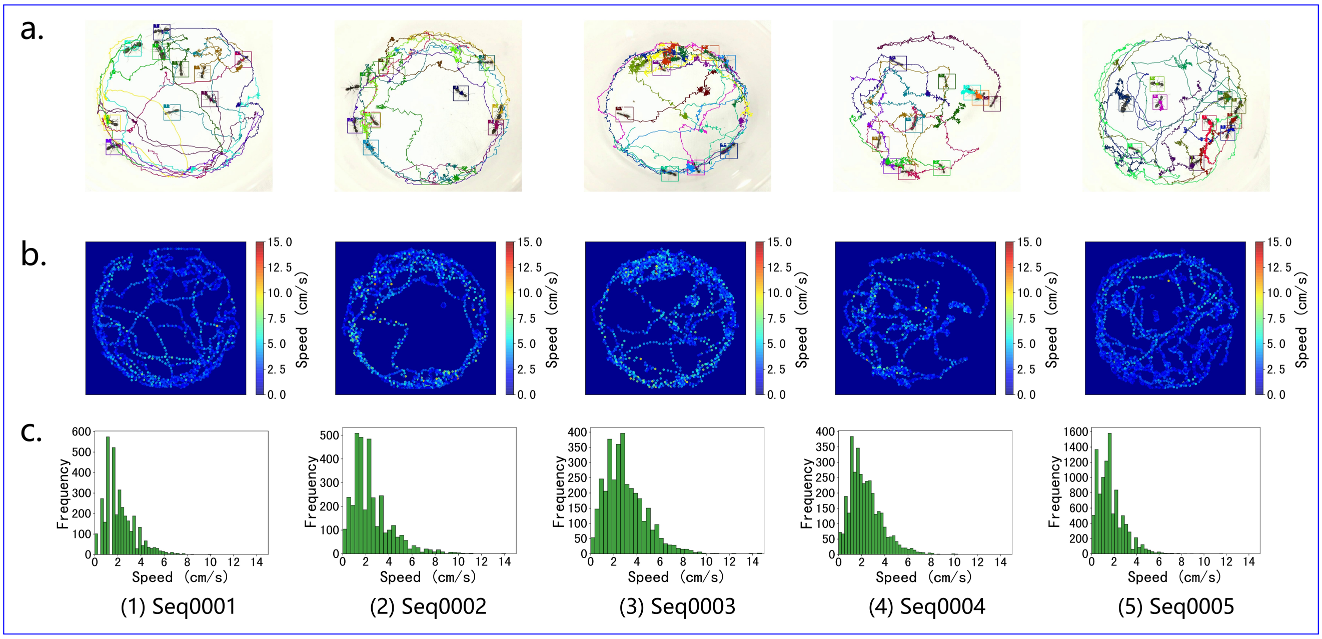

Figure 2. Visual analysis of the marking results on indoor ant videos. (a) Visualization of motion trajectories of the ants for each sequence of the indoor scene. (b) Speed distributions in image space for ~~5~~ five sequences of indoor scenes~~, respectively~~. (c) ~~Ant speed histograms per~~ Histogram of the frequency of ant speeds in *cm/s* for indoor ~~sequence~~sequences.

researchers will obtain standardized annotation data, as details in the previous section. VisualMarkData is open source, which enables researchers to mark their image sequence datasets of any multi-object motion scenario. Alongside, we have provided publicly available Python Scripts at https://github.com/holmescao/ANTS_marking_and_analysis_tools, to illustrate the analysis of data as well as usage of the data. To visualize and reproduce the results described in the Technical Validation section, we develop two scripts for the researchers. Also, we provide another script to calculate metrics [42] of multi-object tracking that enables any deep learning algorithm to evaluate the tracking accuracy on ~~our~~ the dataset. The annotated trajectory data can be used for training and testing of supervised learning models, thus providing a powerful tool for studying a wider range of ant colony behaviors.

In the future, ~~we will enrich the VisualMarkData with more features~~ it is possible that VisualMarkData software will be made some updates to reduce the difficulty of marking and improve the efficiency of marking. The software currently marks targets based on their center points, and we are considering introducing stretchable annotation capabilities based on rectangles or ellipses. In addition, the simultaneous annotation of multiple targets in one frame is also a feature worth developing. Along with that, we can introduce semi-automated annotation, i.e., embedding a neural network model into the VisualMarkData, which will automatically predict and annotate objects of the current frame based on the information in the previous frame. Thus, the annotators only need to fine-tune the annotation, which will significantly improve the efficiency of the annotation.

The dataset and VisualMarkData will boost researchers both in biology and computer science to study on behavior of social insects in different environments. We hope that this work will contribute to the potential discovery of ant colony behavioral mechanisms and facilitate the application of the image processing field in biology.

Potential ~~implications~~usage of dataset

~~Swarm~~ Swarming behavior is one of the most important features of social insects~~, which has important significance for the study of embodied intelligence [8]. Specifically, social insects often tend to cluster into a colony~~ [1]~~, which forms a complex dynamical system together with the surrounding environment [2]. So far, researchers do not know enough about the mechanisms behind swarm behaviors of social insects. We believe our image sequence marking software and dataset could facilitate the analysis of ant colony behavior leading to the development of embodied intelligence~~ [1], often involving division of labor [3], task specialization [4], distributed problem solving [5], etc. To reveal the mechanisms behind swarming behavior requires observing insect colonies over long periods of time as well as recording the motion trajectory of each individual [9]. Before the advance of computer vision technology, biologists utilize manual tracking to study insect behaviors [45, 46]. Since manual recording is time-consuming and laborious, biologists focus only on individual behavioral studies, including foraging activity [46] and prey avoidance [45], etc. In recent years, in order to enable rapid tracking of the activities of multiple insects simultaneously, automated image-based tracking techniques are employed, and a lot of attempts are made to improve the accuracy of tracking [10, 11, 12, 13, 14]. These techniques have assisted biologists to discover some colony mechanisms. For example, Balch T et al [2] found that a number of ants would interact at the entrance of the nest when some find food nearby. However, current studies are almost limited to laboratory settings with clean backgrounds. Such approaches disregard the influence of the environment surrounding the insect colony, including potential predators [47], obstacles on the road [48], etc. In contrast, we provide labeled motion trajectories of ant colonies active outdoors, containing a variety of scenes. These data can be used to train deep learning models for automated tracking ants in natural environments. Moreover, we already used indoor/Japanese arched ants' images as the training set in our previous work [26] and tested our model on outdoor/black ant images (Seq0010), achieving a tracking accuracy up to 92%. Vice versa, we also conducted experiments using outdoor images as the training set and indoor images as the test set, which are presented in a method manuscript that we are preparing [49], which can be found at arXiv. Hence, it will help biologists to quantify and analyze the foraging patterns of ant colonies in natural environments, such as foraging strategies, partner gathering, and collaborative transportation.

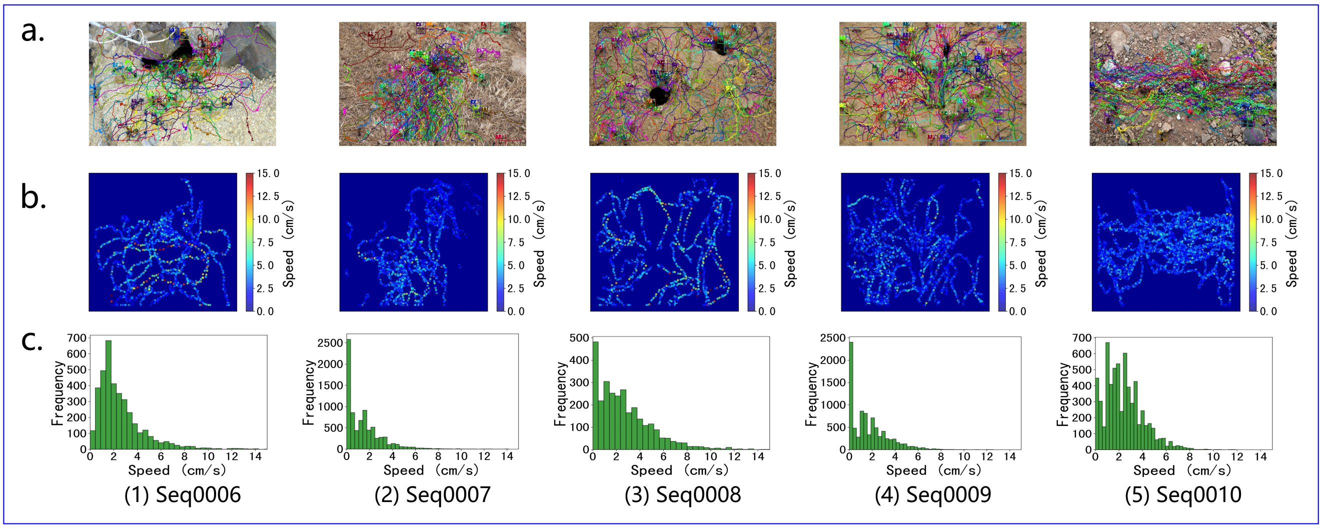

Figure 3. Visual analysis of the marking results on outdoor ant videos. (a) Visualization of motion trajectories of the ants for each sequence of the outdoor scene. (b) Speed distributions in image space for ~~5~~ five consecutive sequences of ~~outdoor~~ indoor scenes~~, respectively~~. (c) ~~Ant speed histograms per outdoor sequence~~Histogram of the frequency of ant speeds in cm/s for indoor sequences.

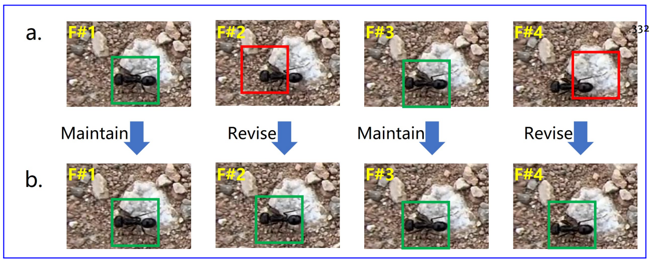

Figure 4. An example of re-marking a segment. (a) indicates the result of marking before revision, where the green and red boxes indicate the high and low quality annotations, respectively. (b) indicates the result after re-marking, where we only need to revise the low quality annotations in (a) to get the high quality annotations (green box).

Methods

Hardware devices for acquiring raw data

Indoor environment

For indoor environments, we ~~use~~ used a cylindrical container made of transparent plastic providing a space for the ants to move around. This container has a bottom diameter of 10 *cm*, a side height of 15 *cm*, and is not closed at the top. Ants, loaded in the container, ~~are~~ were filmed with a high-resolution video camera (Panasonic GX 85) with 25 ~~frames per second (FPS)~~ FPS in the format H.264 with a resolution of 1920×1080 pixels. ~~And the distance between the camera and the top~~ To ensure stable filming, we fixed the camera on a tripod, as well as hung a light bulb above the container. The height of the camera from the bottom of the container ~~is~~ was 30 *cm*~~so that the filming view of the camera can cover the whole container. To ensure stable filming , we fix the camera on a tripod, as well as hanging a light bulb above the container~~, and the filming angles in both the horizontal and vertical directions were 0 °. Also, the camera has an angle of view of 16° and 7.5° in the horizontal and vertical directions, respectively. Figure 5 presents the filming illustration, and the line segment BD denotes the length or width of the filming scene. As a result, we can use the known information to infer the value of line segment BD, as shown in Equation 4. Further, we can easily obtain the area of the indoor scene is 136 cm^2 (17 cm× 8 cm). Besides, the anti-dusting powder ~~is~~ was applied to the inner wall of the container, preventing ants from escaping from the container during the filming.

Outdoor environment

For natural environments, there was no processing for the backgrounds of the scenes. The camera type was mainly the Canon 5d, which has a resolution of 1280 × 720 with a frame rate of 30 FPS. The height of the camera from the ground was 30 *cm*. In different scenes, the horizontal filming angles were different (as shown in Table 1), while the vertical filming angles were all 0°. Additionally, the angle of view of the camera in the horizontal and vertical directions was 16° and 7.5°, respectively. Likewise, according to Figure 5 and Equation 4, we can calculate the area of each outdoor scene, and the concrete values are shown in Table 1.

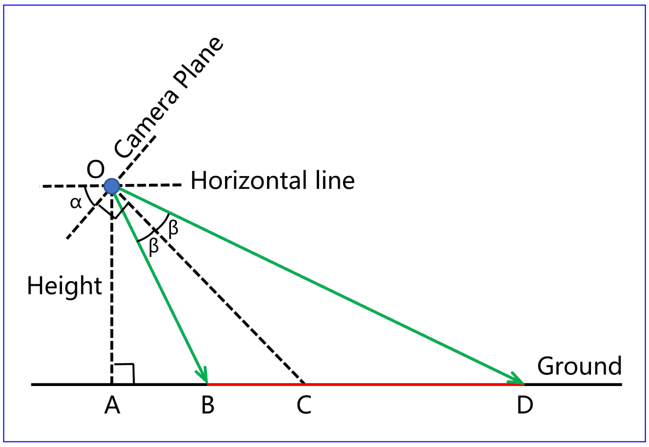

Figure 5. Illustration of the way of camera filming. Take the horizontal direction as an example. The camera is at point O, the height from the ground is OA (denoted by Height), the angle between the filming angle and the horizontal line is α, and the camera's angle of view is β. Thus, according to the position of the angle of view extending to the ground (green arrow line), the horizontal filming range can be determined, denoted by the line BD (red line segment)

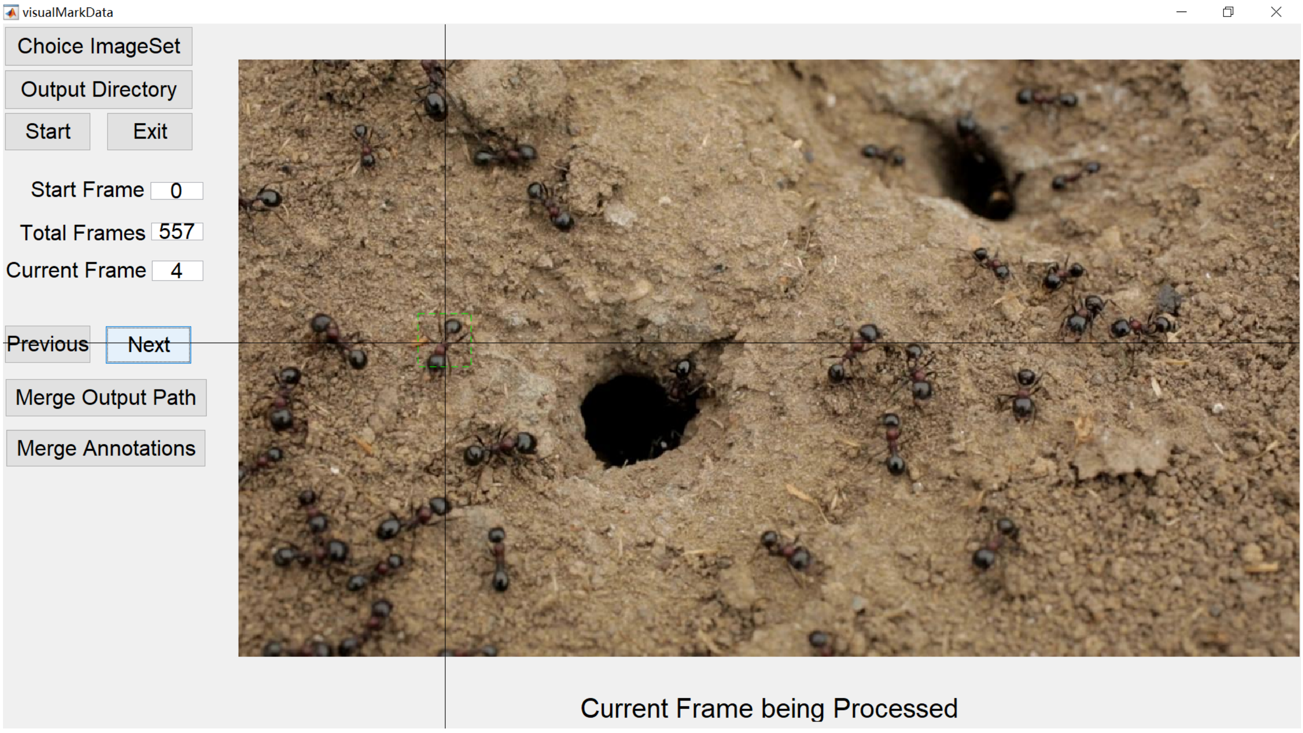

Figure 6. The interactive interface of our image sequence marking software is named VisualMarkData. After selecting an image sequence, the user can acquire the annotation by clicking on the center location of the ant's body.

$$
\begin{aligned}
BD &= AD - AB \\
&= OA \times \tan \angle AOD - OA \times \tan \angle AOB \\
&= OA \times (\tan \angle AOD - \tan \angle AOB) \\
&= OA \times (\tan(\angle AOC + \angle COD) - \tan(\angle AOC - \angle BOC)) \\
&= Height \times (\tan(\angle\alpha + \angle\beta) - \tan(\angle\alpha - \angle\beta))
\end{aligned} \quad (4)
$$

Description of the marking software VisualMarkData

We ~~develop~~ developed an image sequence marking software called VisualMarkData to provide the locations and identification numbers of objects in the sequence for motion analysis. The overall annotation pipeline for the dataset using this software is shown in Figure 1. The operation procedure of VisualMarkData is as follows, and its interface is shown in Figure 6.

- **Choose Image Set.** Before marking, the user should click "Choose ImageSet" to select an image set. The filename of the image set is defined in the format of "SeqXObjectYImageZ", where X is the name of the sequence, Y is the number of objects in the first frame and Z is the size of the bounding box which represents the object. For example, the image set, named "Seq0001Object10Image94", indicates that the sequence "0001" contains 10 objects in the first frame, and each object will be marked with a bounding box with the size of 94x94.
- **Create Output Directory.** The user needs to click "Output Directory" to select the storage path of annotations. Since VisualMarkData only focuses on one object per marking round (each round goes through the whole image sequence), the output folder is suggested to be named with the identification number of the object, e.g. "0001". As the identity number of the object is user-defined, the user can use any number for the object and folder as long as it is unique.
- **Select Start Frame.** In the last step before starting marking, you need to enter the start frame, the default value is 0. This means that you are allowed to exit the software halfway and continue the progress of the current marking task the next time. Then, you can click the "Start" button.
- **Marking.** The user clicks on the center of the object in the current frame, and the software will automatically save the digital location of the center, as well as a bounding box centered on the object. It should be emphasized that the user only marks the same object until finishes the entire image sequence, and then the user can focus on another object by repeating the same operation for the previous one.
- **Next Frame.** The user clicks the "Next" button to show the next frame on the window of the software. The marked location on the previous frame will be displayed with a green-dotted, which can help the user quickly locate the target object.
- **Previous Frame.** If the marked location of the previous frame is incorrect, the user can click the "Previous" button to roll back one frame.
- **Check and Modify.** After the user finishes marking the entire image set, checking is needed to guarantee the quality. In this case, the user can enter the specific frame to modify the annotations by carrying out **Select Start Frame** step.
- **Merge Annotations.** After all objects in a sequence have been marked and reviewed, the user needs to click the "Merge" button, thereby all annotations for each object will be sorted by frames and then the ID of the object both in ascending order.

Availability of source code and requirements

Lists the following:

- Project name: ANTS_marking_and_analysis_tools
- Project home page: e.g., https://github.com/holmescao/ANTS_marking_and_analysis_tools
- Operating system(s): Platform independent
- Programming language: Python, MATLAB, Shell
- Other requirements: MATLAB R2021b (with Image Processing

Toolbox)
- License: MIT License
- RRID: SCR_022543
- biotoolsID identifiers: ants_marking_and_analysis_tools

Data Availability~~of supporting data and materials~~

The dataset supporting the results of this article is available in the ANTS–ant detection and tracking repository at http://dx.doi.org/10.17632/9ws98g4npw.4. Note that the files associated with this dataset are licensed under a Public Domain Dedication license.

Declarations

List of abbreviations

CV: computer vision; ID: identity; FPS: frames per second

Consent for publication

Not applicable

Competing Interests

The authors declare that they have no competing interests.

Funding

This work was supported by the Natural Science Foundation of Fujian Province (No. 2019J01002) and National Nature Science Foundation of China (No. ~~2019J01002).~~ 32071057; No. 61673322; No. 31200769), and was partly supported by the Key project of National Key R&D project (No. 2017YFC1703303).

Author's Contributions

M.W. and S.G conceived the experiment(s), X.C. conducted the experiment(s), X.C. analyzed the results. All authors reviewed the manuscript.

~~Acknowledgements~~

Acknowledgements

The authors thank the reviewers, for providing useful suggestions for improvements and valuable feedback on the workflow and the manuscript.

References

1. Vandermeer J, Perfecto I, Philpott SM. Clusters of ant colonies and robust criticality in a tropical agroecosystem. Nature 2008;451(7177):457–459.
2. Balch T, Khan Z, Veloso M. Automatically tracking and analyzing the behavior of live insect colonies. In: Proceedings of the fifth international conference on Autonomous agents; 2001. p. 521–528.
3. Hölldobler B, Wilson EO, et al. The ants. Harvard University Press; 1990.
4. Whitehouse ME, Jaffe K. Ant wars: combat strategies, territory and nest defence in the leaf-cutting antAtta laevigata. Animal Behaviour 1996;51(6):1207–1217.
5. Vaughan RT, Støy K, Sukhatme GS, Matarić MJ. Whistling in the dark: cooperative trail following in uncertain localization space. In: Proceedings of the fourth international conference on Autonomous agents; 2000. p. 187–194.
6. Fewell JH. Social insect networks. Science 2003;301(5641):1867–1870.
7. Motani M, Srinivasan V, Nuggehalli PS. Peoplenet: engineering a wireless virtual social network. In: Proceedings of the 11th annual international conference on Mobile computing and networking; 2005. p. 243–257.
8. Tiacharoen S, Chatchanayuenyong T. Design and development of an intelligent control by using bee colony optimization technique. American Journal of Applied Sciences 2012;9(9):1464.
9. Poff C, Nguyen H, Kang T, Shin MC. Efficient tracking of ants in long video with GPU and interaction. In: 2012 IEEE Workshop on the Applications of Computer Vision (WACV) IEEE; 2012. p. 57–62.
10. Khan Z, Balch T, Dellaert F. MCMC-based particle filtering for tracking a variable number of interacting targets. IEEE transactions on pattern analysis and machine intelligence 2005;27(11):1805–1819.
11. Khan Z, Balch T, Dellaert F. MCMC data association and sparse factorization updating for real time multitarget tracking with merged and multiple measurements. IEEE transactions on pattern analysis and machine intelligence 2006;28(12):1960–1972.
12. Oh SM, Rehg JM, Dellaert F. Parameterized duration mmodeling for switching linear dynamic systems. In: 2006 IEEE Computer Society Conference on Computer Vision and Pattern Recognition (CVPR'06), vol. 2 IEEE; 2006. p. 1694–1700.
13. Veeraraghavan A, Chellappa R, Srinivasan M. Shape-and-behavior encoded tracking of bee dances. IEEE transactions on pattern analysis and machine intelligence 2008;30(3):463–476.
14. Fletcher M, Dornhaus A, Shin MC. Multiple ant tracking with global foreground maximization and variable target proposal distribution. In: 2011 IEEE Workshop on Applications of Computer Vision (WACV) IEEE; 2011. p. 570–576.
15. Li M, Zhang Z, Huang K, Tan T. Estimating the number of people in crowded scenes by mid based foreground segmentation and head-shoulder detection. In: 2008 19th international conference on pattern recognition IEEE; 2008. p. 1–4.
16. Li Y, Huang C, Nevatia R. Learning to associate: Hybridboosted multi-target tracker for crowded scene. In: 2009 IEEE conference on computer vision and pattern recognition IEEE; 2009. p. 2953–2960.
17. Zhao M, Liu H, Wan Y. An improved Canny edge detection algorithm based on DCT. In: 2015 IEEE International Conference on Progress in Informatics and Computing (PIC) IEEE; 2015. p. 234–237.
18. Schmelzer E, Kastberger G. 'Special agents' trigger social waves in giant honeybees (Apis dorsata). Naturwissenschaften 2009;96(12):1431–1441.
19. Kastberger G, Weihmann F, Hoetzl T. Social waves in giant honeybees (Apis dorsata) elicit nest vibrations. Naturwissenschaften 2013;100(7):595–609.
20. Tan K, Dong S, Li X, Liu X, Wang C, Li J, et al. Honey bee inhibitory signaling is tuned to threat severity and can act as a colony alarm signal. PLoS biology 2016;14(3):e1002423.
21. Dong S, Wen P, Zhang Q, Wang Y, Cheng Y, Tan K, et al. Olfactory eavesdropping of predator alarm pheromone by sympatric but not allopatric prey. Animal Behaviour 2018;141:115–125.
22. Schor N, Bechar A, Ignat T, Dombrovsky A, Elad Y, Berman S. Robotic disease detection in greenhouses: Combined detection of powdery mildew and tomato spotted wilt virus. IEEE Robotics and Automation Letters 2016;1(1):354–360.

23. Wang G, Li W, Zuluaga MA, Pratt R, Patel PA, Aertsen M, et al. Interactive medical image segmentation using deep learning with image-specific fine tuning. IEEE transactions on medical imaging 2018;37(7):1562–1573.
24. Wang C. Research and application of traffic sign detection and recognition based on deep learning. In: 2018 International Conference on Robots & Intelligent System (ICRIS) IEEE; 2018. p. 150–152.
25. Imirzian N, Zhang Y, Kurze C, Loreto RG, Chen DZ, Hughes DP. Automated tracking and analysis of ant trajectories shows variation in forager exploration. Scientific reports 2019;9(1):1–10.
26. Cao X, Guo S, Lin J, Zhang W, Liao M. Online tracking of ants based on deep association metrics: method, dataset and evaluation. Pattern Recognition 2020;103:107233.
27. Sabattini J, Reta J, Bugnon L, Cerrudo J, Sabattini R, Peñalva A, et al. AntVideoRecord: Autonomous system to capture the locomotor activity of leafcutter ants. HardwareX 2022;11:e00270.
28. Yuen J, Russell B, Liu C, Torralba A. Labelme video: Building a video database with human annotations. In: 2009 IEEE 12th International Conference on Computer Vision IEEE; 2009. p. 1451–1458.
29. Vondrick C, Patterson D, Ramanan D. Efficiently scaling up crowdsourced video annotation. International journal of computer vision 2013;101(1):184–204.
30. Doermann D, Mihalcik D. Tools and techniques for video performance evaluation. In: Proceedings 15th International Conference on Pattern Recognition. ICPR-2000, vol. 4 IEEE; 2000. p. 167–170.
31. Biresaw TA, Nawaz T, Ferryman J, Dell AI. Vitbat: Video tracking and behavior annotation tool. In: 2016 13th IEEE International Conference on Advanced Video and Signal Based Surveillance (AVSS) IEEE; 2016. p. 295–301.
32. Nakanishi A, Nishino H, Watanabe H, Yokohari F, Nishikawa M. Sex-specific antennal sensory system in the ant Camponotus japonicus: structure and distribution of sensilla on the flagellum. Cell and tissue research 2009;338(1):79–97.
33. He H, Chen Y, Zhang Y, Wei C. Bacteria associated with gut lumen of Camponotus japonicus Mayr. Environmental Entomology 2011;40(6):1405–1409.
34. Nishikawa M, Watanabe H, Yokohari F. Higher brain centers for social tasks in worker ants, Camponotus japonicus. Journal of Comparative Neurology 2012;520(7):1584–1598.
35. Terayama M, Ogata K. Two new species of the ant genus Probolomyrmex (Hymenoptera, Formicidae) from Japan. Kontyu 1988;56(3):590–594.
36. Wang L, Chen J. Fatty amines from little black ants, Monomorium minimum, and their biological activities against red imported fire ants, Solenopsis invicta. Journal of chemical ecology 2015;41(8):708–715.
37. Thompson C. Ants that have pest status in the United States. In: Applied Myrmecology CRC press; 2019.p. 51–67.
38. Sanders C. The Biology of Carpenter Ants in New Brunswick1. The Canadian Entomologist 1964;96(6):894–909.
39. Carney WP. Behavioral and morphological changes in carpenter ants harboring dicrocoeliid metacercariae. The American Midland Naturalist 1969;82(2):605–611.
40. Carlin NF, Hölldobler B. The kin recognition system of carpenter ants (Camponotus spp.). Behavioral Ecology and Sociobiology 1986;19(2):123–134.
41. Ayieko MA, Kinyuru J, Ndong'a M, Kenji G. Nutritional value and consumption of black ants (Carebara vidua Smith) from the Lake Victoria region in Kenya. Advance Journal of Food Science and Technology 2012;.
42. Leal-Taixé L, Milan A, Reid I, Roth S, Schindler K. Motchallenge 2015: Towards a benchmark for multi-target tracking. arXiv preprint arXiv:150401942 2015;.
43. Fabbri R, Costa LDF, Torelli JC, Bruno OM. 2D Euclidean distance transform algorithms: A comparative survey. ACM Computing Surveys (CSUR) 2008;40(1):1–44.
44. Wang Q, Song W, Zhang J, Lo S. Bi-directional movement characteristics of Camponotus japonicus ants during nest relocation. Journal of Experimental Biology 2018;221(18):jeb181669.
45. Bond AB. Optimal foraging in a uniform habitat: the search mechanism of the green lacewing. Animal Behaviour 1980;28(1):10–19.
46. Deffernez L, Champagne P, Verhaeghe JC, Josens G, Loreau M. Analysis of the spatio-temporal niche of foraging grassland ants in the field. Insectes sociaux 1990;37(1):1–13.
47. Feener DH, Moss KA. Defense against parasites by hitchhikers in leaf-cutting ants: a quantitative assessment. Behavioral ecology and sociobiology 1990;26(1):17–29.
48. Loreto RG, Hart AG, Pereira TM, Freitas ML, Hughes DP, Elliot SL. Foraging ants trade off further for faster: use of natural bridges and trunk trail permanency in carpenter ants. Naturwissenschaften 2013;100(10):957–963.
49. Wu M, Cao X, Guo S. Swarm behavior tracking based on a deep vision algorithm. arXiv preprint arXiv:220403319 2022;.

response letter Click here to access/download;Manuscript;response_letter.pdf

Response Letter to GigaScience Submission

Paper ID: GIGA-D-22-00055R1

Paper Title: A dataset of ant colonies motion trajectories in indoor and outdoor scenes for social cluster behavior study

We want to thank you for your valuable comments.

We submitted 3 files, including a revised manuscript, a track changes file (to highlight differences between the revised and the original manuscript), and this file, i.e., the response letter (a complete response to the editor and all reviewers). In the following, we respond to each of your concerns and recommendations.

(EC: Editor's Comment, RC: Reviewer's Comment, AR: Authors' Response)

Editor

EC0: In particular, the reviewers mention the need to add more specific information and metadata to make the data set useful for others (including, for example, the scientific name of the ant species used and more exact details on data acquisition - see the reports below). In addition to the reviewers' reports, some minor editorial points from my side:

AR0: We thank the editor and reviewers for their kind comments and responded in a point-by-point manner.

EC1: We will consider the submission for publication as a "Data Note" - please check our instructions or authors for formatting guidelines for this article type (you do not need to change the text in a major way, but please try to use the section headings as indicated for the "Data Note" article type, if applicable). (Please also note that the Data Note article type has a lower Article Processing Charge than "Research".)

AR1: We modified the article to "Data Note" type by referring to the formatting guidelines on the journal website.

EC2: On page 2, you mention that data is available "subject to a royalty-free license", but at the end you provide another source which is public domain /cc0 (which is great). If the data is available under public domain, I recommend you remove the alternative source, or clarify if the data fulfills public domain criteria - (or are these different data sets? If you need help to make data available, we can also store it under a cc0 licence on our server GigaDB.)

AR2: We removed the alternative sources mentioned in the second page in the revised manuscript.

EC3: In the methods section, I recommend the use of past tense for the procedures that you carried out. ("We collected ants" instead of "we collect ants", etc..). Please also include the helpful wording suggestions from the reviewers' reports.

AR3: In the Data Description section and the Methods section of the revised manuscript, we have changed the procedures that are carried out to the past tense (see details in lines 96-124 and 274-303 of the revised manuscript).

EC4: In addition, please register the new software application in the bio.tools and SciCrunch.org databases to receive RRID (Research Resource Identification Initiative ID) and biotoolsID identifiers, and include these in your manuscript (in the code availability section). This will facilitate tracking, reproducibility and re-use of your tool.

AR4: We registered in bio.tools and SciCrunch.org respectively. The biotoolsID identifiers we obtained are ants_marking_and_analysis_tools and the RRID is SCR_022543. These information can be found in the Availability of source code and requirements section in lines 365-366 of the revised manuscript.

Reviewer reports:

Reviewer #1

RC1.0: Authors proposed a new software to evaluate ant movement, very useful to understand ant behavior and also to use their patterns to apply to other areas of science. I found a very interesting manuscript, well written and well addresses as well. This is a great advance in the methods to evaluate ant foraging, which I feel is useful for scientific researchers from many areas. I have some suggestions that I hope can help the authors strengthen the manuscript, making it clear to readers.

AR1.0: We thank the reviewer for the kind comments.

RC1.1: [Abstract] Lines 1,2: The clustering mechanism is most related to inside the colony than outside, in terms of the behavior of ant colonies that cluster their corpses and sort their larvae inside the colony. Or it could be valid for outside the colony like the authors made, at the entrance of the nest?

AR1.1: As you pointed out, clustering mechanism is most related to inside the colony, hence what we want to explore is the mechanism inside the colony. However, it seems that our statement is not clear enough. The so-called "indoor" and "outdoor" refer to the laboratory environment with a clean background (as shown in Figure 2a) and the natural environment (as shown in Figure 3a), respectively. We modified the abstract to eliminate ambiguity, as detailed in lines 2-3 of the revised manuscript, and shown below.

Origin: "Most studies in the field of ant behavior have only focused on indoor environments, while outdoor environments are still underexplored."

Revision: "Most studies in the field of ant behavior have only focused on indoor environments (laboratory setup), while outdoor environments (natural environments) are still underexplored."

RC1.2: [Abstract] Line 3: Include how many videos were taken indoors and outdoor.

AR1.2: We have 10 videos in total, 5 of which are indoor (laboratory setting) scene and 5 of which are outdoor (natural environment) scene. The comparison before and after modification is as follows (see details in lines 3-4 of the revised manuscript):

Origin: "In this paper, we collect 10 videos of ant colonies from different indoor and outdoor scenes."

Revision: "In this paper, for the first time, we collect 10 videos and 3 species of ant colonies from different scenes, including 5 indoor and 5 outdoor scenes."

RC1.3: [Abstract] Line 3: Maybe "And" instead of "In addition".

AR1.3: "In addition" has been changed to "And". The comparison before and after modification is as follows (see details in line 4 of the revised manuscript):

Origin: "In addition, we develop an image sequence marking software named VisualMarkData …"

Revision: "And we develop an image sequence marking software named VisualMarkData …"

RC1.4: [Abstract] Line 5: Maybe using the notation "114,112" instead of "114112".

AR1.4: "114112" has been changed to "114,112". The comparison before and after modification is as follows (see details in lines 10-11 of the revised manuscript):

Origin: "In all 5354 frames, the location information and the identification number of each ant are recorded for a total of 712 ants and 114112 annotations.”

Revision: "In all 5,354 frames, the location information and the identification number of each ant are recorded for a total of 712 ants and 114,112 annotations.”

RC1.5: [Introduction] Line 2 §2: The quality of what? From the information acquired?

AR1.5: To be more specific, we need to ensure the quality of the annotations, i.e., the accuracy. The comparison before and after modification is as follows (see details in lines 12-14 of the revised manuscript):

Origin: "Until the late 20th century, biologists still manually marked the motion trajectories on the video to guarantee the quality."

Revision: "Until the late 20th century, biologists still manually tracked the motion trajectories on the video to guarantee the accuracy of the marking."

RC1.6: [Introduction] Lines 5 §2: The final dot is after the reference: "[9].

AR1.6: The final dot has been placed after the reference. The comparison before and after modification is as follows (see details in line 16 of the revised manuscript):

Origin: ". [9]"

Revision: "[9]."

RC1.7: [Introduction] Lines 5,6 §2: I suggest removing the term "Obviously". Besides, the development of an automated tracking process is also time-consuming in relation to setting and calibrating the system (visual confirmation, etc.), right? And also, the automated tracking process can be more expensive than doing manually, right? If there is some previous work comparing the trade-off cost/benefit it could be included in the citation in order to better base the author's arguments.

AR1.7: "Obviously" has been removed. The comparison before and after modification is as follows (see details in line 16 of the revised manuscript):

Origin: "Obviously, manually tracking is time-consuming and prone to human error."

Revision: "Manually tracking is time-consuming and prone to human error."

Besides, the development of an automatic tracking process is indeed time-consuming. However, we only need to develop it once. It is reusable for same tasks in the future. Once development finished, additional manually making time for every video will no longer be needed.

RC1.8: [Introduction] Line 1 §3: "release" instead of "free"?

AR1.8: "Free" has been modified to "release". The comparison before and after modification is as follows (see details in line 22 of the revised manuscript):

Origin: "Traditional CV techniques free researchers …"

Revision: "Traditional CV techniques release researchers …"

RC1.9: [Introduction] Lines 12, 13 §4: Regarding "only one outdoor scene dataset": Which one?

AR1.9: We want to express that only a single outdoor scene sequence is used in all the current studies. References to these studies have been added now. The comparison before and after modification is as follows (see details in lines 42-44 of the revised manuscript):

Origin: "To the best of our knowledge, however, the current studies are all using only one outdoor-scene dataset, which is lack of data diversity."

Revision: "To the best of our knowledge, however, only a few works~\cite{imirzian2019automated, cao2020online} annotate motion trajectories in videos, and both use only a single outdoor scene sequence, which lacks data diversity."

RC1.10: [Introduction] §4: I would suggest using "5,354" and "114,112" instead of "5354" and "114112".

AR1.10: We totally agree with your suggestion. "5354" and "114112" have been modified to "5,354" and "114,112". The comparison before and after modification is as follows (see details in lines 82-83 of the revised manuscript):

Origin: "The total size of our dataset is 5354 frames, 712 ants, and 114112 labels."

Revision: "The total size of the dataset is 5,354 frames, 712 ants, and 114,112 labels."

RC1.11: [Data Description] Line 2 §2: What is the time interval of each frame taken from the video?

AR1.11: In Table 1 of the revised manuscript, column 3 indicates the FPS (frames per second) of the video. Specifically, Seq0001~Seq0005 is 25 frames per second, the interval between two frames is 40 ms. Seq0006~Seq0010 is 30 frames per second, that is, the interval between two frames is 33 ms.

RC1.12: [Data acquisition] Line 1 §1: "ant" instead of "ants".

AR1.12: "ants" has been modified to "ant". The comparison before and after modification is as follows (see details in lines 99-100 of the revised manuscript):

Origin: "We collect 50 workers of Japanese arched ants species …".

Revision: "We collect 50 workers of Japanese arched ant species …".

RC1.13: [Data acquisition] Regarding the links, https://cn.depositphotos.com/home.html and https://data.mendeley.com/datasets/9ws98g4npw/3: How stable are those links? Does the journal require Dryad/Github or another repository?

AR1.13: We removed the first link because its information was already included in our dataset. For the second link, the Mendeley Data continues to provide open-source data repositories to researchers. They claim on their official website that the DOI of this dataset is available always: "To ensure the highest level of integrity and security possible , data is stored on Amazon's S3 servers in Ireland. Our service was extensively penetration tested and received certification. Additionally, your published datasets are archived with Data Archiving and Network Services (DANS) to preserve your data over the long term. DANS is a long-term archiving provider, which is an institute of the Dutch Academy KNAW, and of the Netherlands' national research council, NWO. We contract with DANS to archive all valid published datasets in perpetuity. The agreement ensures that the DOIs we provide for datasets will always resolve to a web page, where the dataset metadata and files will be available. Data archived at DANS is backed up and stored in three locations for redundancy."

Besides, the journal allows us to choose other repositories.

RC1.14: [Det folder] Provide the information you want to show and include the source between parenthesis. Do not start with the "The 'det' folder…"

AR1.14: Thank you for this suggestion, according to your suggestion, we modified the expression and the comparison before and after modification is as follows (see details in lines 134-139 of the revised manuscript):

Origin: "The 'det' folder contains a 'det.txt' file which is the ground truth for detection, recording the location parameters of the ants in all frames, which is similar to multi-object tracking challenge~\cite{leal2015motchallenge}. Each line represents one ant instance, and it contains 7 values as shown in Table~\ref{tab:data_format}."

Revision: "In the same format as the dataset of multi-object tracking challenge~\cite{leal2015motchallenge}, we record information such as the identity and location parameters of all ants in each frame for detection. Such information is stored in a 'det.txt' file in a folder named 'det' in our dataset. Concretely, each line represents one ant instance, and it contains 7 values (also called attributes) as shown in Table~\ref{tab:data_format}."

RC1.15: [Det folder] Line 3: "which is similar to multi-object tracking challenge": And how is it? Similar how?

AR1.15: The multi-object tracking challenge~\cite{leal2015motchallenge} we describe here is a dataset, and the format of our dataset is the same as that. We modified the expression, and the comparison before and after modification is as follows (see details in lines 134-137 of the revised manuscript):

Origin: "The 'det' folder contains a 'det.txt' file which is the ground truth for detection, recording the location parameters of the ants in all frames, which is similar to multi-object tracking challenge~\cite{leal2015motchallenge}."

Revision: "In the same format as the dataset of multi-object tracking challenge~\cite{leal2015motchallenge}, we record information such as the identity and location parameters of all ants in each frame for detection. Such information is stored in a 'det.txt' file in a folder named 'det' in our dataset."

Besides, the specific format can be seen in lines 139-147 or Table 2 of the revised manuscript.

RC1.16: [Det folder] Line 4: "the position of one ant at one instant classified into seven categories (values 1-7)"? instead of "one ant instance and it contains 7 values"

AR1.16: We use 7 attributes to represent each ant instance, including frame number, identity number, bounding box left, bounding box top, bounding box width, bounding box height, and confidence score (see Table 2 for details). However, it seems that there was an ambiguity in our original statement, so we revised it. The comparison before and after modification is as follows (see details in lines 138-139 of the revised manuscript):

Origin: "Each line represents one ant instance, and it contains 7 values as shown in Table~\ref{tab:data_format}."

Revision: "Concretely, each line represents one ant instance, and it contains 7 values (also called attributes) as shown in Table~\ref{tab:data_format}."

RC1.17: [Gt folder] The same for Det folder. Describe the results, and the information, and then reference the source.

AR1.17: Thank you for this suggestion, according to your suggestion, we modified the expression, and the comparison before and after modification is as follows (see details in lines 149-153 of the revised manuscript):

Origin: "The 'gt' folder contains a 'gt.txt' file, which is the ground truth for muti-object tracking. Similar to the 'det.txt' file, it also contains 7 values (see details in Table~\ref{tab:data_format})."

Revision: "In our dataset, we provide groundtruth records for multi-object tracking. These information are stored in a 'gt.txt' file in a folder named 'gt'. Similar to the previous description of the 'det.txt' file, the records of each instance in the 'gt.txt' file also contains 7 values (also called attributes), see Table~\ref{tab:data_format} for details."

RC1.18: [Img folder] The same as the previous comment. Also, after "e.g." there is a comma: "e.g.,"

AR1.18: Thank you for this suggestion, according to your suggestion, we modified the expression and the comparison before and after modification is as follows (see details in lines 159-160 of the revised manuscript):

Origin: "The 'img' folder stores the original image sequence converted from the video."

Revision: "In our dataset, we provide the original image sequence converted from the video, which are stored in the 'img' folder."

Besides, "e.g.”has been modified to "e.g.,", The comparison before and after modification is as follows (see details in lines 161-162 of the revised manuscript):

Origin: "All images are converted to JPEG and named sequentially to a 6-digit file name (e.g. 000001.jpg)"

Revision: "All images are converted to JPEG and named sequentially to a 6-digit file name (e.g., 000001.jpg)"

RC1.19: [Analyses] Visually confirmation instead of "Visually confirm"

AR1.19: "Visually confirm" has been modified to "Visually confirmation". The comparison before and after modification is as follows (see details in line 164 of the revised manuscript):

Origin: "Visually confirm"

Revision: "Visually confirmation"

RC1.20: [Analyses] Please describe how many people did it, if it expected some bias depending on the person that check the images? Also: Is it necessary to do with all images? How much time does it take? Can you all estimate the labor time, is it?

AR1.20:

Q1: Please describe how many people did it, if it expected some bias depending on the person that check the images?

A1: We have 2 staff marking indoor videos and 3 staff marking outdoor videos. And there is only one inspector, so the bias is avoided. (See details in lines 165-168 of the revised manuscript)

Q2: Is it necessary to check all images?

A2: In the sequence-level checking, all sequences need to be checked. In the image-level checking, Only the sequence that is considered to be of low quality needs to have each frame checked. (See details in Figure 4 and lines 170-181 of the revised manuscript)

Q3: How much time does it take? Can you all estimate the labor time, is it?

A3: In the sequence-level checking, depending on how many ants are in each video, the time cost ranges from 8 to 10 times the length of the original video sequence. In the image-level checking, the check speed is about 0.5 seconds/ant, and the repair time is about 2 seconds/ant. (See details in lines 177-179 and 184-185 of the revised manuscript)

A comparison of the above three questions involving the content before and after the revision is as follows (see details in lines 165-185 of the revised manuscript):

Origin: "The ground truth annotations for all image sequences in the dataset were visually confirmed by the data annotation staff. The visual reviewing consists of two aspects, sequence-level (coarse-grained) and image-level (fine-grained). Firstly, staffs performed a coarse-grained review of a single sequence. Specifically, we drew the annotations on the corresponding images, and then converted the image sequence to video. By replaying the video, staff can quickly confirm which segments of the video are poor quality and needed to be re-marked. For each scene, an example image frame is shown in Figure~\ref{fig:tech_val_in} (a) and Figure~\ref{fig:tech_val_out} (a). After that, staff reviewed the quality of annotations frame-by-frame via VisualMarkData. For inaccurate annotations, staff modified manually by using the "Check and modify" function of VisualMarkData (see details in Methods)."

Revision: "In all 10 videos, we have 2 staff to mark indoor videos and 3 staff to mark outdoor videos. Furthermore, the ground-truth annotations for all image sequences in the dataset were visually confirmed by one staff. The visual reviewing consists of two aspects, sequence-level (coarse-grained) and image-level (fine-grained). Firstly, the staff performed a coarse-grained review of a single sequence. Specifically, we drew the annotations on the corresponding images, and then converted the image sequence to video. For each scene, an example image frame is shown in Figure~\ref{fig:tech_val_in} (a) and Figure~\ref{fig:tech_val_out} (a). By replaying the video, staff can quickly confirm which segments of the video are of poor quality and needed to be re-marked. Figure~\ref{fig:low_quality} (a) shows an example of a segment distinguished as low-quality annotations. The sequence-level checking time consumption per video is 8 to 10 times the original video sequence duration, which depends on the number of ants in the video. After that, staff reviewed the quality of annotations frame-by-frame via VisualMarkData. For inaccurate annotations, staff modified manually by using the "Check and modify" function of VisualMarkData (see details in Methods). Figure~\ref{fig:low_quality} (b) shows the modified annotations. The image-level checking speed is about 0.5 sec per ant instance while correction takes about 2 sec per ant instance."

RC1.21: [Analyses] After the equation 3: "is set to o": I guess this is an "o" and not a "0" (zero).

AR1.21: Here v0 is set to zero, because we assume that the ants are stationary at the initial moment. We added this assumption after the expression. The comparison before and after modification is as follows (see details in lines 187-188 of the revised manuscript):

Origin: "Where, the v_{0} is set to 0."

Revision: "Where, the v_{0} is set to 0, i.e. we assumed that the ants were stationary at the initial moment."

RC1.22: [Discussion] Lines 9-11 §1: Where are the Python Scripts available?

AR1.22: Links to the Python scripts have been added now. The comparison before and after modification is as follows (see details in lines 207-210 of the revised manuscript):

Origin: "Alongside, we have provided publicly available Python Scripts to illustrate the analysis of data as well as usage of the data."

Revision: "Alongside, we have provided publicly available Python Scripts at ~\url{https://github.com/holmescao/ANTS_marking_and_analysis_tools}, to illustrate the analysis of data as well as usage of the data."

RC1.23: [Discussion] Line 15: "the" instead of "our".

AR1.23: “our” has been modified to “the”. The comparison before and after modification is as follows (see details in line 214 of the revised manuscript):

Origin: "… evaluate the tracking accuracy on our dataset."

Revision: "… evaluate the tracking accuracy on the dataset."

RC1.24: [Discussion] Lines 1-3 §2: How it would be made? When it would be available? I think that promising something in a paper is not appropriate. Maybe the authors can only tell something parsimonious like: "it is possible that some updates will be done in the future".

AR1.24: We highly agree with your suggestion, and we modified the sentence expression and the comparison before and after modification is as follows (see details in lines 218-220 of the revised manuscript):

Origin: "In the future, we will enrich the VisualMarkData with more features to reduce the difficulty of marking and improve the efficiency of marking."

Revision: "In the future, it is possible that VisualMarkData software will be made some updates to reduce the difficulty of marking and improve the efficiency of marking."

RC1.25: [Potential implications] It is a bit vague about the implications (and also applications). Please provide citations of previous articles that used the manual tracking and conclusions they made and mention how this technique can improve the reach of these studies in terms of investigating outdoor nests and also more replicates.

AR1.25: According to your suggestion, we modified the potential implications section (The current version is called Potential usage of dataset). The comparison before and after modification is as follows (see details in lines 237-270 of the revised manuscript):

Origin: "Swarm behavior is one of the most important features of social insects, which has important significance for the study of embodied intelligence~\cite{tiacharoen2012design}. Specifically, social insects often tend to cluster into a colony~\cite{vandermeer2008clusters}, which forms a complex dynamical system together with the surrounding environment~\cite{balch2001automatically}. So far, researchers do not know enough about the mechanisms behind swarm behaviors of social insects. We believe our image sequence marking software and dataset could facilitate the analysis of ant colony behavior leading to the development of embodied intelligence."

Revision: " Swarming behavior is one of the most important features of social insects~\cite{vandermeer2008clusters}, often involving division of labor~\cite{holldobler1990ants}, task specialization~\cite{whitehouse1996ant}, distributed problem solving~\cite{vaughan2000whistling}, etc. To reveal the mechanisms behind swarming behavior requires observing insect colonies over long periods of time as well as recording the motion trajectory of each individual~\cite{poff2012efficient}. Before the advance of computer vision technology, biologists utilize manual tracking to study insect behaviors~\cite{bond1980optimal, deffernez1990analysis}. Since manual recording is time-consuming and laborious, biologists focus only on individual behavioral studies, including foraging activity~\cite{deffernez1990analysis} and prey avoidance~\cite{bond1980optimal}, etc. In recent years, in order to enable rapid tracking of the activities of multiple insects simultaneously, automated image-based tracking techniques are employed, and a lot of attempts are made to improve the accuracy of tracking~\cite{khan2005mcmc, khan2006mcmc, oh2006parameterized, veeraraghavan2008shape, fletcher2011multiple}. These techniques have assisted biologists to discover some colony mechanisms. For example, Balch T et al~\cite{balch2001automatically} found that a number of ants would interact at the entrance of the nest when some find food nearby. However, current studies are almost limited to laboratory settings with clean backgrounds. Such approaches disregard the influence of the environment surrounding the insect colony, including potential predators~\cite{feener1990defense}, obstacles on the road~\cite{loreto2013foraging}, etc. In contrast, we provide labeled motion trajectories of ant colonies active outdoors, containing a variety of scenes. These data can be used to train deep learning models for automated tracking ants in natural environments. Moreover, we already used indoor/Japanese arched ants' images as the training set in our previous work~\cite{cao2020online} and tested our model on outdoor/black ant images (Seq0010), achieving a tracking accuracy up to 92\%. Vice versa, we also conducted experiments using outdoor images as the training set and indoor images as the test set, which are presented in a method manuscript that we are preparing~\cite{wu2022swarm}, which can be found at arXiv. Hence, it will help biologists to quantify and analyze the foraging patterns of ant colonies in natural environments, such as foraging strategies, partner gathering, and collaborative transportation."

RC1.26: [Methods] More information is required here (or can be placed in the Data Description section). Which ant species do the ant colonies belong to? Which size (more or less) do the ant workers have? Outside or inside the nest? Close to the entrance? What angle of view was used, i.e., distance from ground? The height where the camera was placed? Which camera was used? Area of the frames? Description of the background? The background was cleaned prior to the recording? Etc.

AR1.26: We added the information you suggested and below are the answers to your questions.

Q1: Which ant species do the ant colonies belong to?

R1: Column 7 in the bottom part of Table 1, and lines 96 and 111 of the revised manuscript describe the species of ants of each video.

Q2: Which size (more or less) do the ant workers have?

R2: The ants in each scene are all worker ants, see lines 99-100 and 112-114 in the revised manuscript for details.

Q3: Outside or inside the nest?

R3: No nest in the indoor scenes. Outdoor scenes are filmed outside the nest except for Seq0010. See lines 103-104 and 121-122 in the revised manuscript for details.

Q4: Close to the entrance?

R4: Only the four video scenes of outdoor scenes are close to the entrance, including Seq0006 to Seq0009. See Table 1 and lines 121-122 of the revised manuscript for details.

Q5&Q6: What angle of view was used, i.e., distance from ground? The height where the camera was placed?

R5&A6: Table 1, Figure 5, and lines 281-285 and 297-301 of the revised manuscript describe the camera angle, the field of view of the camera, and the height of the camera from the ground for each video sequence.

Q7: Which camera was used?

A7: Table 1, lines 278-280 and 295-296 of the revised manuscript describe the camera types.

Q8：Area of the frames?

A8: Table 1 gives the area of each scene (length times width). Figure 5, Equation 4, and lines 285-290, 301-303 of the revised manuscript describe how the area is calculated.

Q9&Q10：Description of the background? The background was cleaned prior to the recording?

A9&A10: Lines 103-104 and 116-122 of the revised manuscript describe the backgrounds of scenes.

Besides, we added information about the date, location, and temperature during the filming, as detailed in Table 1.

RC1.27: [Methods] Figure 1: Figure 1 was never referenced in the text. Line 4, Caption of Figure 1: Describe each of the three visualization tools to verify the data quality.

AR1.27: We have added the mention of Figure 1 in 2 places of the context. The added information is as follow (see details in lines 80-81 and 307-308 of the revised manuscript):

Add: "Then utilizing VisualMarkData and following the process shown in Figure~\ref{fig:pipeline}, …"

Add: "The overall annotation pipeline for the dataset using this software is shown in Figure~\ref{fig:pipeline}."

About caption of Figure 1, we added detail descriptions of three Python scripts. The comparison before and after modification is as follows (please see the description of Figure 1 lines 4-5):

Origin: "Additionally, we provide three visualization tools to verify the data quality."

Revision: "Additionally, three Python scripts are provided to generate three visualization results to verify the quality of the data, including the trajectories drawn on the original graph, the heat map of motion velocity, and the histogram of the frequency distribution of motion velocity."

RC1.28: [Methods] Figure 2: I suggest changing Velocity to Speed, prefer the scalar rather than the vector.

AR1.28: “Velocity” in Figure 2 has been modified to “Speed”, as detailed in Figure 2.

Besides, all the "velocity" in the manuscript have been replaced by "speed”.

RC1.29: [Methods] Line 2, Caption of Figure 2: "five consecutive sequences" instead of "5 sequences" and then remove ", respectively". "Histogram of the frequency of ant speeds in cm/s for indoor sequences" instead of "Ant speed histograms per indoor sequence".

AR1.29: We fully adopt your suggestions. The comparison before and after modification is as follows (see details in Figure 2 lines 1-2):

Origin: "(b) Speed distributions in image space for 5 sequences of indoor scenes, respectively. (c) Ant speed histograms per indoor sequence."

Revision: "(b) Speed distributions in image space for five consecutive sequences of indoor scenes. (c) Histogram of the frequency of ant speeds in cm/s for indoor sequences."

RC1.30: [Methods] Table 1: Describe the meaning of each column, Sequence is ...XXX, FPS is ...XXX, etc. Maybe "statistics" is not the appropriate term; prefer the use of "description" or something like that.

AR1.30: We described the meaning of each column and changed "Statistics" to "Description". It should be noted that we expanded the column of Table 1 based on other comments. The information before and after the modification of caption is as follows (see details in Table 1):

Origin: "Statistics of ant videos with annotations in indoor and outdoor scenes."

Revision: "Description of ant videos with annotations in indoor and outdoor scenes. Top part provides filming details. Sequence = Name of video for each colony. Angle = Angle of the camera during filming. Height = Height of camera from the ground. Temp = Local temperature during filming. Datetime = Date and time of the filming. Location = Location of the filming. Camera = Camera type. Bottom part provides a description of ant videos with annotations. FPS = Frame rate of the video. Resolution = The resolution of the video. Length = The number of frames of the video, with the duration in parentheses. Ants = The number of ants with different IDs that appear in the video. Annotations = The number of ants instances labeled in the video. Species = The ant species. Entrance = Whether the colony is active at the nest entrance. Area = The area of the filmed scene. Note that the camera's angle of view is 16° and 7.5° in the horizontal and vertical directions, respectively, which are not represented in the table."

RC1.31: [Methods] Table 2: Maybe the files could be provided as an appendix for the paper.

AR1.31: Thank you for your suggestion. However, due to each file contains thousands of lines, it would be inconvenient to add the files directly to the manuscript's appendix. Thus, we published the files in https://data.mendeley.com/datasets/9ws98g4npw/3, which is convenient for users to view and download.

Reviewer #2

RC2.0: This paper developed the image sequence marking software VisualMarkData and tracked ants' locomotion in indoor and outdoor environments. All data of these images are available. Developing new tracking software is important for animal behaviour and computational biology. However, it wasn't easy to follow the novelty of this study and its importance as biological research.

AR2.0: We thank the reviewer for the kind comments.

RC2.1: [Major comments] What is the novel point of the image sequence marking software VisualMarkData? The authors have mentioned outdoor and indoor, but how did you overcome this difficulty?

AR2.1: For the first question, the main contribution of VisualMarkData is that it offers: (1) a comprehensive annotation of states at individual-target as well as group-target level; (2) representation of annotations (together with their IDs and behavior labels) of multiple targets and multiple groups in a simple-to-access matrix format; (3) a simple and efficient visualization during annotation, which presents the annotation information of the previous frame as a reference and then only requires clicking on the center point of each target to complete the annotation; and (4) a windows-based friendly graphical user interface that minimizes labor and maximizes annotation quality. We revised the abstract and the introduction (current version is context) about the VisualMarkData to highlight these contributions, as described in lines 4-10 and 62-74, respectively, of the revised manuscript.

In addition, since the type of our manuscript is "Data Note", we focus on providing a detailed approach to data production, validation, and potential reuse, which is in line with the instructions on the journal's website, see https://academic.oup.com/gigascience/ pages/data_note#Criteria. We are the first to construct the ant colony activity dataset with annotations with multiple species and colonies in both indoor and outdoor environments. Specifically, the dataset contains 3 species and 10 colonies with a total of 5,354 frames, 712 ants, and 114,112 labels.

Where the revision of the descriptions of the abstract is shown below (It can also be found in lines 4-10 of the revised manuscript abstract).

Origin: "And we develop an image sequence marking software named VisualMarkData, which enables us to provide annotations of ants in the video."

Revision: "And we develop an image sequence marking software named VisualMarkData, which enables us to provide annotations of ants in the video: (1) offers a comprehensive annotation of states at individual-target as well as colony-target level; (2) provides a simple matrix format to represent multiple targets and multiple groups of annotations (along with their IDs and behavior labels); (3) during the annotation process, we propose a simple and effective visualization that takes the annotation information of the previous frame as a reference and then simply clicks on the

center point of each target to complete the annotation; and (4) we develop a user-friendly windows-based GUI to minimize labor and maximize annotation quality."

Where the revision of the descriptions of the introduction (called context in the current version) is shown below (it can also be found in lines 62-74 of the revised manuscript).

Origin: "Besides, we develop an image sequence mark software named VisualMarkData, which is used to mark the pixel patches covered by ants in each frame of the video."

Revision: "With respect to the tool, we propose the VisualMarkData, which allows users to generate ground-truth information of multi-target motion trajectories in video sequences. Specifically, VisualMarkData offers: (1) a comprehensive annotation of states at individual-target as well as group-target level; (2) representation of annotations (together with their IDs and behavior labels) of multiple targets and multiple groups in a simple-to-access matrix format; (3) a simple and efficient visualization during annotation, which presents the annotation information of the previous frame as a reference and then only requires clicking on the center point of each target to complete the annotation; and (4) a windows-based friendly graphical user interface that minimizes labor and maximizes annotation quality."

For the second question, there is no difference in the way we annotate the indoor and outdoor scene videos, and the detailed annotation method is described in lines 311-354 of the revised manuscript.

As for the method of data acquisition, we provide a detailed description in the Data Description section, including both indoor and outdoor (added in the current version) ways, which are described in lines 95-124 of the revised manuscript.

RC2.2: [Major comments] What is the purpose of providing your dataset for training or future research? If it is for training, the authors will show that training using indoor/Japanese arched ants' images can be useful for tracking outdoor/black ant images or vice versa. If authors want other researchers re-analyze these data for any reason, basic information is lacking. The species name is very important; the authors need to mention it. It is better to provide the temperature, date, time, and location of the colony when they took video in the outdoor environment.

AR2.2: The main purpose of providing this data set is to train automated models for biologists to better understand the clustering behavior of insects, as described in lines 6-9 of the revised version. In the meanwhile, our dataset can also be used for future research.

For training, we already used indoor/Japanese arched ants' images as the training set in our previous work~\cite{cao2020online} and tested our model on outdoor/black ant images, achieving a tracking accuracy up to 92%. Vice versa, we also conducted experiments using outdoor images as the training set and indoor images as the test set, which are presented in a method manuscript

that we are preparing~\cite{wu2022swarm}, which can be found at arXiv. These details are added in lines 260-267 of the Potential implications (named Potential usage of dataset in the current version) section of the revised manuscript.

For future research, we added relevant information so that other researchers re-analyze these data for any reason. Additional information includes:

- Colony size
- Species name
- Nest exist or not in the scene
- Relationship between the scene and the entrance of the nest
- The background of the scene is processed or not
- The background description of the filming scene
- Temperature during the filming
- Filming date and time
- Filming location
- Camera type and parameter
- Camera angle
- Filming height of camera
- The field of view of camera
- The area of the filming scene and its measure method

This information is supplemented in Table 1, Figure 5, Equation 4, lines 95-124, 277-290 and 295-303 of the revised manuscript.

RC2.3: [Major comments] Why did you use two species? Did you collect two species of ants and record their behaviour under both indoor and outdoor conditions? If not, why? Can we use data of different ant species as training data? For example, if you use data of Japanese arched ants as training, can you detect the black ant?

AR2.3: We use three species, including Japanese arched ants (also called Camponotus japonicus ants)~\cite{nakanishi2009sex, he2011bacteria, nishikawa2012higher}, Little black ants~\cite{wang2015fatty, thompson2019ants} and Carpenter ants~\cite{sanders1964biology, carney1969behavioral, carlin1986kin}, which are widely studied by behavioral ecologists and social biologists. Since Japanese arched ants are often domesticated, they are suitable for observation in laboratory environments. As for the other two species of ants, we only record their behavior outdoors and do not catch them in laboratory environments so as not to disturb their normal life. It is in accordance with academic ethics that ant citizens should not be violated casually to prevent ecological damage. In the revised manuscript, the species of ants in each video are shown in Table 1, and the relevant descriptions can be found in lines 96-99 and 111-114.

In addition, we can use data of different ant species as training data. we already used indoor/Japanese arched ants' images as the training set in our previous work~\cite{cao2020online}

and tested our model on outdoor/black ant images, achieving a tracking accuracy up to 92%. Vice versa, we also conducted experiments using outdoor images as the training set and indoor images as the test set, which are presented in a method manuscript that we are preparing~\cite{wu2022swarm}, which can be found at arXiv. These details are added in lines 260-267 of the Potential implications (named Potential usage of dataset in the current version) section of the revised manuscript.

RC2.4: [Minor comments] Indoor, did you detect ten ants all time? What was the accuracy of the detection?

AR2.4: VisualMarkData is a marking tool with a graphical user interface, which can facilitate users to mark targets in the scene quickly. We marked the location and identity information of each ant in each frame, as described in lines 10-11 of the abstract. Thus, we didn’t use neural network to detect ants in this manuscript. Our contribution is to manually mark the trajectories of ants using VisualMarkData to obtain ground-truth. As a "Data Note" type article, the introduction to the dataset and its production method is our main contribution. As for the accuracy of the annotations, all the image sequences in the dataset were visually confirmed by a data annotator. More detailed information is described in the visual confirmation subsection of the revised manuscript (lines 165-185).

RC2.5: [Minor comments] Indoor environment: How many colonies did you collect? How did you record the video outdoor? I couldn't find this information in the Outdoor environment and Hardware devices for acquiring raw data.

AR2.5:
For indoor environment, we collected 50 workers of Japanese arched ant species and randomly divided them into 5 colonies of 10 ants each, as described in lines 99-105 in the revised manuscript. We have supplemented information such as video record of the outdoor environment and hardware devices for acquiring raw data in the revised manuscript (see details in lines 293-303 of the revised manuscript).

RC2.6: [Minor comments] Visually confirm: I am unsure of the meaning of poor quality and needed to be re-marked. Can you show the use before and after of re-marked image? In addition, did you annotate just an ant or keep the ID of an ant? If you keep an ant's ID, how did you do?

AC2.6: To make the meaning of mark quality and re-marking clearer and easier to understand, we add an example of re-marking a segment, as shown in Figure 4.

Besides, we always keep one ant's ID while marking. Specifically, the user only marks the same object until finishes the entire image sequence, and then the user can focus on another object and repeating the same operation. In order to help users quickly locate the same target in the next frame, the marked location on the previous frame will be displayed with a green-dotted. Above description can be seen in lines 333-343 of the revised manuscript.

Reviewer #3

RC3.0: Authors make available a new dataset of ant colony videos, containing scenes from 10 different ant colonies, with indoor and outdoor variants, to aid in the preparation of better computer vision approaches to track ants. Indeed, those methods require a good amount of labeled data, which is very time consuming and error prone in such insects. Videos seem to have a good variety of resolution and number of ants (crowding). They also provide an open-source tagging software to aid in the augmentation of such datasets. Code and data is publically available.

AR3.0: Thank you for your careful evaluation of this manuscript.

RC3.1: Given the details above, I don't have major concerns about this work. I would only recommend checking English in some sentences, but it is on average very well written.

AR3.1: We have made any necessary modifications and check that intended meaning has been retained. And here we did not list the changes but marked in blue (add) and red (delete) in revised paper. Please see the revised manuscript with track changes for details.

RC3.2: In addition, others are also working on improving the capture of such insects in natural environments, so it could be useful to cite them as well. One in particular provides open-hardware scheme to make it reproducible: Sabattini, J. A., Reta, J. M., Bugnon, L. A., Cerrudo, J. I., Sabattini, R. A., Peñalva, A., ... & Sturniolo, F. (2022). AntVideoRecord: Autonomous system to capture the locomotor activity of leafcutter ants. HardwareX, 11, e00270.

AR3.2: Thanks for the comments and suggestions. In the introduction (named context in the current version), we add two works on capturing insects in the natural environment and modify the relevant contents. The comparison before and after modification is as follows (see details in lines 38-44 of the revised manuscript):

Origin: "A critical requirement for the development of these models is access to the datasets containing motion trajectories of insects in the video. To the best of our knowledge, however, the current studies are all using only one outdoor-scene dataset, which is lack of data diversity."

Revision: "A critical requirement for the development of these models is access to the datasets containing motion trajectories of insects in the video. Several works are working on improving the capture of such insects in natural environments~\cite{imirzian2019automated, sabattini2022antvideorecord}. To the best of our knowledge, however, only a few works~\cite{imirzian2019automated, cao2020online} annotate motion trajectories in videos, and all use only a single outdoor scene sequence, which lacks data diversity."
