# Supplementary material for: A dataset of ant colonies’ motion trajectories in indoor and outdoor scenes to study clustering behavior
Source: Gigascience. 2022 Oct 28;11:giac096. doi: 10.1093/gigascience/giac096 (PMC9614923; doi:10.1093/gigascience/giac096)
Supplement: giac096_Response_to_Reviewer_Comments_Original_Submission [file giac096_response_to_reviewer_comments_original_submission.pdf]

Author's Response To Reviewer Comments

Close

We want to thank you for your valuable comments.
We submitted 3 files, including a revised manuscript, a track changes file (to highlight differences between the revised and the original manuscript), and this file, i.e., the response letter (a complete response to the editor and all reviewers). In the following, we respond to each of your concerns and recommendations.
(EC: Editor’s Comment, RC: Reviewer's Comment, AR: Authors' Response)

Editor
EC0: In particular, the reviewers mention the need to add more specific information and metadata to make the data set useful for others (including, for example, the scientific name of the ant species used and more exact details on data acquisition - see the reports below). In addition to the reviewers' reports, some minor editorial points from my side:
AR0: We thank the editor and reviewers for their kind comments and responded in a point-by-point manner.

EC1: We will consider the submission for publication as a "Data Note" - please check our instructions or authors for formatting guidelines for this article type (you do not need to change the text in a major way, but please try to use the section headings as indicated for the "Data Note" article type, if applicable). (Please also note that the Data Note article type has a lower Article Processing Charge than "Research".)
AR1: We modified the article to "Data Note" type by referring to the formatting guidelines on the journal website.

EC2: On page 2, you mention that data is available "subject to a royalty-free license", but at the end you provide another source which is public domain /cc0 (which is great). If the data is available under public domain, I recommend you remove the alternative source, or clarify if the data fulfills public domain criteria - (or are these different data sets? If you need help to make data available, we can also store it under a cc0 licence on our server GigaDB.)
AR2: We removed the alternative sources mentioned in the second page in the revised manuscript.

EC3: In the methods section, I recommend the use of past tense for the procedures that you carried out. ("We collected ants" instead of "we collect ants", etc..). Please also include the helpful wording suggestions from the reviewers' reports.
AR3: In the Data Description section and the Methods section of the revised manuscript, we have changed the procedures that are carried out to the past tense (see details in lines 96-124 and 274-303 of the revised manuscript).

EC4: In addition, please register the new software application in the bio.tools and SciCrunch.org databases to receive RRID (Research Resource Identification Initiative ID) and biotoolsID identifiers, and include these in your manuscript (in the code availability section). This will facilitate tracking, reproducibility and re-use of your tool.
AR4: We registered in bio.tools and SciCrunch.org respectively. The biotoolsID identifiers we obtained are ants_marking_and_analysis_tools and the RRID is SCR_022543. These information can be found in the Availability of source code and requirements section in lines 365-366 of the revised manuscript.

Reviewer reports:
Reviewer #1
RC1.0: Authors proposed a new software to evaluate ant movement, very useful to understand ant behavior and also to use their patterns to apply to other areas of science. I found a very interesting manuscript, well written and well addresses as well. This is a great advance in the methods to evaluate ant foraging, which I feel is useful for scientific researchers from many areas. I have some suggestions that I hope can help the authors strengthen the manuscript, making it clear to readers.
AR1.0: We thank the reviewer for the kind comments.

RC1.1: [Abstract] Lines 1,2: The clustering mechanism is most related to inside the colony than outside, in terms of the behavior of ant colonies that cluster their corpses and sort their larvae inside the colony. Or it could be valid for outside the colony like the authors made, at the entrance of the nest?
AR1.1: As you pointed out, clustering mechanism is most related to inside the colony, hence what we want to explore is the mechanism inside the colony. However, it seems that our statement is not clear enough. The so-called "indoor" and "outdoor" refer to the laboratory environment with a clean background (as shown in Figure 2a) and the natural environment (as shown in Figure 3a), respectively. We modified the abstract to eliminate ambiguity, as detailed in lines 2-3 of the revised manuscript, and shown below.
Origin: "Most studies in the field of ant behavior have only focused on indoor environments, while outdoor environments are still underexplored."
Revision: "Most studies in the field of ant behavior have only focused on indoor environments (laboratory setup), while outdoor environments (natural environments) are still underexplored."

RC1.2: [Abstract] Line 3: Include how many videos were taken indoors and outdoor.
AR1.2: We have 10 videos in total, 5 of which are indoor (laboratory setting) scene and 5 of which are outdoor (natural environment) scene. The comparison before and after modification is as follows (see details in lines 3-4 of the revised manuscript):
Origin: "In this paper, we collect 10 videos of ant colonies from different indoor and outdoor scenes."
Revision: "In this paper, for the first time, we collect 10 videos and 3 species of ant colonies from different scenes, including 5 indoor and 5 outdoor scenes."

RC1.3: [Abstract] Line 3: Maybe "And" instead of "In addition".
AR1.3: "In addition" has been changed to "And". The comparison before and after modification is as follows (see details in line 4 of the revised manuscript):
Origin: "In addition, we develop an image sequence marking software named VisualMarkData …"
Revision: "And we develop an image sequence marking software named VisualMarkData …"

RC1.4: [Abstract] Line 5: Maybe using the notation "114,112" instead of "114112".
AR1.4: "114112" has been changed to "114,112". The comparison before and after modification is as follows (see details in lines 10-11 of the revised manuscript):
Origin: "In all 5354 frames, the location information and the identification number of each ant are recorded for a total of 712 ants and 114112 annotations."
Revision: "In all 5,354 frames, the location information and the identification number of each ant are recorded for a total of 712 ants and 114,112 annotations."

RC1.5: [Introduction] Line 2 §2: The quality of what? From the information acquired?
AR1.5: To be more specific, we need to ensure the quality of the annotations, i.e., the accuracy. The comparison before and after modification is as follows (see details in lines 12-14 of the revised manuscript):
Origin: "Until the late 20th century, biologists still manually marked the motion trajectories on the video to guarantee the quality."
Revision: "Until the late 20th century, biologists still manually tracked the motion trajectories on the video to guarantee the accuracy of the marking."

RC1.6: [Introduction] Lines 5 §2: The final dot is after the reference: "[9].
AR1.6: The final dot has been placed after the reference. The comparison before and after modification is as follows (see details in line 16 of the revised manuscript):
Origin: ". [9]"
Revision: "[9]."

RC1.7: [Introduction] Lines 5,6 §2: I suggest removing the term "Obviously". Besides, the development of an automated tracking process is also time-consuming in relation to setting and calibrating the system (visual confirmation, etc.), right? And also, the automated tracking process can be more expensive than doing manually, right? If there is some previous work comparing the trade-off cost/benefit it could be included in the citation in order to better base the author's arguments.
AR1.7: "Obviously" has been removed. The comparison before and after modification is as follows (see details in line 16 of the revised manuscript):
Origin: "Obviously, manually tracking is time-consuming and prone to human error."
Revision: "Manually tracking is time-consuming and prone to human error."
Besides, the development of an automatic tracking process is indeed time-consuming. However, we only

need to develop it once. It is reusable for same tasks in the future. Once development finished, additional manually making time for every video will no longer be needed.

RC1.8: [Introduction] Line 1 §3: "release" instead of "free"?
AR1.8: "Free" has been modified to "release". The comparison before and after modification is as follows (see details in line 22 of the revised manuscript):
Origin: "Traditional CV techniques free researchers …"
Revision: "Traditional CV techniques release researchers …"

RC1.9: [Introduction] Lines 12, 13 §4: Regarding "only one outdoor scene dataset": Which one?
AR1.9: We want to express that only a single outdoor scene sequence is used in all the current studies. References to these studies have been added now. The comparison before and after modification is as follows (see details in lines 42-44 of the revised manuscript):
Origin: "To the best of our knowledge, however, the current studies are all using only one outdoor-scene dataset, which is lack of data diversity."
Revision: "To the best of our knowledge, however, only a few works~\cite{imirzian2019automated, cao2020online} annotate motion trajectories in videos, and both use only a single outdoor scene sequence, which lacks data diversity."

RC1.10: [Introduction] §4: I would suggest using "5,354" and "114,112" instead of "5354" and "114112".
AR1.10: We totally agree with your suggestion. "5354" and "114112" have been modified to "5,354" and "114,112". The comparison before and after modification is as follows (see details in lines 82-83 of the revised manuscript):
Origin: "The total size of our dataset is 5354 frames, 712 ants, and 114112 labels."
Revision: "The total size of the dataset is 5,354 frames, 712 ants, and 114,112 labels."

RC1.11: [Data Description] Line 2 §2: What is the time interval of each frame taken from the video?
AR1.11: In Table 1 of the revised manuscript, column 3 indicates the FPS (frames per second) of the video. Specifically, Seq0001~Seq0005 is 25 frames per second, the interval between two frames is 40 ms. Seq0006~Seq0010 is 30 frames per second, that is, the interval between two frames is 33 ms.

RC1.12: [Data acquisition] Line 1 §1: "ant" instead of "ants".
AR1.12: "ants" has been modified to "ant". The comparison before and after modification is as follows (see details in lines 99-100 of the revised manuscript):
Origin: "We collect 50 workers of Japanese arched ants species …".
Revision: "We collect 50 workers of Japanese arched ant species …".

RC1.13: [Data acquisition] Regarding the links, https://cn.depositphotos.com/home.html and https://data.mendeley.com/datasets/9ws98g4npw/3: How stable are those links? Does the journal require Dryad/Github or another repository?
AR1.13: We removed the first link because its information was already included in our dataset. For the second link, the Mendeley Data continues to provide open-source data repositories to researchers. They claim on their official website that the DOI of this dataset is available always: "To ensure the highest level of integrity and security possible , data is stored on Amazon's S3 servers in Ireland. Our service was extensively penetration tested and received certification. Additionally, your published datasets are archived with Data Archiving and Network Services (DANS) to preserve your data over the long term. DANS is a long-term archiving provider, which is an institute of the Dutch Academy KNAW, and of the Netherlands' national research council, NWO. We contract with DANS to archive all valid published datasets in perpetuity. The agreement ensures that the DOIs we provide for datasets will always resolve to a web page, where the dataset metadata and files will be available. Data archived at DANS is backed up and stored in three locations for redundancy."
Besides, the journal allows us to choose other repositories.

RC1.14: [Det folder] Provide the information you want to show and include the source between parenthesis. Do not start with the "The 'det' folder…"
AR1.14: Thank you for this suggestion, according to your suggestion, we modified the expression and the comparison before and after modification is as follows (see details in lines 134-139 of the revised manuscript):
Origin: "The 'det' folder contains a 'det.txt' file which is the ground truth for detection, recording the location parameters of the ants in all frames, which is similar to multi-object tracking challenge~\cite{leal2015motchallenge}. Each line represents one ant instance, and it contains 7 values

as shown in Table~\ref{tab:data_format}."
Revision: "In the same format as the dataset of multi-object tracking challenge~\cite{leal2015motchallenge}, we record information such as the identity and location parameters of all ants in each frame for detection. Such information is stored in a 'det.txt' file in a folder named 'det' in our dataset. Concretely, each line represents one ant instance, and it contains 7 values (also called attributes) as shown in Table~\ref{tab:data_format}."

RC1.15: [Det folder] Line 3: "which is similar to multi-object tracking challenge": And how is it? Similar how?
AR1.15: The multi-object tracking challenge~\cite{leal2015motchallenge} we describe here is a dataset, and the format of our dataset is the same as that. We modified the expression, and the comparison before and after modification is as follows (see details in lines 134-137 of the revised manuscript):
Origin: "The 'det' folder contains a 'det.txt' file which is the ground truth for detection, recording the location parameters of the ants in all frames, which is similar to multi-object tracking challenge~\cite{leal2015motchallenge}."
Revision: "In the same format as the dataset of multi-object tracking challenge~\cite{leal2015motchallenge}, we record information such as the identity and location parameters of all ants in each frame for detection. Such information is stored in a 'det.txt' file in a folder named 'det' in our dataset."
Besides, the specific format can be seen in lines 139-147 or Table 2 of the revised manuscript.

RC1.16: [Det folder] Line 4: "the position of one ant at one instant classified into seven categories (values 1-7)"? instead of "one ant instance and it contains 7 values"
AR1.16: We use 7 attributes to represent each ant instance, including frame number, identity number, bounding box left, bounding box top, bounding box width, bounding box height, and confidence score (see Table 2 for details). However, it seems that there was an ambiguity in our original statement, so we revised it. The comparison before and after modification is as follows (see details in lines 138-139 of the revised manuscript):
Origin: "Each line represents one ant instance, and it contains 7 values as shown in Table~\ref{tab:data_format}."
Revision: "Concretely, each line represents one ant instance, and it contains 7 values (also called attributes) as shown in Table~\ref{tab:data_format}."

RC1.17: [Gt folder] The same for Det folder. Describe the results, and the information, and then reference the source.
AR1.17: Thank you for this suggestion, according to your suggestion, we modified the expression, and the comparison before and after modification is as follows (see details in lines 149-153 of the revised manuscript):
Origin: "The 'gt' folder contains a 'gt.txt' file, which is the ground truth for muti-object tracking. Similar to the 'det.txt' file, it also contains 7 values (see details in Table~\ref{tab:data_format})."
Revision: "In our dataset, we provide groundtruth records for multi-object tracking. These information are stored in a 'gt.txt' file in a folder named 'gt'. Similar to the previous description of the 'det.txt' file, the records of each instance in the 'gt.txt' file also contains 7 values (also called attributes), see Table~\ref{tab:data_format} for details."

RC1.18: [Img folder] The same as the previous comment. Also, after "e.g." there is a comma: "e.g.,"
AR1.18: Thank you for this suggestion, according to your suggestion, we modified the expression and the comparison before and after modification is as follows (see details in lines 159-160 of the revised manuscript):
Origin: "The 'img' folder stores the original image sequence converted from the video."
Revision: "In our dataset, we provide the original image sequence converted from the video, which are stored in the 'img' folder."
Besides, "e.g.”has been modified to "e.g.,", The comparison before and after modification is as follows (see details in lines 161-162 of the revised manuscript):
Origin: "All images are converted to JPEG and named sequentially to a 6-digit file name (e.g. 000001.jpg)"
Revision: "All images are converted to JPEG and named sequentially to a 6-digit file name (e.g., 000001.jpg)"

RC1.19: [Analyses] Visually confirmation instead of "Visually confirm"
AR1.19: "Visually confirm" has been modified to "Visually confirmation". The comparison before and after modification is as follows (see details in line 164 of the revised manuscript):

Origin: "Visually confirm"
Revision: "Visually confirmation"

RC1.20: [Analyses] Please describe how many people did it, if it expected some bias depending on the person that check the images? Also: Is it necessary to do with all images? How much time does it take? Can you all estimate the labor time, is it?
AR1.20:
Q1: Please describe how many people did it, if it expected some bias depending on the person that check the images?
A1: We have 2 staff marking indoor videos and 3 staff marking outdoor videos. And there is only one inspector, so the bias is avoided. (See details in lines 165-168 of the revised manuscript)
Q2: Is it necessary to check all images?
A2: In the sequence-level checking, all sequences need to be checked. In the image-level checking, Only the sequence that is considered to be of low quality needs to have each frame checked. (See details in Figure 4 and lines 170-181 of the revised manuscript)
Q3: How much time does it take? Can you all estimate the labor time, is it?
A3: In the sequence-level checking, depending on how many ants are in each video, the time cost ranges from 8 to 10 times the length of the original video sequence. In the image-level checking, the check speed is about 0.5 seconds/ant, and the repair time is about 2 seconds/ant. (See details in lines 177-179 and 184-185 of the revised manuscript)
A comparison of the above three questions involving the content before and after the revision is as follows (see details in lines 165-185 of the revised manuscript):
Origin: "The ground truth annotations for all image sequences in the dataset were visually confirmed by the data annotation staff. The visual reviewing consists of two aspects, sequence-level (coarse-grained) and image-level (fine-grained). Firstly, staffs performed a coarse-grained review of a single sequence. Specifically, we drew the annotations on the corresponding images, and then converted the image sequence to video. By replaying the video, staff can quickly confirm which segments of the video are poor quality and needed to be re-marked. For each scene, an example image frame is shown in Figure~\ref{fig:tech_val_in} (a) and Figure~\ref{fig:tech_val_out} (a). After that, staff reviewed the quality of annotations frame-by-frame via VisualMarkData. For inaccurate annotations, staff modified manually by using the "Check and modify" function of VisualMarkData (see details in Methods)."
Revision: "In all 10 videos, we have 2 staff to mark indoor videos and 3 staff to mark outdoor videos. Furthermore, the ground-truth annotations for all image sequences in the dataset were visually confirmed by one staff. The visual reviewing consists of two aspects, sequence-level (coarse-grained) and image-level (fine-grained). Firstly, the staff performed a coarse-grained review of a single sequence. Specifically, we drew the annotations on the corresponding images, and then converted the image sequence to video. For each scene, an example image frame is shown in Figure~\ref{fig:tech_val_in} (a) and Figure~\ref{fig:tech_val_out} (a). By replaying the video, staff can quickly confirm which segments of the video are of poor quality and needed to be re-marked. Figure~\ref{fig:low_quality} (a) shows an example of a segment distinguished as low-quality annotations. The sequence-level checking time consumption per video is 8 to 10 times the original video sequence duration, which depends on the number of ants in the video. After that, staff reviewed the quality of annotations frame-by-frame via VisualMarkData. For inaccurate annotations, staff modified manually by using the "Check and modify" function of VisualMarkData (see details in Methods). Figure~\ref{fig:low_quality} (b) shows the modified annotations. The image-level checking speed is about 0.5 sec per ant instance while correction takes about 2 sec per ant instance."

RC1.21: [Analyses] After the equation 3: "is set to o": I guess this is an "o" and not a "0" (zero).
AR1.21: Here v0 is set to zero, because we assume that the ants are stationary at the initial moment. We added this assumption after the expression. The comparison before and after modification is as follows (see details in lines 187-188 of the revised manuscript):
Origin: "Where, the v_{0} is set to 0."
Revision: "Where, the v_{0} is set to 0, i.e. we assumed that the ants were stationary at the initial moment."

RC1.22: [Discussion] Lines 9-11 §1: Where are the Python Scripts available?
AR1.22: Links to the Python scripts have been added now. The comparison before and after modification is as follows (see details in lines 207-210 of the revised manuscript):
Origin: "Alongside, we have provided publicly available Python Scripts to illustrate the analysis of data as well as usage of the data."
Revision: "Alongside, we have provided publicly available Python Scripts at ~\url{https://github.com/holmescao/ANTS_marking_and_analysis_tools}, to illustrate the analysis of

data as well as usage of the data."

RC1.23: [Discussion] Line 15: "the" instead of "our".
AR1.23: “our” has been modified to “the”. The comparison before and after modification is as follows (see details in line 214 of the revised manuscript):
Origin: "… evaluate the tracking accuracy on our dataset."
Revision: "… evaluate the tracking accuracy on the dataset."

RC1.24: [Discussion] Lines 1-3 §2: How it would be made? When it would be available? I think that promising something in a paper is not appropriate. Maybe the authors can only tell something parsimonious like: "it is possible that some updates will be done in the future".
AR1.24: We highly agree with your suggestion, and we modified the sentence expression and the comparison before and after modification is as follows (see details in lines 218-220 of the revised manuscript):
Origin: "In the future, we will enrich the VisualMarkData with more features to reduce the difficulty of marking and improve the efficiency of marking."
Revision: "In the future, it is possible that VisualMarkData software will be made some updates to reduce the difficulty of marking and improve the efficiency of marking."

RC1.25: [Potential implications] It is a bit vague about the implications (and also applications). Please provide citations of previous articles that used the manual tracking and conclusions they made and mention how this technique can improve the reach of these studies in terms of investigating outdoor nests and also more replicates.
AR1.25: According to your suggestion, we modified the potential implications section (The current version is called Potential usage of dataset). The comparison before and after modification is as follows (see details in lines 237-270 of the revised manuscript):
Origin: "Swarm behavior is one of the most important features of social insects, which has important significance for the study of embodied intelligence~\cite{tiacharoen2012design}. Specifically, social insects often tend to cluster into a colony~\cite{vandermeer2008clusters}, which forms a complex dynamical system together with the surrounding environment~\cite{balch2001automatically}. So far, researchers do not know enough about the mechanisms behind swarm behaviors of social insects. We believe our image sequence marking software and dataset could facilitate the analysis of ant colony behavior leading to the development of embodied intelligence."
Revision: " Swarming behavior is one of the most important features of social insects~\cite{vandermeer2008clusters}, often involving division of labor~\cite{holldobler1990ants}, task specialization~\cite{whitehouse1996ant}, distributed problem solving~\cite{vaughan2000whistling}, etc. To reveal the mechanisms behind swarming behavior requires observing insect colonies over long periods of time as well as recording the motion trajectory of each individual~\cite{poff2012efficient}. Before the advance of computer vision technology, biologists utilize manual tracking to study insect behaviors~\cite{bond1980optimal, deffernez1990analysis}. Since manual recording is time-consuming and laborious, biologists focus only on individual behavioral studies, including foraging activity~\cite{deffernez1990analysis} and prey avoidance~\cite{bond1980optimal}, etc. In recent years, in order to enable rapid tracking of the activities of multiple insects simultaneously, automated image-based tracking techniques are employed, and a lot of attempts are made to improve the accuracy of tracking~\cite{khan2005mcmc, khan2006mcmc, oh2006parameterized, veeraraghavan2008shape, fletcher2011multiple}. These techniques have assisted biologists to discover some colony mechanisms. For example, Balch T et al~\cite{balch2001automatically} found that a number of ants would interact at the entrance of the nest when some find food nearby. However, current studies are almost limited to laboratory settings with clean backgrounds. Such approaches disregard the influence of the environment surrounding the insect colony, including potential predators~\cite{feener1990defense}, obstacles on the road~\cite{loreto2013foraging}, etc. In contrast, we provide labeled motion trajectories of ant colonies active outdoors, containing a variety of scenes. These data can be used to train deep learning models for automated tracking ants in natural environments. Moreover, we already used indoor/Japanese arched ants' images as the training set in our previous work~\cite{cao2020online} and tested our model on outdoor/black ant images (Seq0010), achieving a tracking accuracy up to 92\%. Vice versa, we also conducted experiments using outdoor images as the training set and indoor images as the test set, which are presented in a method manuscript that we are preparing~\cite{wu2022swarm}, which can be found at arXiv. Hence, it will help biologists to quantify and analyze the foraging patterns of ant colonies in natural environments, such as foraging strategies, partner gathering, and collaborative transportation."

RC1.26: [Methods] More information is required here (or can be placed in the Data Description section).

Which ant species do the ant colonies belong to? Which size (more or less) do the ant workers have? Outside or inside the nest? Close to the entrance? What angle of view was used, i.e., distance from ground? The height where the camera was placed? Which camera was used? Area of the frames? Description of the background? The background was cleaned prior to the recording? Etc.
AR1.26: We added the information you suggested and below are the answers to your questions.
Q1: Which ant species do the ant colonies belong to?
R1: Column 7 in the bottom part of Table 1, and lines 96 and 111 of the revised manuscript describe the species of ants of each video.
Q2: Which size (more or less) do the ant workers have?
R2: The ants in each scene are all worker ants, see lines 99-100 and 112-114 in the revised manuscript for details.
Q3: Outside or inside the nest?
R3: No nest in the indoor scenes. Outdoor scenes are filmed outside the nest except for Seq0010. See lines 103-104 and 121-122 in the revised manuscript for details.
Q4: Close to the entrance?
R4: Only the four video scenes of outdoor scenes are close to the entrance, including Seq0006 to Seq0009. See Table 1 and lines 121-122 of the revised manuscript for details.
Q5&Q6: What angle of view was used, i.e., distance from ground? The height where the camera was placed?
R5&A6: Table 1, Figure 5, and lines 281-285 and 297-301 of the revised manuscript describe the camera angle, the field of view of the camera, and the height of the camera from the ground for each video sequence.
Q7: Which camera was used?
A7: Table 1, lines 278-280 and 295-296 of the revised manuscript describe the camera types.
Q8：Area of the frames?
A8: Table 1 gives the area of each scene (length times width). Figure 5, Equation 4, and lines 285-290, 301-303 of the revised manuscript describe how the area is calculated.
Q9&Q10：Description of the background? The background was cleaned prior to the recording?
A9&A10: Lines 103-104 and 116-122 of the revised manuscript describe the backgrounds of scenes. Besides, we added information about the date, location, and temperature during the filming, as detailed in Table 1.

RC1.27: [Methods] Figure 1: Figure 1 was never referenced in the text. Line 4, Caption of Figure 1: Describe each of the three visualization tools to verify the data quality.
AR1.27: We have added the mention of Figure 1 in 2 places of the context. The added information is as follow (see details in lines 80-81 and 307-308 of the revised manuscript):
Add: "Then utilizing VisualMarkData and following the process shown in Figure~\ref{fig:pipeline}, …"
Add: "The overall annotation pipeline for the dataset using this software is shown in Figure~\ref{fig:pipeline}."
About caption of Figure 1, we added detail descriptions of three Python scripts. The comparison before and after modification is as follows (please see the description of Figure 1 lines 4-5):
Origin: "Additionally, we provide three visualization tools to verify the data quality."
Revision: "Additionally, three Python scripts are provided to generate three visualization results to verify the quality of the data, including the trajectories drawn on the original graph, the heat map of motion velocity, and the histogram of the frequency distribution of motion velocity."

RC1.28: [Methods] Figure 2: I suggest changing Velocity to Speed, prefer the scalar rather than the vector.
AR1.28: "Velocity" in Figure 2 has been modified to "Speed", as detailed in Figure 2.
Besides, all the "velocity" in the manuscript have been replaced by "speed".

RC1.29: [Methods] Line 2, Caption of Figure 2: "five consecutive sequences" instead of "5 sequences" and then remove ", respectively". "Histogram of the frequency of ant speeds in cm/s for indoor sequences" instead of "Ant speed histograms per indoor sequence".
AR1.29: We fully adopt your suggestions. The comparison before and after modification is as follows (see details in Figure 2 lines 1-2):
Origin: "(b) Speed distributions in image space for 5 sequences of indoor scenes, respectively. (c) Ant speed histograms per indoor sequence."
Revision: "(b) Speed distributions in image space for five consecutive sequences of indoor scenes. (c) Histogram of the frequency of ant speeds in cm/s for indoor sequences."

RC1.30: [Methods] Table 1: Describe the meaning of each column, Sequence is ...XXX, FPS is ...XXX,

etc. Maybe "statistics" is not the appropriate term; prefer the use of "description" or something like that.
AR1.30: We described the meaning of each column and changed "Statistics" to "Description". It should be noted that we expanded the column of Table 1 based on other comments. The information before and after the modification of caption is as follows (see details in Table 1):
Origin: "Statistics of ant videos with annotations in indoor and outdoor scenes."
Revision: "Description of ant videos with annotations in indoor and outdoor scenes. Top part provides filming details. Sequence = Name of video for each colony. Angle = Angle of the camera during filming. Height = Height of camera from the ground. Temp = Local temperature during filming. Datetime = Date and time of the filming. Location = Location of the filming. Camera = Camera type. Bottom part provides a description of ant videos with annotations. FPS = Frame rate of the video. Resolution = The resolution of the video. Length = The number of frames of the video, with the duration in parentheses. Ants = The number of ants with different IDs that appear in the video. Annotations = The number of ants instances labeled in the video. Species = The ant species. Entrance = Whether the colony is active at the nest entrance. Area = The area of the filmed scene. Note that the camera's angle of view is 16° and 7.5° in the horizontal and vertical directions, respectively, which are not represented in the table."

RC1.31: [Methods] Table 2: Maybe the files could be provided as an appendix for the paper.
AR1.31: Thank you for your suggestion. However, due to each file contains thousands of lines, it would be inconvenient to add the files directly to the manuscript's appendix. Thus, we published the files in https://data.mendeley.com/datasets/9ws98g4npw/3, which is convenient for users to view and download.

Reviewer #2
RC2.0: This paper developed the image sequence marking software VisualMarkData and tracked ants' locomotion in indoor and outdoor environments. All data of these images are available. Developing new tracking software is important for animal behaviour and computational biology. However, it wasn't easy to follow the novelty of this study and its importance as biological research.
AR2.0: We thank the reviewer for the kind comments.

RC2.1: [Major comments] What is the novel point of the image sequence marking software VisualMarkData? The authors have mentioned outdoor and indoor, but how did you overcome this difficulty?
AR2.1: For the first question, the main contribution of VisualMarkData is that it offers: (1) a comprehensive annotation of states at individual-target as well as group-target level; (2) representation of annotations (together with their IDs and behavior labels) of multiple targets and multiple groups in a simple-to-access matrix format; (3) a simple and efficient visualization during annotation, which presents the annotation information of the previous frame as a reference and then only requires clicking on the center point of each target to complete the annotation; and (4) a windows-based friendly graphical user interface that minimizes labor and maximizes annotation quality. We revised the abstract and the introduction (current version is context) about the VisualMarkData to highlight these contributions, as described in lines 4-10 and 62-74, respectively, of the revised manuscript.
In addition, since the type of our manuscript is "Data Note", we focus on providing a detailed approach to data production, validation, and potential reuse, which is in line with the instructions on the journal's website, see https://academic.oup.com/gigascience/ pages/data_note#Criteria. We are the first to construct the ant colony activity dataset with annotations with multiple species and colonies in both indoor and outdoor environments. Specifically, the dataset contains 3 species and 10 colonies with a total of 5,354 frames, 712 ants, and 114,112 labels.
Where the revision of the descriptions of the abstract is shown below (It can also be found in lines 4-10 of the revised manuscript abstract).
Origin: "And we develop an image sequence marking software named VisualMarkData, which enables us to provide annotations of ants in the video."
Revision: "And we develop an image sequence marking software named VisualMarkData, which enables us to provide annotations of ants in the video: (1) offers a comprehensive annotation of states at individual-target as well as colony-target level; (2) provides a simple matrix format to represent multiple targets and multiple groups of annotations (along with their IDs and behavior labels); (3) during the annotation process, we propose a simple and effective visualization that takes the annotation information of the previous frame as a reference and then simply clicks on the center point of each target to complete the annotation; and (4) we develop a user-friendly windows-based GUI to minimize labor and maximize annotation quality."
Where the revision of the descriptions of the introduction (called context in the current version) is shown below (it can also be found in lines 62-74 of the revised manuscript).

Origin: "Besides, we develop an image sequence mark software named VisualMarkData, which is used to mark the pixel patches covered by ants in each frame of the video."
Revision: "With respect to the tool, we propose the VisualMarkData, which allows users to generate ground-truth information of multi-target motion trajectories in video sequences. Specifically, VisualMarkData offers: (1) a comprehensive annotation of states at individual-target as well as group-target level; (2) representation of annotations (together with their IDs and behavior labels) of multiple targets and multiple groups in a simple-to-access matrix format; (3) a simple and efficient visualization during annotation, which presents the annotation information of the previous frame as a reference and then only requires clicking on the center point of each target to complete the annotation; and (4) a windows-based friendly graphical user interface that minimizes labor and maximizes annotation quality."
For the second question, there is no difference in the way we annotate the indoor and outdoor scene videos, and the detailed annotation method is described in lines 311-354 of the revised manuscript.
As for the method of data acquisition, we provide a detailed description in the Data Description section, including both indoor and outdoor (added in the current version) ways, which are described in lines 95-124 of the revised manuscript.

RC2.2: [Major comments] What is the purpose of providing your dataset for training or future research? If it is for training, the authors will show that training using indoor/Japanese arched ants' images can be useful for tracking outdoor/black ant images or vice versa. If authors want other researchers re-analyze these data for any reason, basic information is lacking. The species name is very important; the authors need to mention it. It is better to provide the temperature, date, time, and location of the colony when they took video in the outdoor environment.
AR2.2: The main purpose of providing this data set is to train automated models for biologists to better understand the clustering behavior of insects, as described in lines 6-9 of the revised version. In the meanwhile, our dataset can also be used for future research.
For training, we already used indoor/Japanese arched ants' images as the training set in our previous work~\cite{cao2020online} and tested our model on outdoor/black ant images, achieving a tracking accuracy up to 92%. Vice versa, we also conducted experiments using outdoor images as the training set and indoor images as the test set, which are presented in a method manuscript that we are preparing~\cite{wu2022swarm}, which can be found at arXiv. These details are added in lines 260-267 of the Potential implications (named Potential usage of dataset in the current version) section of the revised manuscript.
For future research, we added relevant information so that other researchers re-analyze these data for any reason. Additional information includes:
- Colony size
- Species name
- Nest exist or not in the scene
- Relationship between the scene and the entrance of the nest
- The background of the scene is processed or not
- The background description of the filming scene
- Temperature during the filming
- Filming date and time
- Filming location
- Camera type and parameter
- Camera angle
- Filming height of camera
- The field of view of camera
- The area of the filming scene and its measure method

This information is supplemented in Table 1, Figure 5, Equation 4, lines 95-124, 277-290 and 295-303 of the revised manuscript.

RC2.3: [Major comments] Why did you use two species? Did you collect two species of ants and record their behaviour under both indoor and outdoor conditions? If not, why? Can we use data of different ant species as training data? For example, if you use data of Japanese arched ants as training, can you detect the black ant?
AR2.3: We use three species, including Japanese arched ants (also called Camponotus japonicus ants)~\cite{nakanishi2009sex, he2011bacteria, nishikawa2012higher}, Little black ants~\cite{wang2015fatty, thompson2019ants} and Carpenter ants~\cite{sanders1964biology, carney1969behavioral, carlin1986kin}, which are widely studied by behavioral ecologists and social biologists. Since Japanese arched ants are often domesticated, they are suitable for observation in laboratory environments. As for the other two species of ants, we only record their behavior outdoors and do not catch them in laboratory environments so as not to disturb their normal life. It is in

accordance with academic ethics that ant citizens should not be violated casually to prevent ecological damage. In the revised manuscript, the species of ants in each video are shown in Table 1, and the relevant descriptions can be found in lines 96-99 and 111-114.
In addition, we can use data of different ant species as training data. we already used indoor/Japanese arched ants' images as the training set in our previous work~\cite{cao2020online} and tested our model on outdoor/black ant images, achieving a tracking accuracy up to 92%. Vice versa, we also conducted experiments using outdoor images as the training set and indoor images as the test set, which are presented in a method manuscript that we are preparing~\cite{wu2022swarm}, which can be found at arXiv. These details are added in lines 260-267 of the Potential implications (named Potential usage of dataset in the current version) section of the revised manuscript.

RC2.4: [Minor comments] Indoor, did you detect ten ants all time? What was the accuracy of the detection?
AR2.4: VisualMarkData is a marking tool with a graphical user interface, which can facilitate users to mark targets in the scene quickly. We marked the location and identity information of each ant in each frame, as described in lines 10-11 of the abstract. Thus, we didn't use neural network to detect ants in this manuscript. Our contribution is to manually mark the trajectories of ants using VisualMarkData to obtain ground-truth. As a "Data Note" type article, the introduction to the dataset and its production method is our main contribution. As for the accuracy of the annotations, all the image sequences in the dataset were visually confirmed by a data annotator. More detailed information is described in the visual confirmation subsection of the revised manuscript (lines 165-185).

RC2.5: [Minor comments] Indoor environment: How many colonies did you collect? How did you record the video outdoor? I couldn't find this information in the Outdoor environment and Hardware devices for acquiring raw data.
AR2.5: For indoor environment, we collected 50 workers of Japanese arched ant species and randomly divided them into 5 colonies of 10 ants each, as described in lines 99-105 in the revised manuscript. We have supplemented information such as video record of the outdoor environment and hardware devices for acquiring raw data in the revised manuscript (see details in lines 293-303 of the revised manuscript).

RC2.6: [Minor comments] Visually confirm: I am unsure of the meaning of poor quality and needed to be re-marked. Can you show the use before and after of re-marked image? In addition, did you annotate just an ant or keep the ID of an ant? If you keep an ant's ID, how did you do?
AC2.6: To make the meaning of mark quality and re-marking clearer and easier to understand, we add an example of re-marking a segment, as shown in Figure 4.
Besides, we always keep one ant's ID while marking. Specifically, the user only marks the same object until finishes the entire image sequence, and then the user can focus on another object and repeating the same operation. In order to help users quickly locate the same target in the next frame, the marked location on the previous frame will be displayed with a green-dotted. Above description can be seen in lines 333-343 of the revised manuscript.

Reviewer #3
RC3.0: Authors make available a new dataset of ant colony videos, containing scenes from 10 different ant colonies, with indoor and outdoor variants, to aid in the preparation of better computer vision approaches to track ants. Indeed, those methods require a good amount of labeled data, which is very time consuming and error prone in such insects. Videos seem to have a good variety of resolution and number of ants (crowding). They also provide an open-source tagging software to aid in the augmentation of such datasets. Code and data is publically available.
AR3.0: Thank you for your careful evaluation of this manuscript.

RC3.1: Given the details above, I don't have major concerns about this work. I would only recommend checking English in some sentences, but it is on average very well written.
AR3.1: We have made any necessary modifications and check that intended meaning has been retained. And here we did not list the changes but marked in blue (add) and red (delete) in revised paper. Please see the revised manuscript with track changes for details.

RC3.2: In addition, others are also working on improving the capture of such insects in natural environments, so it could be useful to cite them as well. One in particular provides open-hardware scheme to make it reproducible: Sabattini, J. A., Reta, J. M., Bugnon, L. A., Cerrudo, J. I., Sabattini, R. A., Peñalva, A., ... & Sturniolo, F. (2022). AntVideoRecord: Autonomous system to capture the locomotor activity of leafcutter ants. HardwareX, 11, e00270.

AR3.2: Thanks for the comments and suggestions. In the introduction (named context in the current version), we add two works on capturing insects in the natural environment and modify the relevant contents. The comparison before and after modification is as follows (see details in lines 38-44 of the revised manuscript):
Origin: "A critical requirement for the development of these models is access to the datasets containing motion trajectories of insects in the video. To the best of our knowledge, however, the current studies are all using only one outdoor-scene dataset, which is lack of data diversity."
Revision: "A critical requirement for the development of these models is access to the datasets containing motion trajectories of insects in the video. Several works are working on improving the capture of such insects in natural environments~\cite{imirzian2019automated, sabattini2022antvideorecord}. To the best of our knowledge, however, only a few works~\cite{imirzian2019automated, cao2020online} annotate motion trajectories in videos, and all use only a single outdoor scene sequence, which lacks data diversity."

Close
