# Supplementary material for: A dataset of ant colonies’ motion trajectories in indoor and outdoor scenes to study clustering behavior
Source: Gigascience. 2022 Oct 28;11:giac096. doi: 10.1093/gigascience/giac096 (PMC9614923; doi:10.1093/gigascience/giac096)
Supplement: giac096_Response_to_Reviewer_Comments_Revision_1 [file giac096_response_to_reviewer_comments_revision_1.pdf]

Author's Response To Reviewer Comments

Close

Response Letter to GigaScience Submission
Paper ID: GIGA-D-22-00055_R2
Paper Title: A dataset of ant colonies motion trajectories in indoor and outdoor scenes to study clustering behavior

We want to thank you for your valuable comments.
We submitted 4 files, including a revised manuscript, a track changes file (to highlight differences between the revised and the original manuscript), a certificate (verify that our revised manuscript was edited by Springer Nature Author Services) and this file, i.e., the response letter (a complete response to the editor). In the following, we respond to each of your concerns and recommendations.
(EC: Editor’s Comment, AR: Authors' Response)

Response to the Editor
EC0: Your revised manuscript "A dataset of ant colonies motion trajectories in indoor and outdoor scenes for social cluster behavior study" (GIGA-D-22-00055R1) is acceptable for publication as a Data Note in GigaScience, in principle. Before we proceed, our data curators will have another look at code and data and they may prepare an archival snapshot for our repository GigaDB. They will contact you shortly with any requests they might have. Please also address the following editorial points:
AR0: We thank the editor for your kind comments and responded in a point-by-point manner.

EC1: Language improvement. Maybe a native English speaker in your scientific community could go over the manuscript to correct some language issues. Overall the paper reads good, but some phrases are hard to understand because of grammar issues.
(e.g. line 164 "Visually confirmation" should be "visual confirmation"; line 218, "In the future, it is possible that VisualMarkData software will be made some updates" - ? should that read "will be updated"? ... and many other, similar small inaccuracies.
AR1: We fully adopt your suggestions, and according to your suggestions, we have tried to address some grammatical issues to improve the clarity of the manuscript and thus make it easier to understand. Firstly, we corrected the two specific grammatical problems you pointed out. The comparison before and after modification is as follows (see details in lines 165 and 233-234 of the revised manuscript):
Origin1: "Visually confirmation"
Revision1: " Visual confirmation "
Origin2: "In the future, it is possible that VisualMarkData software will be made some updates…"
Revision2: "In the future, it is possible that the VisualMarkData software will be updated…"
In addition, we submitted the manuscript to Springer Nature Author Services, a polishing agency. The editors thoroughly polished the grammar, punctuation, spelling, and overall style of our manuscript and performed multiple edits to further improve the flow and readability of the manuscript. The agency provided a certificate of polishing to verify that we have used this service. The certificate was issued on September 2, 2022, and can be verified on the SNAS website (secure.authorservices.springernature.com/certificate/verify) using the verification code 7258-D681-EA37-A41D-3ACP. We also attach the certificate to verify that our revised manuscript was edited by Springer Nature Author Services.

EC2: Title: ".. for social cluster behavior study" : this sounds a bit odd to my ears. Maybe better "to study clustering behavior" , or similar?
AR2: Thank you for your suggestion, according to your suggestion, the title ".. for social cluster behavior study" has been changed to "to study clustering behavior". The comparison before and after modification is as follows (see details in the title of the revised manuscript):
Origin: "A dataset of ant colonies motion trajectories in indoor and outdoor scenes for social cluster behavior study"
Revision: "A dataset of ant colonies motion trajectories in indoor and outdoor scenes to study clustering behavior"

EC3: Data citation:
In the data availability section, please move the doi for the video data as an item to the reference section and cite it by number, rather than directly mentioning the doi in the main text.
The citation is:

[xx] Cao, X (2022), "ANTS--ant detection and tracking", Mendeley Data, V4, doi: 10.17632/9ws98g4npw.4
Please cite the upcoming GigaDB repository the same way (i.e. by number from the availability section), if our curators prepare one (you'll get the citation details soon)
AR3: We adopt your suggestion. We moved the DOIs that appears twice in the text to the reference section and cited them in the text. The comparison before and after modification is as follows (see details in lines 127-129 and 384-385 of the revised manuscript):
Origin1: "The dataset consists of 10 image sequences from different scenes in JPEG digital image format, which is publicly available on
\url{https://data.mendeley.com/datasets/9ws98g4npw/3}."
Revision1: "The dataset consists of 10 image sequences from different scenes in JPEG digital image format, which is published in the ANTS--ant detection and tracking repository~\cite{cao2022ants, wu2022ants}."
Origin2: "The dataset supporting the results of this article is available in the ANTS--ant detection and tracking repository at \url{http://dx.doi.org/10.17632/9ws98g4npw.4}."
Revision2: "The dataset supporting the results of this paper is published in the ANTS--ant detection and tracking repository~\cite{cao2022ants, wu2022ants}."
The citations are:
[42] Cao X. ANTS–ant detection and tracking. Mendeley Data 2022;Doi:http://dx.doi.org/10.17632/9ws98g4npw.4.
[43] Wu M, Cao X, Yang M, Cao X, Guo S. Supporting data for "A dataset of ant colonies motion trajectories in indoor and outdoor scenes to study clustering behavior". GigaScience Database 2022;Doi:http://dx.doi.org/10.5524/102254.

EC4: Scientific names: When referring to the three ant species at first mention in the data description or methods section, please use the following format: common English name (Latin Binomial in italics; NCBI taxon ID): example: "Japanese ant (Camponotus japonicus; NCBI:txid84547)"
... and the same for the other two species.
AR4: As you suggested, we added NCBI taxon IDs when first mentioning the 3 ant species. he comparison before and after modification is as follows (see details in the lines 95-96 and 111-112 of the revised manuscript):
Origin1: "Japanese arched ants (also called Camponotus japonicus ant) are widely studied …"
Revision1: "Japanese arched ants (Camponotus japonicus; NCBI:txid84547) are widely studied …"
Origin2: "Little black ants~\cite{wang2015fatty, thompson2019ants} and Carpenter ants …"
Revision2: "Little black ants (Solenopsis invicta; NCBI:txid13686)~\cite{wang2015fatty, thompson2019ants} and Carpenter ants (Camponotus herculeanus; NCBI:txid36169) …"

EC5: Orcids: Please add the list of ORCIDS to the title page (we have this on our file: ORCIDs: Meihong Wu [0000-0002-1489-4039]; Xiaoyan Cao [0000-0002-4980-2050]; Xiaoyu Cao [0000-0003-0219-1798]; Shihui Guo [];)
AR5: We have added your list of ORCIDS to the title page. See the revised title page for details.

EC6: structured abstract: Please structure your abstract into three sections, titled "Background", "Findings" , "Conclusions"
AR6: According to your suggestion, we structured the abstract into 3 sections titled "Background", "Findings" and "conclusions". The comparison before and after modification is as follows (see details in the abstract of the revised manuscript):
Origin: "Motion and interaction of social insects (such as ants) have been studied by many researchers to understand the clustering mechanism. Most studies in the field of ant behavior have only focused on indoor environments (laboratory setup), while outdoor environments (natural environments) are still underexplored. In this paper, for the first time, we collect 10 videos and 3 species of ant colonies from different scenes, including 5 indoor and 5 outdoor scenes. And we develop an image sequence marking software named VisualMarkData, which enables us to provide annotations of ants in the video: (1) offers a comprehensive annotation of states at individual-target as well as colony-target levels; (2) provides a simple matrix format to represent multiple targets and multiple groups of annotations (along with their IDs and behavior labels); (3) during the annotation process, we propose a simple and effective visualization that takes the annotation information of the previous frame as a reference and then simply clicks on the center point of each target to complete the annotation; and (4) we develop a user-friendly windows-based GUI to minimize labor and maximize annotation quality. In all 5,354 frames, the location information and the identification number of each ant are recorded for a total of 712 ants and 114,112 annotations. Moreover, we provide visual analysis tools to assess and validate the technical quality and reproducibility of our data. It is hoped that this dataset will contribute to a deeper exploration of the behavior of the ant colony."
Revision: "\subsection{Background}
The motion and interaction of social insects (such as ants) have been studied by many researchers to understand clustering mechanisms. Most studies in the field of ant behavior have only focused on indoor environments (a laboratory setup), while outdoor environments (natural environments) are still underexplored.
\subsection{Findings}
In this paper, we collect 10 videos of 3 species of ant colonies from different scenes, including 5 indoor and 5

outdoor scenes. We develop an image sequence marking software named VisualMarkData, which enables us to provide annotations of the ants in the videos. (1) It offers comprehensive annotations of states at the individual-target and colony-target levels. (2) It provides a simple matrix format to represent multiple targets and multiple groups of annotations (along with their IDs and behavior labels). (3) During the annotation process, we propose a simple and effective visualization that takes the annotation information of the previous frame as a reference, and then a user can simply click on the center point of each target to complete the annotation task. (4) We develop a user-friendly window-based GUI to minimize labor and maximize annotation quality. In all 5,354 frames, the location information and the identification number of each ant are recorded for a total of 712 ants and 114,112 annotations. Moreover, we provide visual analysis tools to assess and validate the technical quality and reproducibility of our data.
\subsection{Conclusions}
We provide a large-scale ant dataset with the accompanying annotation software. It is hoped that our work will contribute to a deeper exploration of the behavior of ant colonies."

EC7: abstract. In the abstract of the revised manuscript, you write, "..., for the first time, we collect 10 videos a..." . I feel this is misleading, as it may give the wrong impression that this is the first data set of this kind. Please delete "for the first time" from the abstract - the novel contribution of your work is clear without this phrase.
AR7: Thank you for your suggestion, according to your suggestion, we delete "for the first time" from the abstract. The comparison before and after modification is as follows (see details in line 1 of the findings of the abstract of the revised manuscript):
Origin: "In this paper, for the first time, we collect 10 videos…"
Revision: "In this paper, we collect 10 videos …"

Close
