# Supplementary material for: A dataset of ant colonies’ motion trajectories in indoor and outdoor scenes to study clustering behavior
Source: Gigascience. 2022 Oct 28;11:giac096. doi: 10.1093/gigascience/giac096 (PMC9614923; doi:10.1093/gigascience/giac096)
Supplement: giac096_Response_to_Reviewer_Comments_Revision_2 [file giac096_response_to_reviewer_comments_revision_2.pdf]

Author's Response To Reviewer Comments

Close

Response Letter to GigaScience Submission
Paper ID: GIGA-D-22-00055_R3
Paper Title: A dataset of ant colonies motion trajectories in indoor and outdoor scenes to study clustering behavior

We want to thank you for your valuable comments.
We submitted 3 files, including a revised manuscript, a track changes file (to highlight differences between the revised and the original manuscript), and this file, i.e., the response letter (a complete response to the editor). In the following, we respond to each of your concerns and recommendations.
(EC: Editor's Comment, AR: Authors' Response)

Response to the Editor
EC1: The current data availability section reads:
"The dataset supporting the results of this paper is published in the ANTS–ant detection and tracking repository [42, 43]. Note that the files associated with this dataset are licensed under a Public Domain Dedication license."
Two small requests here:
1) The licence you used is called "cc0" Although this is in all practical terms identical to a Public Domain dedication, I think you should use the exact term.
2) I'd suggest to mention GigaDB separately.
So the paragraph could read:
"The dataset supporting the results of this paper is published in the ANTS–ant detection and tracking repository [42] and the GigaScience database GigaDB [43]. The files associated with this dataset are licensed under a cc0 licence, dedicating them to the public domain".
AR1: We fully adopt your suggestions, and according to your suggestions, we changed the manuscript accordingly. The comparison before and after modification is as follows (see details in lines 384-388 of the revised manuscript):
Origin: " The dataset supporting the results of this paper is published in the ANTS--ant detection and tracking repository~\cite{cao2022ants, wu2022ants}. Note that the files associated with this dataset are licensed under a Public Domain Dedication license."
Revision: "The dataset supporting the results of this paper is published in the ANTS–ant detection and tracking repository~\cite{cao2022ants} and the GigaScience database GigaDB~\cite{wu2022ants}. The files associated with this dataset are licensed under a cc0 license, dedicating them to the public domain."

Close
