# Supplementary material for: A dataset of ant colonies’ motion trajectories in indoor and outdoor scenes to study clustering behavior
Source: Gigascience. 2022 Oct 28;11:giac096. doi: 10.1093/gigascience/giac096 (PMC9614923; doi:10.1093/gigascience/giac096)
Supplement: giac096_Reviewer_1_Report_Original_Submission — Reuber Antoniazzi -- 4/8/2022 Reviewed [file giac096_reviewer_1_report_original_submission.pdf]

Reviewer Report

Title: A dataset of ant colonies motion trajectories in indoor and outdoor scenes to study clustering behavior

Version: Original Submission **Date:** 4/8/2022

Reviewer name: Reuber Antoniazzi

Reviewer Comments to Author:

Authors proposed a new software to evaluate ant movement, very useful to understand ant behavior and also to use their patterns to apply to other areas of science. I found a very interesting manuscript, well written and well addresses as well. This is a great advance in the methods to evaluate ant foraging, which I feel is useful for scientific researchers from many areas.
I have some suggestions that I hope can help the authors strengthen the manuscript, making it clear to readers.
Abstract
Lines 1,2: The clustering mechanism is most related to inside the colony than outside, in terms of the behavior of ant colonies that cluster their corpses and sort their larvae inside the colony. Or it could be valid for outside the colony like the authors made, at the entrance of the nest?
Line 3: Include how many videos were taken indoors and outdoor.
Line 3: Maybe "And" instead of "In addition".
Line 5: Maybe using the notation "114,112" instead of "114112".
Introduction
Line 2 Â§2: The quality of what? From the information acquired?
Lines 5 Â§2: The final dot is after the reference: "[9]."
Lines 5,6 Â§2: I suggest removing the term "Obviously". Besides, the development of an automated tracking process is also time-consuming in relation to setting and calibrating the system (visual confirmation, etc.), right? And also, the automated tracking process can be more expensive than doing manually, right? If there is some previous work comparing the trade-off cost/benefit it could be included in the citation in order to better base the author's arguments.
Line 1 Â§3: "release" instead of "free"?
Lines 12, 13 Â§4: Regarding "only one outdoor scene dataset": Which one?
Â§4: I would suggest using "5,354" and "114,112" instead of "5354" and "114112"
Data Description
Line 2 Â§2: What is the time interval of each frame taken from the video?
Data acquisition
Indoor environment
Line 1 Â§1: "ant" instead of "ants".
Regarding the links, https://cn.depositphotos.com/home.html and https://data.mendeley.com/datasets/9ws98g4npw/3: How stable are those links? Does the journal require Dryad/Github or another repository?

Det folder
Provide the information you want to show and include the source between parenthesis. Do not start with the "The 'det' folder…"
Line 3: "which is similar to multi-object tracking challenge": And how is it? Similar how?
Line 4: "the position of one ant at one instant classified into seven categories (values 1-7)"? instead of "one ant instance and it contains 7 values"
Gt folder
The same for Det folder. Describe the results, and the information, and then reference the source.
Img folder
The same as the previous comment. Also, after "e.g." there is a comma: "e.g.,"
Analyses
Visually confirmation instead of "Visually confirm"
Please describe how many people did it, if it expected some bias depending on the person that check the images? Also: Is it necessary to do with all images? How much time does it take? Can you all estimate the labor time, is it?
After the equation 3: "is set to o": I guess this is an "o" and not a "0" (zero).
Discussion
Lines 9-11 Â§1: Where are the Python Scripts available?
Line 15: "the" instead of "our".
Lines 1-3 Â§2: How it would be made? When it would be available? I think that promising something in a paper is not appropriate. Maybe the authors can only tell something parsimonious like: "it is possible that some updates will be done in the future"
Potential implications
It is a bit vague about the implications (and also applications). Please provide citations of previous articles that used the manual tracking and conclusions they made and mention how this technique can improve the reach of these studies in terms of investigating outdoor nests and also more replicates.
Methods
More information is required here (or can be placed in the Data Description section). Which ant species do the ant colonies belong to? Which size (more or less) do the ant workers have? Outside or inside the nest? Close to the entrance? What angle of view was used, i.e., distance from ground? The height where the camera was placed? Which camera was used? Area of the frames? Description of the background? The background was cleaned prior to the recording? Etc.
Figure 1: Figure 1 was never referenced in the text. Line 4, Caption of Figure 1: Describe each of the three visualization tools to verify the data quality.
Figure 2: I suggest changing Velocity to Speed, prefer the scalar rather than the vector. Line 2, Caption of Figure 2: "five consecutive sequences" instead of "5 sequences" and then remove ", respectively". "Histogram of the frequency of ant speeds in cm/s for indoor sequences" instead of "Ant speed histograms per indoor sequence".
Table 1: Describe the meaning of each column, Sequence is ...XXX, FPS is ...XXX, etc. Maybe "statistics" is not the appropriate term; prefer the use of "description" or something like that.
Table 2: Maybe the files could be provided as an appendix for the paper.

Level of Interest

Please indicate how interesting you found the manuscript: Choose an item.

Quality of Written English

Please indicate the quality of language in the manuscript: Choose an item.

Declaration of Competing Interests

Please complete a declaration of competing interests, considering the following questions:

- Have you in the past five years received reimbursements, fees, funding, or salary from an organisation that may in any way gain or lose financially from the publication of this manuscript, either now or in the future?
- Do you hold any stocks or shares in an organisation that may in any way gain or lose financially from the publication of this manuscript, either now or in the future?
- Do you hold or are you currently applying for any patents relating to the content of the manuscript?
- Have you received reimbursements, fees, funding, or salary from an organization that holds or has applied for patents relating to the content of the manuscript?
- Do you have any other financial competing interests?
- Do you have any non-financial competing interests in relation to this paper?

If you can answer no to all of the above, write 'I declare that I have no competing interests' below. If your reply is yes to any, please give details below.

I declare that I have no competing interests.

I agree to the open peer review policy of the journal. I understand that my name will be included on my report to the authors and, if the manuscript is accepted for publication, my named report including any attachments I upload will be posted on the website along with the authors' responses. I agree for my report to be made available under an Open Access Creative Commons CC-BY license (http://creativecommons.org/licenses/by/4.0/). I understand that any comments which I do not wish to be included in my named report can be included as confidential comments to the editors, which will not be published.

Choose an item.

To further support our reviewers, we have joined with Publons, where you can gain additional credit to further highlight your hard work (see: https://publons.com/journal/530/gigascience). On publication of

this paper, your review will be automatically added to Publons, you can then choose whether or not to claim your Publons credit. I understand this statement.

Yes Choose an item.
