# Supplementary material for: A dataset of ant colonies’ motion trajectories in indoor and outdoor scenes to study clustering behavior
Source: Gigascience. 2022 Oct 28;11:giac096. doi: 10.1093/gigascience/giac096 (PMC9614923; doi:10.1093/gigascience/giac096)
Supplement: giac096_Reviewer_2_Report_Original_Submission — HARUNA Fujioka -- 4/20/2022 Reviewed [file giac096_reviewer_2_report_original_submission.pdf]

Reviewer Report

Title: A dataset of ant colonies motion trajectories in indoor and outdoor scenes to study clustering behavior

Version: Original Submission **Date:** 4/20/2022

Reviewer name: HARUNA Fujioka

Reviewer Comments to Author:

This paper developed the image sequence marking software VisualMarkData and tracked ants' locomotion in indoor and outdoor environments. All data of these images are available. Developing new tracking software is important for animal behaviour and computational biology. However, it wasn't easy to follow the novelty of this study and its importance as biological research.

Major comments

1. What is the novel point of the image sequence marking software VisualMarkData? The authors have mentioned outdoor and indoor, but how did you overcome this difficulty?
2. What is the purpose of providing your dataset for training or future research? If it is for training, the authors will show that training using indoor/Japanese arched ants' images can be useful for tracking outdoor/black ant images or vice versa. If authors want other researchers re-analyze these data for any reason, basic information is lacking. The species name is very important; the authors need to mention it. It is better to provide the temperature, date, time, and location of the colony when they took video in the outdoor environment.
3. Why did you use two species? Did you collect two species of ants and record their behaviour under both indoor and outdoor conditions? If not, why? Can we use data of different ant species as training data? For example, if you use data of Japanese arched ants as training, can you detect the black ant?

Minor comments

1. Indoor, did you detect ten ants all time? What was the accuracy of the detection?
2. Indoor environment: How many colonies did you collect? How did you record the video outdoor? I couldn't find this information in the Outdoor environment and Hardware devices for acquiring raw data.
3. Visually confirm: I am unsure of the meaning of poor quality and needed to be re-marked. Can you show the use before and after of re-marked image? In addition, did you annotate just an ant or keep the ID of an ant? If you keep an ant's ID, how did you do?

Level of Interest

Please indicate how interesting you found the manuscript: Choose an item.

Quality of Written English

Please indicate the quality of language in the manuscript: Choose an item.

Declaration of Competing Interests

Please complete a declaration of competing interests, considering the following questions:

- Have you in the past five years received reimbursements, fees, funding, or salary from an organisation that may in any way gain or lose financially from the publication of this manuscript, either now or in the future?
- Do you hold any stocks or shares in an organisation that may in any way gain or lose financially from the publication of this manuscript, either now or in the future?
- Do you hold or are you currently applying for any patents relating to the content of the manuscript?
- Have you received reimbursements, fees, funding, or salary from an organization that holds or has applied for patents relating to the content of the manuscript?
- Do you have any other financial competing interests?
- Do you have any non-financial competing interests in relation to this paper?

If you can answer no to all of the above, write 'I declare that I have no competing interests' below. If your reply is yes to any, please give details below.

I declare that I have no competing interests.

I agree to the open peer review policy of the journal. I understand that my name will be included on my report to the authors and, if the manuscript is accepted for publication, my named report including any attachments I upload will be posted on the website along with the authors' responses. I agree for my report to be made available under an Open Access Creative Commons CC-BY license (http://creativecommons.org/licenses/by/4.0/). I understand that any comments which I do not wish to be included in my named report can be included as confidential comments to the editors, which will not be published.

Choose an item.

To further support our reviewers, we have joined with Publons, where you can gain additional credit to further highlight your hard work (see: https://publons.com/journal/530/gigascience). On publication of this paper, your review will be automatically added to Publons, you can then choose whether or not to claim your Publons credit. I understand this statement.

Yes Choose an item.
