# Supplementary material for: A dataset of ant colonies’ motion trajectories in indoor and outdoor scenes to study clustering behavior
Source: Gigascience. 2022 Oct 28;11:giac096. doi: 10.1093/gigascience/giac096 (PMC9614923; doi:10.1093/gigascience/giac096)
Supplement: giac096_Reviewer_3_Report_Original_Submission — Leandro Bugnon -- 4/25/2022 Reviewed [file giac096_reviewer_3_report_original_submission.pdf]

Reviewer Report

Title: A dataset of ant colonies motion trajectories in indoor and outdoor scenes to study clustering behavior

Version: Original Submission **Date:** 4/25/2022

Reviewer name: Leandro Bugnon

Reviewer Comments to Author:

Authors make available a new dataset of ant colony videos, containing scenes from 10 different ant colonies, with indoor and outdoor variants, to aid in the preparation of better computer vision approaches to track ants. Indeed, those methods require a good amount of labeled data, which is very time consuming and error prone in such insects. Videos seem to have a good variety of resolution and number of ants (crowding). They also provide an open-source tagging software to aid in the augmentation of such datasets. Code and data is publically available.
Given the details above, I don't have major concerns about this work. I would only recommend checking English in some sentences, but it is on average very well written.
In addition, others are also working on improving the capture of such insects in natural environments, so it could be useful to cite them as well. One in particular provides open-hardware scheme to make it reproducible:
Sabattini, J. A., Reta, J. M., Bugnon, L. A., Cerrudo, J. I., Sabattini, R. A., PeÃ±alva, A., ... & Sturniolo, F. (2022). AntVideoRecord: Autonomous system to capture the locomotor activity of leafcutter ants. HardwareX, 11, e00270.

Level of Interest

Please indicate how interesting you found the manuscript: Choose an item.

Quality of Written English

Please indicate the quality of language in the manuscript: Choose an item.

Declaration of Competing Interests

Please complete a declaration of competing interests, considering the following questions:

- Have you in the past five years received reimbursements, fees, funding, or salary from an organisation that may in any way gain or lose financially from the publication of this manuscript, either now or in the future?
- Do you hold any stocks or shares in an organisation that may in any way gain or lose financially from the publication of this manuscript, either now or in the future?

- Do you hold or are you currently applying for any patents relating to the content of the manuscript?
- Have you received reimbursements, fees, funding, or salary from an organization that holds or has applied for patents relating to the content of the manuscript?
- Do you have any other financial competing interests?
- Do you have any non-financial competing interests in relation to this paper?

If you can answer no to all of the above, write 'I declare that I have no competing interests' below. If your reply is yes to any, please give details below.

I declare that I have no competing interests

I agree to the open peer review policy of the journal. I understand that my name will be included on my report to the authors and, if the manuscript is accepted for publication, my named report including any attachments I upload will be posted on the website along with the authors' responses. I agree for my report to be made available under an Open Access Creative Commons CC-BY license (http://creativecommons.org/licenses/by/4.0/). I understand that any comments which I do not wish to be included in my named report can be included as confidential comments to the editors, which will not be published.

Choose an item.

To further support our reviewers, we have joined with Publons, where you can gain additional credit to further highlight your hard work (see: https://publons.com/journal/530/gigascience). On publication of this paper, your review will be automatically added to Publons, you can then choose whether or not to claim your Publons credit. I understand this statement.

Yes Choose an item.
